# Branch-Selective Alkene Hydroarylation by Cooperative Destabilization: Iridium-Catalyzed *ortho*-Alkylation of Acetanilides

**DOI:** 10.1002/anie.201506581

**Published:** 2015-10-22

**Authors:** Giacomo E M Crisenza, Olga O Sokolova, John F Bower

**Affiliations:** School of Chemistry, University of Bristol Bristol, BS8 1TS (United Kingdom) E-mail: john.bower@bris.ac.uk

**Keywords:** acetanilides, branch selectivity, hydroarylation, iridium, phosphine ligands

## Abstract

An iridium(I) catalyst system, modified with the wide-bite-angle and electron-deficient bisphosphine d^F^ppb (1,4-bis(di(pentafluorophenyl)phosphino)butane) promotes highly branch-selective hydroarylation reactions between diverse acetanilides and aryl- or alkyl-substituted alkenes. This provides direct and *ortho*-selective access to synthetically challenging anilines, and addresses long-standing issues associated with related Friedel–Crafts alkylations.

Anilines are privileged building blocks for medicinal chemistry and materials science,[1] and many methods have been developed to access substituted derivatives.[2–7] However, a long-standing deficiency resides in the lack of general procedures for the *ortho*-selective introduction of branched alkyl substituents. Palladium-catalyzed cross-couplings of secondary alkyl organometallics are not well suited to this task, partially because of competitive isomerization after transmetalation, which can lead to linear adducts.[8] *ortho*-Selective Friedel–Crafts reactions are an appealing approach, which, in practice, is effective only in certain simple cases.[9] Well established problems associated with controlling the site and extent of alkylation usually predominate, and competitive coordination of the acid catalyst to the aniline nitrogen atom means that, where feasible, harsh reaction conditions are required.[9] Consequently, a method that addresses these issues by providing direct and mild access to *ortho*-branched anilines is likely to have widespread application.

Recently, we reported a method that overturns the linear selectivity of Murai-type hydroarylations[10, 11] to provide access to branched adducts **1 b** (Scheme 1 a).[12, 13] All steps up to iridium(III)–alkyl intermediates **3 a** and **3 b** are fast and reversible, with linear adduct **3 a** likely favored on steric grounds. Our “cooperative destabilization” strategy employs a novel bisphosphine, d^F^ppb, with a wide bite angle to increase bond angle *y* and compress angles *x*^*a*^/*x*^*b*^, thereby enhancing steric destabilization of **3 a**/**3 b**.[12] Destabilization is most acute for **3 b**, as this has a bulkier secondary alkyl ligand, and consequently, reductive elimination by path b is amplified to provide branched products **1 b** at the expense of linear isomers **1 a**.[14] This process employs weakly coordinating carbonyl directing groups, and tolerates both aryl- and alkyl-substituted alkenes. Related branch-selective hydroarylation methods invariably require strongly coordinating N-based directing groups and are limited to styrenes[15a–d] or, more recently, enol ethers as the olefinic partner.[15e, 16, 17] In this report, we extend our strategy to the branch-selective *ortho*-alkylation of acetanilides (Scheme 1 b).[18] Significantly, this work expands our approach to encompass a) electron-rich arenes, and b) inherently more demanding six-ring metallacycles (**2** vs. **4**). Indeed, to the best of our knowledge, this study outlines the first intermolecular branch-selective Murai-type alkene hydroarylations that proceed via six-ring chelates. In combination with earlier work,[12] these results suggest that a unified approach to branch-selective alkene hydroarylation is achievable and underpin ongoing efforts towards enantioselective variants.

**Scheme 1:**
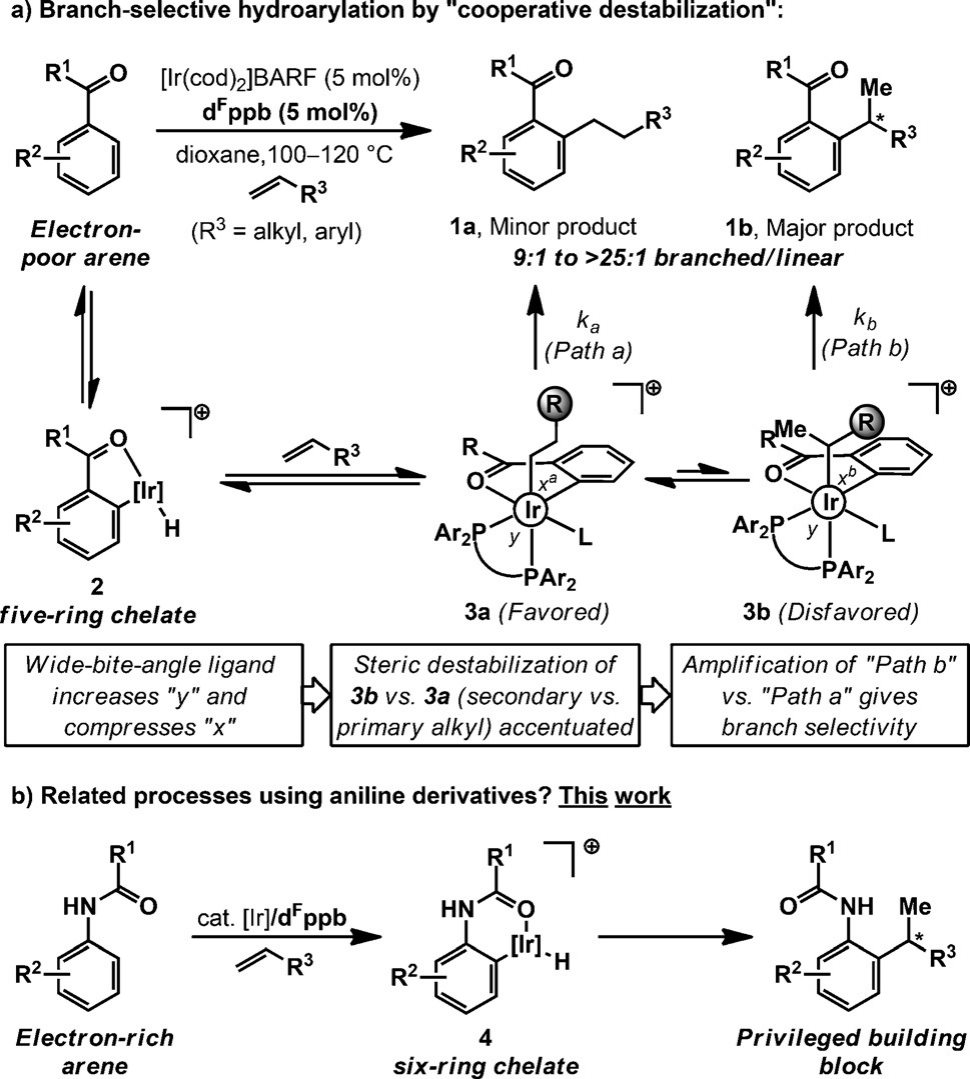
Branch-selective hydroarylation by “cooperative destabilization” and outline of this work. BARF=tetrakis(3,5-bis(trifluoromethyl)phenyl)borate, cod=1,5-cyclooctadiene, d^F^ppb=1,4-bis(di(pentafluorophenyl)phosphino)butane.

Preliminary studies involved exposing acetanilide **5 a** and styrene to an Ir^I^ system derived from [Ir(cod)_2_]BARF and d^F^ppb (Table 1). At 120 °C in dioxane, adduct **6 a** was formed in 34 % yield and with complete branch selectivity (entry 1). Lower reaction temperatures or longer reaction times resulted in diminished yields of **6 a** (entries 2–4). However, a strong dependence upon the Ir counterion was observed, and increased yields were achieved using more strongly associating variants. This resulted in the conditions in entry 6, which deliver **6 a** in 85 % yield and, importantly, with complete branch selectivity and full selectivity for mono-*ortho*-alkylation. Higher loadings of styrene offered no appreciable benefit (entry 6 vs. entry 7). Solvents other than dioxane can be employed, but marginally lower yields of **6 a** were obtained (entries 8–11). Interestingly, for reactions run in 1,2-dichlorobenzene (1,2-DCB), the BARF counterion was superior to triflate (entry 8 vs. entry 9). The secondary acetamide directing group of **5 a** is crucial to the process and the corresponding N-methylated derivative (entry 12) as well as carbamate-, tosyl-, formyl-, and pivaloyl-protected substrates were all ineffective.[19]

**Table 1:** Selected optimization results

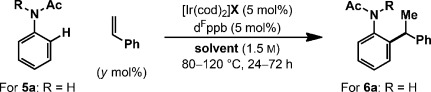							

Entry	R	X	*y* [mol %]	Solvent	*T* [°C]	*t* [h]	Yield [%]^[a]^
1	H	BARF	200	dioxane	120	24	34
2	H	BARF	200	dioxane	100	24	17
3	H	BARF	200	dioxane	80	24	<5
4	H	BARF	200	dioxane	120	72	22^[b]^
5	H	BF_4_	200	dioxane	120	24	32
**6**	**H**	**OTf**	**200**	**dioxane**	**120**	**24**	**85**
7	H	OTf	450	dioxane	120	24	85
8	H	OTf	200	1,2-DCB	120	24	61
9	H	BARF	200	1,2-DCB	120	24	72
10	H	OTf	200	xylene	120	24	58^[b]^
11	H	OTf	200	PhCl	120	24	60^[b]^
12	CH_3_	OTf	200	dioxane	120	24	<5

[a] Yields of isolated products unless stated otherwise, >25:1 branched/linear in all cases. [b] Determined by ^1^H NMR analysis with 1,3,5-trimethoxybenzene as the internal standard. Tf=trifluoromethanesulfonyl.

The scope of the aniline component is outlined in Table 2. Hydroarylation of styrene with *ortho*-substituted derivatives **5 b**–**5 e** provided target compounds **6 b**–**6 e** in high yield and with complete branch selectivity in all cases. For optimal efficiencies, fine-tuning of the styrene loading was required on a case by case basis. For example, hydroarylation to afford **6 b** occurred in only 54 % yield with 200 mol % styrene, but a 73 % yield was achieved using 450 mol %. The tolerance to *ortho*-substitution contrasts our earlier work with aryl ketones and benzamides, where analogous processes were not feasible.[12] Lower loadings of styrene can be used for substrates with electron-donating groups at the C3 position. For example, **6 c** and **6 d** were both generated in excellent yield using just one equivalent of the alkene. Aniline derivatives **5 f**–**5 j** have two different *ortho* C–H bonds available, and regioselectivity is strongly influenced by the *meta*-substituent. For **5 f**–**5 h**, hydroarylation occurred preferentially at the less hindered site to afford adducts **6 f**–**6 h** (4:1 to >25:1 *ortho*-regioselectivity); the structures of **6 h** and *iso*-**6 h** were determined by single-crystal X-ray diffraction.[20] For **5 i** and **5 j**, hydroarylation was moderately selective for the *ortho* C–H bond adjacent to the heteroatom substituent.[21] *para*-Substituted anilines **5 k** and **5 m** participated smoothly, and products **6 k** and **6 m** were formed in good yield. Conversely, hydroarylation using *para*-trifluoromethyl derivative **5 l** was not efficient, and adduct **6 l** was formed in 21 % yield. Complete branch selectivity and complete selectivity for mono-*ortho*-alkylation (>95:5 mono/bis) were observed for **6 f**–**6 m**.

**Table 2:** Aniline scope^[a]^

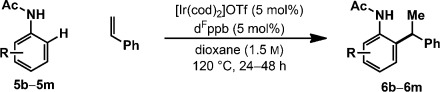		
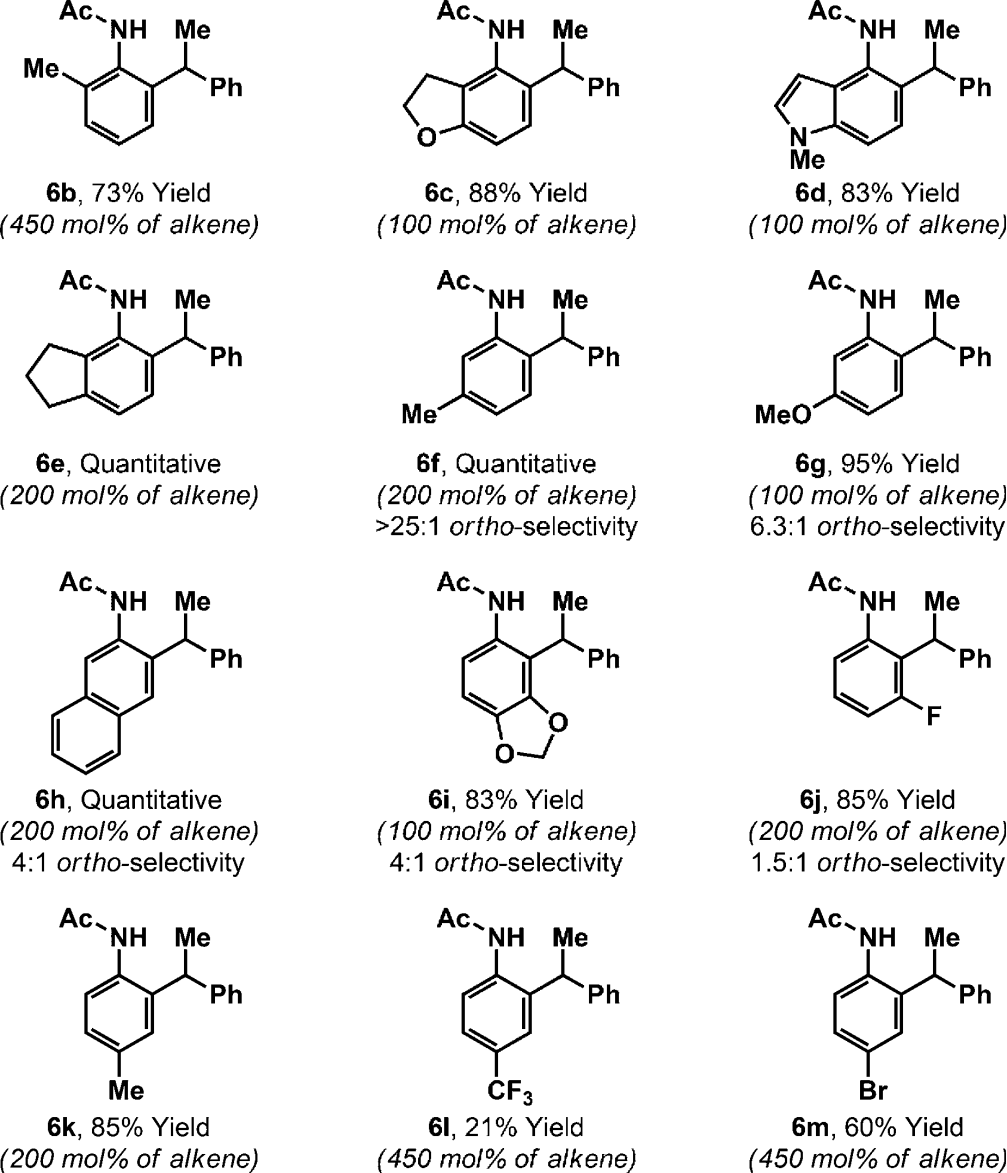		

[a] In all cases, branched/linear >25:1.

We have also examined the scope of the alkene component using acetanilide **5 f**, and, again, complete branch selectivity was achieved in all cases (Table 3). Electronically diverse styrenes are well tolerated, and the target compounds **7 a**–**7 e** were formed in moderate to quantitative yield, with complete selectivity for mono-alkylation at the less hindered *ortho*-site. Processes involving alkyl-substituted alkenes required separate optimization. Changing the precatalyst counterion from triflate to BARF and switching the solvent from dioxane to 1,2-DCB provided a system that delivered targets **7 f**–**7 i** in 33–99 % yield, albeit with 600 mol % of the alkene component. For **7 g**, propylene gas was delivered at atmospheric pressure to introduce the *ortho*-isopropyl moiety in 92 % yield. Isopropyl groups are challenging to install using Pd-catalyzed cross-couplings,[8a] and the present method provides a direct and atom-economic alternative. Sterically demanding alkenes are challenging, and the conversion of **5 f** into **7 h** occurred in only 33 % yield; however, even here, branch selectivity was maintained.

**Table 3:** Alkene scope

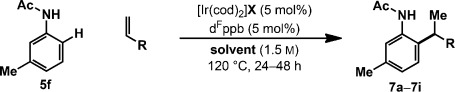		
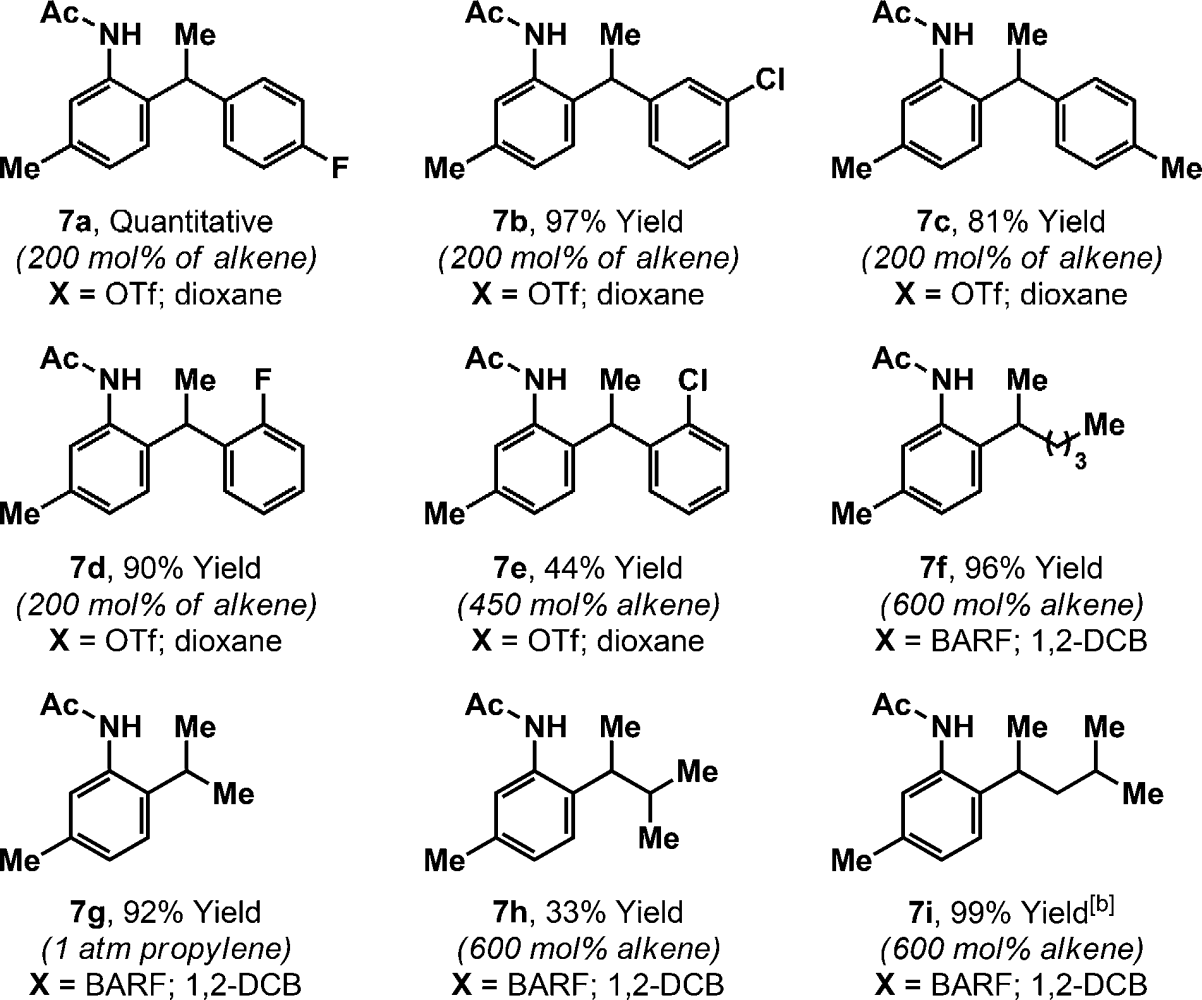		

[a] In all cases, branched/linear and *ortho*-selectivity >25:1. [b] 7.5 mol % of [Ir(cod)_2_]BARF and d^F^ppb.

The mechanism of the hydroarylation process is likely analogous to that outlined in our earlier work (Scheme 1 a).[12] Hydroarylation of [D_2_]-**8** with aniline **5 f** delivered deuterated **7 c**, in which deuterium incorporation at both the methyl and methine positions indicates reversible alkene hydrometalation prior to product-determining C–C bond formation (Scheme 2). The lack of deuterium incorporation at the C6 position suggests that C–H insertion of the Ir catalyst is, in this case, selective for the more sterically accessible *ortho* C–H bond. Indeed, exposure of aniline **5 f** to the Ir system in the absence of the alkene, but in the presence of D_2_O, resulted in 92 % deuterium incorporation at the C6 position and in <5 % at the C2 position; further exchange experiments are outlined in the Supporting Information. The X-ray structures of **6 h** and *iso*-**6 h**[20] show that the secondary alkyl substituent of the products causes the acetamide moiety to twist from the plane of the arene, such that directed insertion of Ir^I^ into the remaining *ortho* C–H bond is challenging; consequently, bis-*ortho*-alkylation is not observed. This effect must be finely balanced given that *ortho*-substituted acetanilides **5 b**–**5 e** participate smoothly in the hydroarylation reaction.

**Scheme 2:**
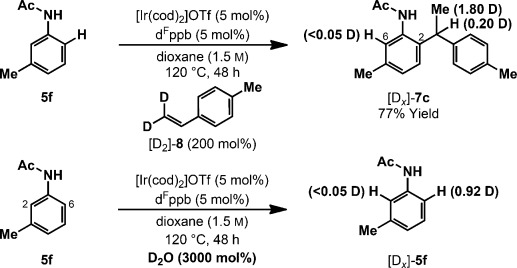
Deuterium labeling and exchange experiments.

A key feature of the processes described here is the use of the wide-bite-angle and electron-deficient bisphosphine ligand d^F^ppb. The branched/linear selectivity for **5 a** to **6 a**/*iso*-**6 a** has been evaluated as a function of ligand bite angle (Scheme 3).[22] A progression from low linear to complete branch selectivity is observed as the ligand is varied from d^F^ppm to d^F^ppb. Although a strong bite-angle effect is evident, a significant electronic influence is also operative. Non-fluorinated ligands, namely dppm, dppe, dppp, and dppb, show the same bite-angle trend, but provide lower branch selectivities. One explanation is that secondary alkyl ligands are better able to stabilize a more electron-deficient Ir center, and so the equilibrium branched/linear ratio of the alkyl–Ir^III^ intermediates (see **3 b** vs. **3 a**) increases when fluorinated ligands are used.[23, 24] Another option is that electron-deficient ligands a) shorten the iridium–alkyl bond by enhancing σ-donation, and b) shorten the iridium–phosphine bonds by increasing π-backbonding.[25] This results in a contraction of the coordination sphere to provide a more congested environment, such that steric destabilization of the branched alkyl–Ir^III^ intermediate is amplified further, and its propensity for reductive elimination increases.

**Scheme 3:**
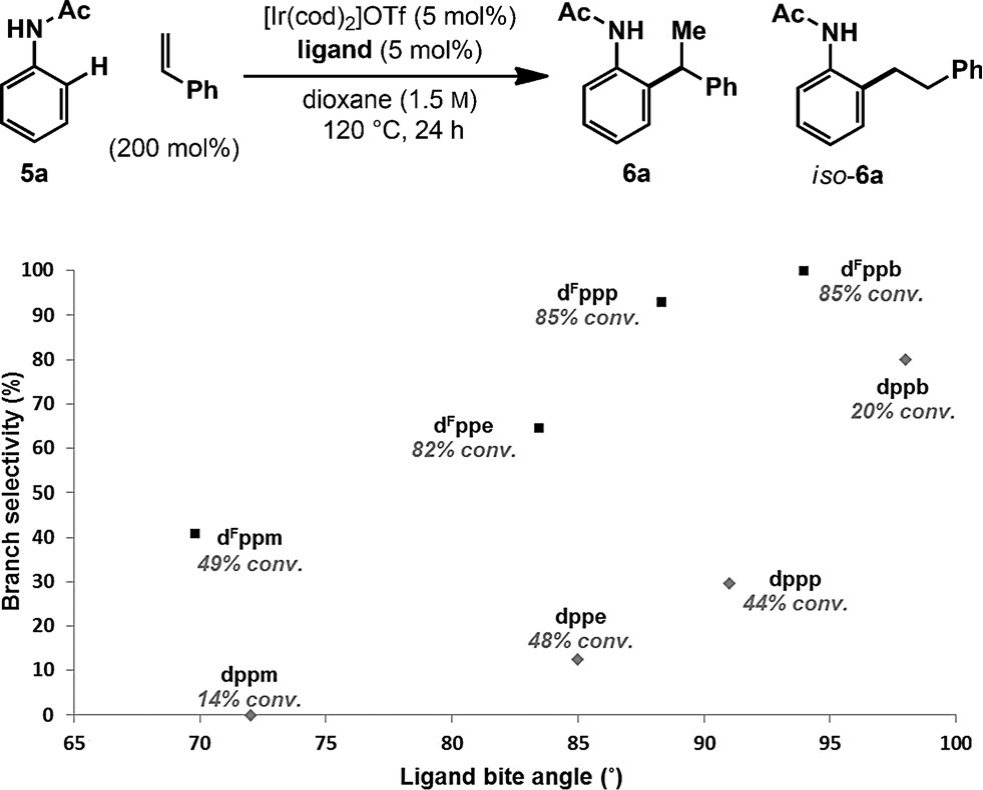
Ligand effects for the hydroarylation of styrene with 5 a.

The substituted aniline products enable access to a wide range of challenging bicyclic heteroaromatic compounds (Scheme 4). Pd-catalyzed *ortho*-bromination of **6 a**, which was prepared on gram scale, delivered **10** in 88 % yield and excellent selectivity.[26] Pd-catalyzed reaction of **10** with *ortho*-toluidine provided benzimidazole **11** in 89 % yield.[27] In this approach, the *N*-acetyl group is incorporated into the heteroaromatic target. Other transformations required conversion into aniline **12**.[28] This intermediate underwent condensation with ethyl acetoacetate, and subsequent Pd-catalyzed oxidative cyclization delivered indole **13** in 52 % yield (over 2 steps).[29] Classical methods towards heteroaromatic compounds are also effective. For example, the Cohn variant of the Skraup quinoline synthesis delivered **14** in 68 % yield.[30] The processes in Scheme 4 validate concise and diversifiable entries to heteroaromatic targets that might be difficult to prepare by other means.

**Scheme 4:**
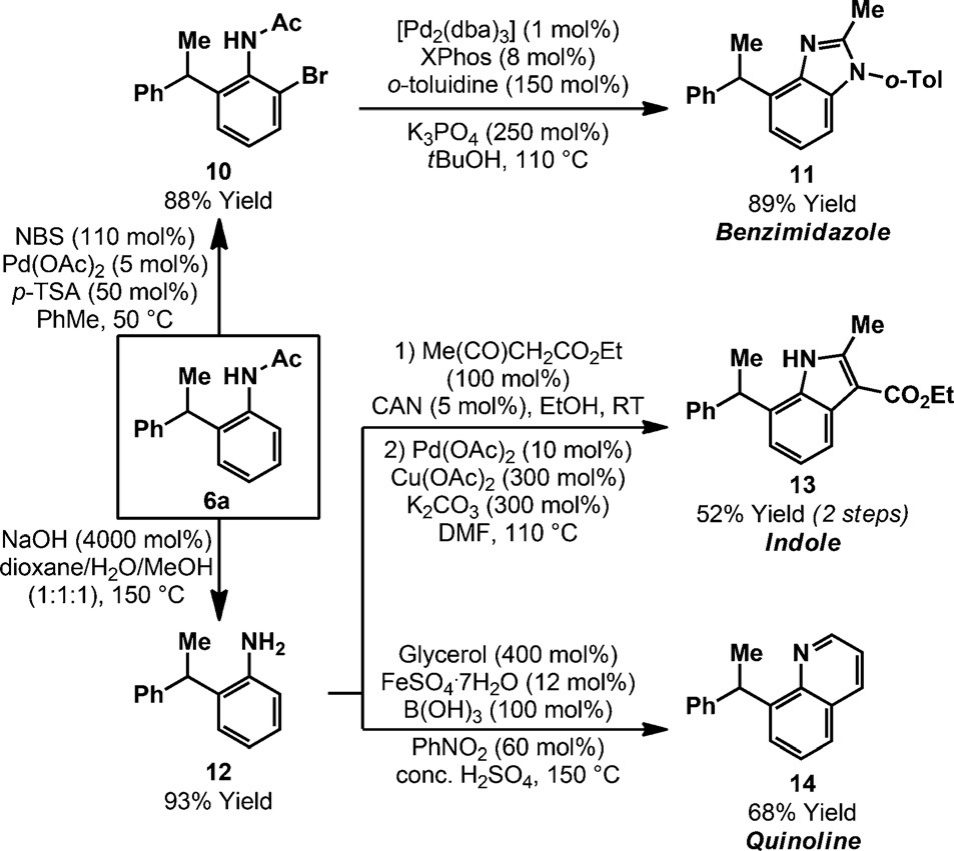
Product derivatizations. dba=dibenzylideneacetone, CAN=cerium ammonium nitrate, NBS=*N*-bromosuccinimide, *p*-TSA=*para*-toluenesulfonic acid.

To conclude, we outline a direct and controlled approach to *ortho*-branched aniline derivatives, which addresses long-standing issues associated with related Friedel–Crafts alkylations. More fundamentally, this work extends our “cooperative destabilization” strategy[12] to include processes that involve electron-rich arenes and proceed via six-ring metallacycles. Both aspects represent a significant expansion to the emerging area of branch-selective Murai-type hydroarylations.[12, 15] The catalyst design features used here will guide efforts in our laboratory aimed at developing a general and enantioselective alkene hydroarylation method.

## Supplementary material

As a service to our authors and readers, this journal provides supporting information supplied by the authors. Such materials are peer reviewed and may be re-organized for online delivery, but are not copy-edited or typeset. Technical support issues arising from supporting information (other than missing files) should be addressed to the authors.
