# Supplementary material for: Branch-Selective Alkene Hydroarylation by Cooperative Destabilization: Iridium-Catalyzed ortho-Alkylation of Acetanilides
Source: Angew Chem Int Ed Engl. 2015 Oct 22;54(49):14866–70. doi: 10.1002/anie.201506581 (PMC4691333; doi:10.1002/anie.201506581)
Supplement: Supplementary file 1 [file anie0054-14866-SD1.pdf]

Angewandte Chemie
Eine Zeitschrift der Gesellschaft Deutscher Chemiker

Supporting Information

Branch-Selective Alkene Hydroarylation by Cooperative Destabilization: Iridium-Catalyzed *ortho*-Alkylation of Acetanilides

*Giacomo E. M. Crisenza, Olga O. Sokolova, and John F. Bower**

anie_201506581_sm_miscellaneous_information.pdf

Supporting Information

Table of Contents

General Experimental Details 2
Experimental Procedures and Data 3
  Substrate Synthesis 3
  Reaction Scope 7
  Mechanistic Studies 24
  Ligand Bite Angle and Electronic Effects 27
  Product Derivatizations 30
Copies of ^{1}H and ^{13}C NMR 35
References 68

General Experimental Details

All reagents requiring purification were purified using standard laboratory techniques according to methods published by Perrin, Armarego, and Perrin (Pergamon Press, 1966). Catalytic reactions were carried out in Young-type re-sealable tubes. Styrenes were distilled before use, while alkyl-substituted alkenes were used without any further purification. Iridium catalysts, 4-methylstyrene-*β*,*β*-d_2 and per-fluorinated ligands were synthesized according to previous reported procedures or purchased from commercial sources.[1] Anhydrous solvents were obtained by distillation using standard procedures or by passage through drying columns supplied by Anhydrous Engineering Ltd. Anhydrous 1,4-dioxane was sparged with argon for 10 minutes prior use. Flash column chromatography (FCC) was performed using silica gel (Aldrich 40-63 µm, 230-400 mesh). Thin layer chromatography was performed using aluminium backed 60 F_{254} silica plates. Visualization was achieved by UV fluorescence or a basic $KMnO_4$ solution and heat. Proton nuclear magnetic resonance spectra (NMR) were recorded at 400 MHz or 500 MHz as stated. ^{13}C NMR spectra were recorded at 100 MHz or 125 MHz as stated. Chemical shifts (δ) are given in parts per million (ppm). Peaks are described as singlets (s), doublets (d), triplets (t), quartets (q), septets (sept), multiplets (m) and broad (br.). Coupling constants (*J*) are quoted to the nearest 0.5 Hz. All assignments of NMR spectra were based on 2D NMR data (DEPT^{135}, COSY, HSQC and HMBC). Where compounds were isolated as a mixture of isomers (*e.g.* regioisomers, rotamers), they are referred as *A* and *B. In situ* yields were determined by employing 1,3,5-trimethoxybenzene as an internal standard. Mass spectra were recorded using a VG Autospec (CI$_+$ mode), a Brüker Daltonics FT-ICR-MS Apex 4e 7.0T FT-MS (ESI$_+$ mode) and Shimadzu GCMS QP2010$_+$ (EI$_+$ mode). Infrared spectra were recorded on a Perkin Elmer Spectrum Two FTIR spectrometer as thin films or solids compressed on a diamond plate. Melting points were determined using Reichert melting point apparatus and are uncorrected.

Experimental Procedures and Data

Substrate Synthesis

General Procedure A for the preparation of acetanilide substrates from anilines: To an ice-cooled (0 °C) solution of aniline (100 mol%) and pyridine (60 mol%) in CH_2Cl_2 (0.7 M) was added dropwise acetic anhydride (110 mol%). The reaction was warmed to r.t. and stirred until consumption of the starting material (monitored by TLC). Water (2 mL/mmol) was added and the mixture was extracted with CH_2Cl_2 (3 × 5 mL/mmol). The organic extracts were combined, washed with saturated aq. $NaHCO_3$ (3 × 2 mL/mmol) and brine (2 mL/mmol), dried over Na_2SO_4 and concentrated *in vacuo* to provide the crude product. Purification by either FCC (40% EtOAc/hexane → 50% EtOAc/hexane) or recrystallization (hexane/EtOAc) afforded pure acetanilide.

*Substrates **5a**, **5b**, **5f**, **5j**, **5k**, **5l** and **5m** were purchased from commercial sources (Aldrich, Alfa-Aesar) and used without any further purification.*

Preparation of substrate 5c [2,3-Dihydro-4-benzofuranacetanilide]:

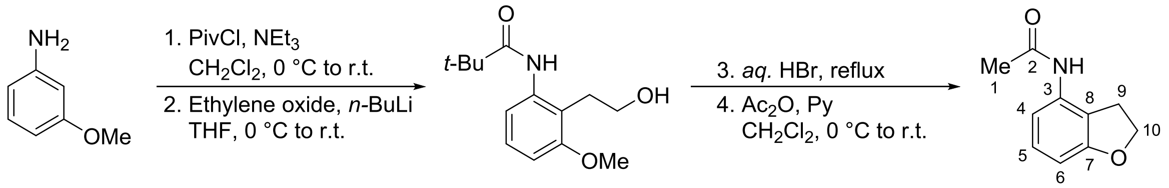

Step 1: To an ice-cooled (0 °C) solution of *m*-anisidine (1.08 mL, 9.65 mmol, 100 mol%) and NEt_3 (1.61 mL, 11.6 mmol, 120 mol%) in CH_2Cl_2 (25 mL) was added dropwise pivaloyl chloride (1.43 mL, 11.6 mmol, 120 mol%). The reaction was warmed to r.t. and stirred until consumption of the starting material was observed (TLC). The reaction was quenched with aq. 1M HCl (15 mL) and the mixture was extracted with CH_2Cl_2 (3 × 25 mL). The organic extracts were combined, washed with brine (10 mL), dried over Na_2SO_4 and concentrated *in vacuo* to provide the crude product (2.00 g, quantitative yield). The crude material was employed without any further purification on the next step. ^1H NMR ($CDCl_3$, 400 MHz): δ 7.38 (1H, t, J = 2.5 Hz), 7.29 (1H, br. s), 7.19 (1H, t, J = 8.0 Hz), 6.92 (1H, ddd, J = 1.0, 2.5, 8.0 Hz), 6.65 (1H, ddd, J = 1.0, 2.5, 8.0 Hz), 3.80 (3H, s), 1.31 (9H, s); ^{13}C NMR ($CDCl_3$, 100 MHz): δ 176.5, 160.2, 139.3, 129.5, 111.8, 110.4, 105.3, 55.3, 39.7, 27.6. *The spectroscopic proprieties were consistent with the data available in literature.*[2a]

Step 2: To an ice-cooled (0 °C) solution of *N*-(3-methoxyphenyl)pivalamide (1.90 g, 9.17 mmol, 100 mol%) in anhydrous THF (35 mL) was added dropwise a 1.5 M solution of *n*-BuLi (22.9 mmol, 250 mol%) in hexanes. The reaction was stirred at 0 °C for 2 hours. Then a 2.5 M solution of ethylene oxide (13.7 mmol, 150 mol%) in THF was added to the mixture and the reaction was stirred at 0 °C for an additional hour. The solution was warmed to r.t. and stirred overnight. Water (5 mL) was added and the solvent was removed *in vacuo*. The residue was re-suspended in water (10 mL) and extracted

with EtOAc (3 × 20 mL). The organic extracts were combined, washed with saturated aq. Na_2CO_3 (5 mL), dried over Na_2SO_4 and concentrated *in vacuo* to provide the crude material. Purification by FCC (10% EtOAc/hexane → 30% EtOAc/hexane) afforded pure product (1.87 g, 81% yield) as a colorless solid. ^{1}H NMR ($CDCl_3$, 400 MHz): δ 8.79 (1H, br. s), 7.44 (1H, dd, *J* = 1.0, 8.0 Hz), 7.19 (1H, t, *J* = 8.0 Hz), 6.67 (1H, dd, *J* = 1.0, 8.0 Hz), 3.91 (2H, dt, *J* = 4.0, 5.5 Hz), 3.79 (3H, s), 2.87 (2H, t, *J* = 5.5 Hz), 1.30 (9H, s); ^{13}C NMR ($CDCl_3$, 100 MHz): δ 177.1, 157.5, 138.2, 127.1, 120.3, 116.9, 106.7, 63.8, 55.6, 39.6, 27.7, 27.1. *The spectroscopic proprieties were consistent with the data available in literature.*[2b]

Step 3: A round-bottomed flask equipped with a condenser was charged with *N*-(2-(2-hydroxyethyl)-3-methoxyphenyl)pivalamide (1.86 g, 7.40 mmol) and 48% aq. HBr (20 mL). The mixture was heated at 100 °C overnight. The reaction was cooled to r.t. and the pH of the solution was adjusted to ≈ 9 with NaOH pellets. The mixture was extracted with EtOAc (3 × 20 mL). The organic extracts were combined, washed with water (10 mL), dried over Na_2SO_4 and concentrated *in vacuo* to provide the crude aniline product (1.06 g, quantitative yield). The crude material was employed in the next step without any further purification. ^{1}H NMR ($CDCl_3$, 400 MHz): δ 6.93 (1H, t, *J* = 8.0 Hz), 6.26 (1H, dd, *J* = 1.0, 8.0 Hz), 6.22 (1H, dd, *J* = 1.0, 8.0 Hz), 4.58 (2H, t, *J* = 8.5 Hz), 3.57 (2H, br. s), 3.02 (2H, t, *J* = 8.5 Hz). *The spectroscopic proprieties were consistent with the data available in literature.*[2b]

Step 4: This step was accomplished following General Procedure A. Purification by recrystallization (hexane/EtOAc) provided substrate **5c** in 71% yield (58% yield over 4 steps) as an off-white solid; ν_{max} / cm^{-1}: 3441 (s), 3002 (m), 2984 (s), 2908 (m), 1687 (s), 1609 (s), 1524 (s), 1437 (s), 1096 (s); ^{1}H NMR ($CDCl_3$, 400 MHz): δ 7.36 (1H, br. s, N-H), 7.19-6-91 (2H, m, C**4**-H and C**5**-H), 6.57 (1H, d, *J* = 7.5 Hz, C**6**-H), 4.51 (2H, t, *J* = 8.5 Hz, C**10**-H_2), 3.06 (2H, t, *J* = 8.5 Hz, C**9**-H_2), 2.12 (3H, s, C**1**-H_3); ^{13}C NMR ($CDCl_3$, 100 MHz): δ 168.4 (C**2**), 160.8 (C**7**), 134.3 (C**3**), 128.6 (C**5**), 119.2 (C**8**), 114.3 (C**4**), 106.3 (C**6**), 71.1 (C**10**), 28.3 (C**9**), 24.0 (C**1**); HRMS: (ESI$^+$) Calculated for $C_{10}H_{11}NNaO_2$: 200.0682. Found $[M+Na]^+$: 200.0692; m.p. = 126-127 ˚C (hexane/EtOAc).

Preparation of substrate 5d [*N*-(1-Methyl-1*H*-indol-4-yl)acetamide]:

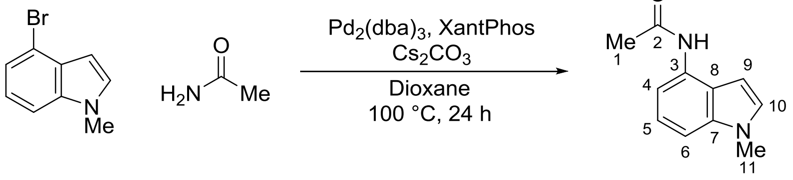

The title compound was prepared following a literature procedure.[3] An oven-dried re-sealable tube, fitted with a magnetic stirrer, was charged with 4-bromo-1-methylindole (500 mg, 2.38 mmol, 100 mol%), acetamide (169 mg, 2.86 mmol, 120 mol%), $Pd_2(dba)_3$ (54.5 mg, 0.06 mmol, 2.50 mol%), Xantphos (103 mg, 0.18 mmol, 7.50 mol%) and Cs_2CO_3 (1.09 g, 3.33 mmol, 140 mol%). The tube was fitted with a rubber septum and purged with nitrogen. Anhydrous 1,4-dioxane (5.70 mL) was

added *via* syringe and the tube was sealed with a Young's tap. The reaction vessel was placed into a pre-heated heating block at 100 °C and stirred for 24 hours. The reaction mixture was cooled to room temperature, diluted with CH_2Cl_2 and filtered through a short pad of Celite®. The solvent was removed under reduced pressure to provide the crude material. Purification by FCC (70% EtOAc/hexane), followed by recrystallization (EtOAc), afforded product **5d** (315 mg, 70% yield) as an off-white solid; ν_{max} / cm^{-1}: 3262 (s), 3151 (m), 3067 (m), 2915 (m), 1650 (s), 1544 (s), 1415 (s), 1279 (s); ^1H NMR (CD_3OD, 400 MHz): δ 7.41 (1H, d, *J* = 8.0 Hz, C**6**-H), 7.19 (1H, d, *J* = 8.0 Hz, C**4**-H), 7.15-7.09 (2H, m, C**5**-H and C**10**-H), 6.55 (1H, d, *J* = 3.0 Hz, C**9**-H), 3.78 (3H, s, C**11**-H_3), 2.20 (3H, s, C**1**-H_3); ^{13}C NMR (CD_3OD, 100 MHz): δ 170.5 (C**2**), 137.8 (C**7**), 129.6 (C**3**), 128.1 (C**10**), 121.9 (C**8**), 121.0 (C**5**), 112.3 (C**6**), 106.1 (C**4**), 97.7 (C**9**), 31.6 (C**11**), 22.1 (C**1**); HRMS: (ESI^+) Calculated for $C_{11}H_{13}N_2O$: 189.1022. Found $[M+H]^+$: 189.1019; m.p. = 205-206 ˚C (EtOAc).

***N*-(2,3-Dihydro-1*H*-inden-4-yl)acetamide (5e)**

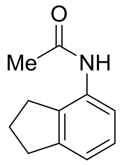

General Procedure A: Purification by recrystallization afforded acetanilide **5e** (439 mg, 2.50 mmol, quantitative yield) as an off-white solid; ^1H NMR ($CDCl_3$, 400 MHz): δ 7.72 (1H, d, *J* = 7.5 Hz), 7.14 (1H, dd, *J* = 7.5, 7.5 Hz), 7.01 (1H, d, *J* = 7.5 Hz), 6.95 (1H, br. s), 2.94 (2H, t, *J* = 7.5 Hz), 2.80 (2H, t, *J* = 7.5 Hz), 2.18 (3H, s), 2.10 (2H, tt, *J* = 7.5, 7.5 Hz); ^{13}C NMR ($CDCl_3$, 100 MHz): δ 168.1, 145.2, 133.9, 133.8, 127.2, 120.8, 118.9, 33.2, 30.0, 24.8, 14.5; m.p. = 125-126 ˚C [hexane/EtOAc] (Lit.[4a] 118-119 ˚C, benzene). *The spectroscopic proprieties were consistent with the data available in literature.*[4b]

***N*-(3-Methoxyphenyl)acetamide (5g)**

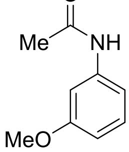

General Procedure A: Purification by FCC afforded acetanilide **5g** (500 mg, 3.03 mmol, quantitative yield) as an off-white solid; ^1H NMR ($CDCl_3$, 400 MHz): δ 8.23 (1H, br. s), 7.27 (1H, s), 7.16 (1H, dd, *J* = 8.0, 8.0 Hz), 7.01 (1H, d, *J* = 8.0 Hz), 6.63 (1H, d, *J* = 8.0 Hz), 3.73 (3H, s), 2.13 (3H, s); ^{13}C NMR ($CDCl_3$, 100 MHz): δ 169.1, 160.0, 139.3, 129.6, 112.3, 109.9, 105.9, 55.2, 24.5; m.p. = 81-83 ˚C [hexane/EtOAc] (Lit.[5] 79-82 ˚C, *no recrystallization solvent specified*). *The spectroscopic proprieties were consistent with the data available in literature.*[5]

Preparation of substrate 5h [*N*-(Naphthalen-2-yl)acetamide]:

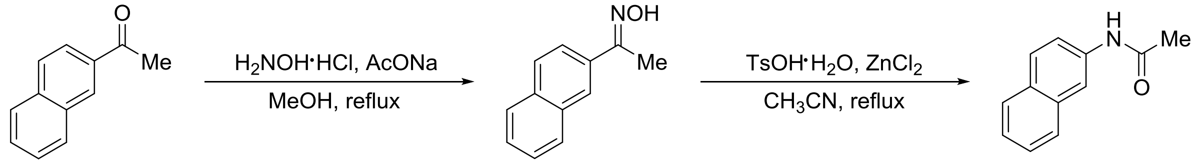

__Step 1__: A round-bottomed flask equipped with a condenser was charged with 2-acetonaphthone (919 mg, 5.40 mmol, 100 mol%), AcONa (531 mg, 6.48 mmol, 120 mol%), $H_2NOH{\cdot}HCl$ (450 mg, 6.48 mmol, 120 mol%) and MeOH (5 mL). The mixture was heated to 70 °C and stirred for 2 hours. The reaction was cooled to r.t. and diluted with toluene (10 mL) and aq. 2M NaOH solution (3 mL). The solvent was removed under reduced pressure. The residue was re-suspended in water (8 mL) and extracted with EtOAc (3 × 15 mL). The organic extracts were combined, washed with brine (5 mL), dried over Na_2SO_4 and concentrated *in vacuo* to provide the crude oxime product (992 mg, 99% yield). The crude material was employed in the next step without any further purification. ^{1}H NMR ($CDCl_3$, 400 MHz): δ 8.02 (1H, s), 7.91-7.80 (4H, m), 7.68 (1H, br. s), 7.53-7.46 (2H, m), 2.41 (3H, s). *The spectroscopic proprieties were consistent with the data available in literature.*[6a]

__Step 2__: A round-bottomed flask equipped with a condenser was charged with 1-(naphthalen-2-yl)ethan-1-one oxime (991 mg, 5.35 mmol, 100 mol%), TsOH (102 mg, 0.53 mmol, 10.0 mol%), $ZnCl_2$ (87.2 mg, 0.64 mmol, 12.0 mol%) and CH_3CN (8 mL). The mixture was heated at reflux for 5 hours. The reaction was cooled to r.t. and aq. 2M NaOH solution (6 mL) was added to the mixture. The solvent was removed under reduced pressure. The residue was re-suspended in water (10 mL) and extracted with EtOAc (3 × 25 mL). The organic extracts were combined, washed with brine (5 mL), dried over Na_2SO_4 and concentrated *in vacuo* to provide the crude material. Purification by FCC (30% EtOAc/hexane → 50% EtOAc/hexane) afforded the acetanilide product **5h** (824 mg, 83% yield) as an off-white solid. ^{1}H NMR ($CDCl_3$, 400 MHz): δ 8.18 (1H, s), 7.91 (1H, br. s), 7.84-7.61 (3H, m), 7.54-7.32 (3H, m), 2.20 (3H, s); ^{13}C NMR ($CDCl_3$, 100 MHz): δ 168.9, 135.4, 133.8, 130.6, 128.7, 127.6, 127.5, 126.4, 125.0, 120.0, 116.8, 24.6; m.p. = 131-133 ˚C [hexane/EtOAc] (Lit.[6b] 133-134 ˚C, EtOH). *The spectroscopic proprieties were consistent with the data available in literature.*[6c]

***N*-(Benzo[*d*][1,3]dioxol-5-yl)acetamide (5i)**

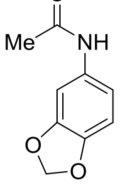

__General Procedure A:__ Purification by recrystallization afforded acetanilide **5i** (344 mg, 1.92 mmol, 69% yield) as a brown solid; ^{1}H NMR ($CDCl_3$, 400 MHz): δ 7.20 (1H, s), 7.15 (1H, br. s), 6.81-6.69 (2H, m), 5.94 (2H, s), 2.14 (3H, s); ^{13}C NMR ($CDCl_3$, 100 MHz): δ 168.1, 147.8, 144.3, 132.01,

113.2, 108.0, 103.0, 101.2, 24.4; m.p. = 137-138 ˚C [hexane/EtOAc] (Lit.[7a] 133-135 ˚C, petroleum ether/EtOAc). *The spectroscopic proprieties were consistent with the data available in literature.*[7b]

Preparation of substrate 9 [*N*-Methylacetanilide]:

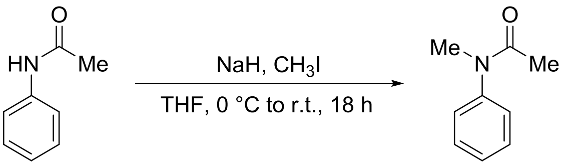

In a two-necked round-bottomed flask, equipped with condenser and N_2 adaptor, NaH (60% dispersion in mineral oil, 1.07 g, 26.8 mmol, 400 mol%) was suspended in anhydrous THF (50 mL). The mixture was cooled 0 °C and a solution of acetanilide (906 mg, 6.70 mmol, 100 mol%) in anhydrous THF (20 mL) was added dropwise. The reaction was stirred at 0 °C for 30 minutes. Then a solution of CH_3I (1.67 mL, 26.8 mmol, 400 mol%) in anhydrous THF (20 mL) was slowly added to the mixture. After complete addition, the reaction was warmed to room temperature and stirred overnight. Water (20 mL) was added and the mixture was extracted with EtOAc (3 × 20 mL). The organic extracts were combined, washed with brine (15 mL), dried over Na_2SO_4 and concentrated *in vacuo* to provide the crude material. Purification by recrystallization (hexane/EtOAc) afforded pure product **9** (726 mg, 73% yield) as an off-white solid. ^{1}H NMR ($CDCl_3$, 400 MHz): δ 7.39 (2H, dd, J = 7.5 Hz), 7.31 (1H, t, J = 7.5 Hz), 7.17 (2H, d, J = 7.5 Hz), 3.24 (3H, s), 1.85 (3H, s); ^{13}C NMR ($CDCl_3$, 100 MHz): δ 170.5, 144.6, 129.7, 127.7, 127.1, 37.1, 22.4; m.p. = 96-97 ˚C [hexane/EtOAc] (Lit.[8a] 93-96 ˚C, hexane). *The spectroscopic proprieties were consistent with the data available in literature.*[8b]

Reaction Scope

<u>General Procedure B</u> for branch selective hydroarylation reactions

An oven-dried re-sealable tube, fitted with a magnetic stirrer, was charged with acetanilide substrate (0.14 mmol, 100 mol%), iridium pre-catalyst (5 mol%) and d^{F}ppb (5.62 mg, 5 mol%). The tube was fitted with a rubber septum and purged with nitrogen. A solution of the appropriate alkene (100-450 mol%) in the appropriate anhydrous solvent (1.5 M concentration with respect to substrate) was added *via* syringe and the tube was sealed with a Young's tap. The reaction vessel was placed into a pre-heated heating block at 120 ºC and stirred for 24-48 hours. The reaction mixture was cooled to room temperature and concentrated *in vacuo*. Purification of the residue by FCC (10% EtOAc/toluene → 30% EtOAc/toluene) afforded pure acetanilide product.

Representative gram-scale alkene hydroarylation procedure An oven-dried re-sealable tube, fitted with a magnetic stirrer, was charged with acetanilide 5a (706 mg, 5.22 mmol, 100 mol%),

[Ir(cod)$_2$]OTf (144 mg, 0.26 mmol, 5 mol%) and d^{F}ppb (205 mg, 0.26 mmol, 5 mol%). The tube was fitted with a rubber septum and purged with nitrogen. A solution of freshly distilled styrene (1.20 mL, 10.5 mmol, 200 mol%) in 1,4-dioxane (3.5 mL) was added *via* syringe and the tube was sealed with a Young's tap. The reaction vessel was placed into a pre-heated heating block at 120 °C and stirred for 72 hours. The reaction mixture was cooled to room temperature and concentrated *in vacuo*. Purification of the residue by flash column chromatography (SiO_2, 10% EtOAc/toluene to 30% EtOAc/toluene) afforded product 6a (1.03 g, 83% yield, >25:1 branched:linear) as an off-white solid. *Characterization data are provided later.*

N-(2-(1-Phenylethyl)phenyl)acetamide (6a)

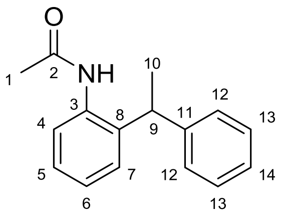

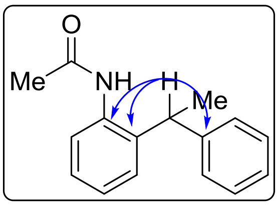

HMBC analysis

General Procedure B: [Ir(cod)$_2$]OTf was employed as the pre-catalyst. A solution of styrene (200 mol%) in anhydrous 1,4-dioxane was added to the reaction tube. The reaction was conducted for 48 hours and afforded acetanilide **6a** (29.2 mg, 85% yield, 0.9:0.1 mixture of rotamers *A*:*B*, >25:1 branched:linear) as an off-white solid; ν_{max} / cm^{-1}: 3410 (s), 3027 (m), 2971 (s), 2933 (m), 1684 (s), 1492 (s), 1376 (s), 1255 (s); ^{1}H NMR ($CDCl_3$, 400 MHz): δ 7.70 (0.9H, d, *J* = 8.0 Hz, C**4**-H, *A*), 7.42 (0.9H, d, *J* = 7.5 Hz, C**7**-H, *A*), 7.37-7.05 (7.2H, m, ArC-H, *A*+*B*), 6.71 (1H, br. s, N-H, *A*+*B*), 4.36-4.23 (0.1H, m, C**9**-H, *B*), 4.16 (0.9H, q, *J* = 7.0 Hz, C**9**-H), 1.94 (2.7H, s, C**1**-H_3, *A*), 1.62 (3.3H, d, *J* = 7.0 Hz, C**10**-H_3 *of A* and C**1**-H_3 + C**10**-H_3 *of B*); ^{13}C NMR ($CDCl_3$, 100 MHz, *major rotamer signals only*): δ 168.3 (C**2**), 145.4 (C**11**), 136.7 (C**8**), 135.3 (C**3**), 129.2 (C1**3**), 127.4 (C**7**), 127.3 (2 signals, C**6** and C**12**), 126.9 (C**14**), 125.6 (C**5**), 125.0 (C**4**), 40.9 (C**9**), 24.1 (C**1**), 21.8 (C**10**); HRMS: (ESI$^+$) Calculated for $C_{16}H_{18}NO$: 240.1383. Found [M+H]$^+$: 240.1376; m.p. = 107-109 ˚C (hexane/CH_2Cl_2). *The regiochemistry of compound **6a** was confirmed by HMBC (as indicated above). Branch selectivity was further confirmed by ^{13}C-DEPT NMR analysis that showed 3 CH/CH_3 signals in the alkyl region. High temperature ^{1}H NMR experiments (90˚C, toluene-d_8) showed coalescence of the signals associated with each rotamer.*

*Data for linear regioisomer iso-***6a**

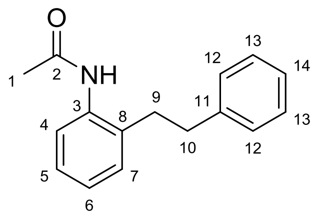

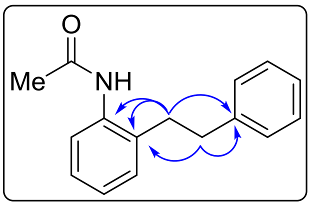

HMBC analysis

General Procedure B: [Ir(cod)$_2$]OTf and dppm were employed respectively as the pre-catalyst and the ligand. A solution of styrene (200 mol%) in anhydrous 1,4-dioxane was added to the reaction tube. The reaction was conducted for 48 hours and afforded acetanilide *iso*-**6a** (4.90 mg, 14% yield, >25:1 linear:branched) as a colorless solid; ν_{max} / cm^{-1}: 3400 (s), 3032 (m), 2974 (s), 1685 (s), 1520 (s), 1448 (s), 1386 (s), 1094 (s); ^{1}H NMR (CDCl$_3$, 400 MHz): δ 7.56 (1H, d, *J* = 8.0 Hz, C**4**-H), 7.37-7.12 (6H, m, ArC-H), 7.09 (2H, d, *J* = 8.0 Hz, 2 × C**12**-H), 6.17 (1H, br. s, N-H), 3.01-2.78 (4H, m, C**9**-H$_2$ and C**10**-H$_2$), 1.94 (3H, s, C**1**-H$_3$); ^{13}C NMR (CDCl$_3$, 100 MHz): δ 168.5 (C**2**), 141.4 (C**11**), 135.2 (C**3**), 133.8 (C**8**), 129.8 (C**7**), 128.7 (C1**3**), 128.6 (C**12**), 126.9 (C**6**), 126.4 (C**14**), 125.9 (C**5**), 124.9 (C**4**), 37.1 (C**10**), 33.8 (C**9**), 23.9 (C**1**);); HRMS: (ESI$^+$) Calculated for $C_{16}H_{18}NO$: 240.1383. Found [M+H]$^+$: 240.1382; m.p. = 102-103 ˚C (hexane/CH_2Cl_2). *The regiochemistry of compound iso-**6a** was confirmed by HMBC (as indicated above). Linear selectivity was further confirmed by ^{13}C-DEPT NMR analysis that showed 2 CH_2 signals in the alkyl region.*

***N*-(2-Methyl-6-(1-phenylethyl)phenyl)acetamide (6b)**

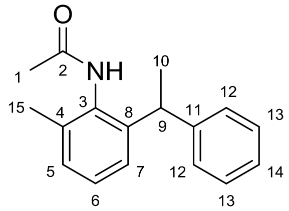

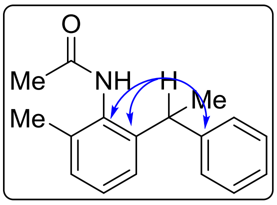

HMBC analysis

General Procedure B: [Ir(cod)$_2$]OTf was employed as the catalyst. A solution of styrene (450 mol%) in anhydrous 1,4-dioxane was added to the reaction tube. The reaction was conducted for 48 hours and afforded acetanilide **6b** (26.4 mg, 73% yield, 0.9:0.1 mixture of rotamers *A*:*B*, >25:1 branched:linear) as a colorless solid; ν_{max} / cm^{-1}: 3407 (s), 3030 (m), 2963 (s), 2940 (m), 1676 (s), 1465 (s), 1383 (s); ^{1}H NMR (CDCl$_3$, 400 MHz): δ 7.42-7.05 (8H, m, ArC-H, *A*+*B*), 6.46 (0.9H, br. s, N-H, *A*), 6.33 (0.1H, br. s, N-H, *B*), 4.33 (0.1H, q, *J* = 7.0 Hz, C**9**-H, *B*), 4.21 (0.9H, q, *J* = 7.0 Hz, C**9**-H, *A*), 2.22 (0.3H, s, C**15**-H$_3$, *B*), 2.18 (2.7H, s, C**15**-H$_3$, *A*), 2.06 (2.7H, s, C**1**-H$_3$, *A*), 1.76 (0.3H, s, C**1**-H$_3$, *B*), 1.61 (0.3H, d, *J* = 7.0 Hz, C**10**-H$_3$, *B*), 1.58 (2.7H, d, *J* = 7.0 Hz, C**10**-H$_3$, *A*); ^{13}C NMR (CDCl$_3$, 100 MHz, *major rotamer signals only*): δ 168.4 (C**2**), 146.2 (C**11**), 142.1 (C**8**), 136.7 (C**4**), 133.6 (C**3**), 129.0 (C**6**), 128.7 (C**13**), 127.6 (C**5**), 127.3 (C**12**), 126.2 (C**14**), 125.1 (C**7**), 41.0 (C**9**), 23.1 (C**1**), 21.9 (C**10**), 18.4 (C**15**); HRMS: (ESI$^+$) Calculated for $C_{17}H_{20}NO$: 254.1539. Found

[M+H]$^+$: 254.1537; m.p. = 103-104 ˚C (hexane/CH_2Cl_2). *The regiochemistry of compound* ***6b*** *was confirmed by HMBC analysis (as indicated above). Branch selectivity was further confirmed by* ^{13}*C-DEPT NMR analysis that showed 4 CH/CH_3 signals in the alkyl region.*

N-(5-(1-Phenylethyl)-2,3-dihydrobenzofuran-4-yl)acetamide (6c)

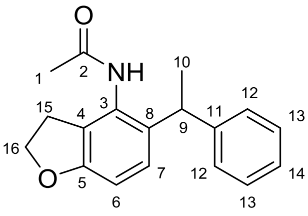

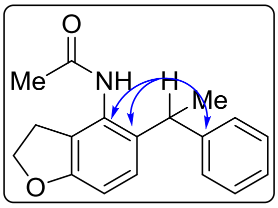

HMBC analysis

General Procedure B: [Ir(cod)$_2$]OTf was employed as the catalyst. A solution of styrene (100 mol%) in anhydrous 1,4-dioxane was added to the reaction tube. The reaction was conducted for 48 hours and afforded acetanilide **6c** (35.5 mg, 88% yield, 0.9:0.1 mixture of rotamers *A*:*B*, >25:1 branched:linear) as an off-white solid; ν_{max} / cm^{-1}: 3675 (s), 3250 (s), 2967 (m), 2901 (m), 1655 (s), 1521 (s), 1461 (s), 1236 (s), 1041 (s), 984 (s); ^{1}H NMR (CDCl$_3$, 400 MHz): δ 7.40-7.04 (6H, m, ArC-H, *A*+*B*), 6.82 (0.1H, d, *J* = 8.0 Hz, C**6**-H, *B*), 6.72 (0.9H, d, *J* = 8.0 Hz, C**6**-H, *A*), 6.53 (1H, br. s, N-H, *A*+*B*), 4.63-4.43 (2H, m, C**16**-H$_2$, *A*+*B*), 4.18 (0.1H, q, *J* = 7.0 Hz , C**9**-H, *B*), 4.11 (0.9H, q, *J* = 7.0 Hz, C**9**-H, *A*), 3.23-3.05 (1H, m, 1 × C**15**-H$_2$, *A*+*B*), 3.02-2.85 (1H, m, 1 × C**15**-H$_2$, *A*+*B*), 1.96 (2.7H, s, C**1**-H$_3$, *A*), 1.72 (0.3H, s, C**1**-H$_3$, *B*), 1.56 (3H, d, *J* = 7.0 Hz, C**10**-H$_3$, *A*+*B*); ^{13}C NMR (CDCl$_3$, 100 MHz, *major rotamer signals only*): δ 167.4 (C**2**), 159.6 (C**5**), 146.2 (C**11**), 131.6 (*2 signals*, C**3**+C**8**), 128.9 (C**13**), 127.2 (C**12**), 126.8 (C**7**), 126.4 (C**14**), 125.7 (C**4**), 107.4 (C**6**), 71.5 (C**16**), 40.6 (C**9**), 29.3 (C**15**), 23.3 (C**1**), 22.0 (C**10**); HRMS: (ESI$^+$) Calculated for $C_{18}H_{20}NO_2$: 282.1489. Found [M+H]$^+$: 282.1482; m.p. = 138-139 ˚C (hexane/CH_2Cl_2). *The regiochemistry of compound* ***6c*** *was confirmed by HMBC analysis (as indicated above). Branch selectivity was further confirmed by* ^{13}*C-DEPT NMR analysis that showed 3 CH/CH_3 signals in the alkyl region.*

N-(1-Methyl-5-(1-phenylethyl)-1*H*-indol-4-yl)acetamide (6d)

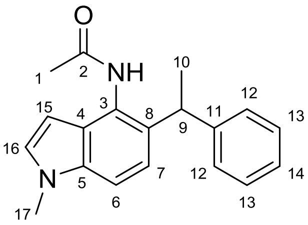

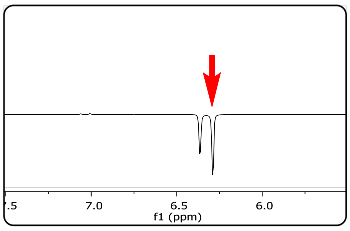

nOe analysis

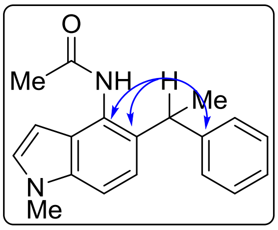

HMBC analysis

General Procedure B: [Ir(cod)$_2$]OTf was employed as the pre-catalyst. A solution of styrene (100 mol%) in anhydrous 1,4-dioxane was added to the reaction tube. The reaction was conducted for 48 hours and afforded acetanilide **6d** (34.8 mg, 83% yield, 0.6:0.4 mixture of rotamers *A*:*B*, >25:1 branched:linear) as a brown solid; ν_{max} / cm^{-1}: 3685 (s), 2987 (m), 2972 (m), 2901 (m), 1675 (s), 1406

(s), 1264 (s), 1066 (s), 893 (s); ^{1}H NMR ($CDCl_3$, 400 MHz): δ 7.40-7.09 (7H, m, ArC-H, *A*+*B*), 7.06 (0.4H, d, *J* = 3.0 Hz, C**16**-H, *B*), 7.00 (0.6H, d, *J* = 3.0 Hz, C**16**-H, *A*), 6.80 (1H, br. s, N-H, *A*+*B*), 6.36 (0.4H, d, *J* = 3.0 Hz, C**15**-H, *B*), 6.29 (0.6H, d, *J* = 3.0 Hz, C**15**-H, *A*), 4.48 (0.4H, q, *J* = 7.0 Hz , C**9**-H, *B*), 4.41 (0.9H, q, *J* = 7.0 Hz, C**9**-H, *A*), 3.79 (1.2H, s, C**17**-H_3, *B*), 3.75 (1.8H, s, C**17**-H_3, *A*), 2.14 (1.8H, s, C**1**-H_3, *A*), 1.75 (1.2H, br.s, C**1**-H_3, *B*), 1.69-1.61 (3H, m, C**10**-H_3, *A*+*B*); ^{13}C NMR ($CDCl_3$, 100 MHz): δ 168.5 (*2 signals*, C**2**, *A*+*B*), 146.7 (C**11**, *A*), 146.0 (C**11**, *B*), 136.6 (C**5**, *A*), 136.4 (C**5**, *B*), 134.0 (C**8**, *B*), 132.0 (C**8**, *A*), 130.0 (C**16**, *B*), 129.2 (C**16**, *A*), 128.6 (C**13**, *A*), 128.5 (C**13**, *B*), 127.6 (C**4**, *B*), 127.5 (C**12**, *B*), 127.4 (C**12**, *A*), 127.0 (C**3**, *B*), 126.4 (C**3**, *A*), 126.2 (C**4**, *A*), 126.0 (*2 signals*, C**14**, *A*+*B*), 121.1 (C**7**, *A*), 120.9 (C**7**, *B*), 109.4 (C**6**, *B*), 108.6 (C**6**, *A*), 99.4 (C**15**, *A*), 98.7 (C**15**, *B*), 40.1 (C**9**, *A*), 39.6 (C**9**, *B*), 33.0 (C**17**, *B*), 32.9 (C**17**, *A*), 23.5 (*2 signals*, C**1**, *A*+*B*), 22.0 (C**10**, *B*), 21.9 (C**10**, *A*); HRMS: (ESI$^+$) Calculated for $C_{19}H_{21}N_2O$: 293.1648. Found [M+H]$^+$: 293.1654; m.p. = 170-171 ˚C (hexane/CH_2Cl_2). *The regiochemistry of compound* ***6d*** *was confirmed by HMBC analysis (as indicated above). Branch selectivity was further confirmed by* ^{13}*C-DEPT NMR analysis that showed 4 CH/CH_3 signals in the alkyl region. Selective irradiation of signals for C**15**-H (as shown above) and C**16**-H of rotamer A in a 1D gradient nOe experiment revealed a negative peak for the respective signals of rotamer B.*

N-(5-(1-Phenylethyl)-2,3-dihydro-1*H*-inden-4-yl)acetamide (6e)

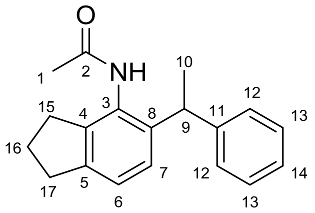

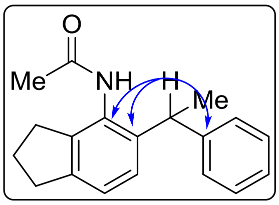

General Procedure B: [Ir(cod)$_2$]OTf was employed as the pre-catalyst. A solution of styrene (200 mol%) in anhydrous 1,4-dioxane was added to the reaction tube. The reaction was conducted for 48 hours and afforded acetanilide **6e** (40.0 mg, quantitative yield, 0.9:0.1 mixture of rotamers *A*:*B*, >25:1 branched:linear) as a colorless solid; ν_{max} / cm^{-1}: 3675 (s), 2988 (m), 2972 (s), 2901 (m), 1679 (s), 1493 (s), 1406 (s), 1242 (s), 1066 (s); ^{1}H NMR ($CDCl_3$, 400 MHz): δ 7.37-7.07 (7H, m, ArC-H, *A*+*B*), 6.50 (0.9H, br. s, N-H, *A*), 6.39 (0.1H, br. s, N-H, *B*), 4.34-4.25 (0.1H, m, C**9**-H, *B*), 4.20 (0.9H, q, *J* = 7.0 Hz, C**9**-H, *A*), 3.01-2.87 (2H, m, C**17**-H_2, *A*+*B*), 2.87-2.74 (1H, m, 1 × C**15**-H_2, *A*+*B*), 2.74-2.62 (1H, m, 1 × C**15**-H_2, *A*+*B*), 2.17-1.90 (4.7H, m, 2 × C**16**-H_2 + 3 × C**1**-H_3 *of A* and 2 × C**16**-H_2 *of B*), 1.85-1.68 (0.3H, m, C**1**-H_3, *B*), 1.58 (3H, d, *J* = 7.0 Hz, C**10**-H_3, *A*+*B*); ^{13}C NMR ($CDCl_3$, 100 MHz, *major rotamer signals only*): δ 168.0 (C**2**), 146.2 (C**11**), 143.9 (C**4**), 142.7 (C**5**), 138.7 (C**8**), 130.9 (C**3**), 128.7 (C**13**), 127.3 (C**12**), 126.2 (C**14**), 125.5 (C**7**), 123.2 (C**6**), 40.6 (C**9**), 33.0 (C**17**), 31.5 (C**15**), 25.1 (C**16**), 23.2 (C**1**), 21.9 (C**10**); HRMS: (ESI$^+$) Calculated for $C_{19}H_{22}NO$: 280.1696. Found [M+H]$^+$: 280.1704; m.p. = 120-121 ˚C (hexane/CH_2Cl_2). *The regiochemistry of compound* ***6e*** *was*

confirmed by HMBC analysis (as indicated above). Branch selectivity was further confirmed by ^{13}C-DEPT NMR analysis that showed 3 CH/CH_3 signals in the alkyl region.

N-(5-Methyl-2-(1-phenylethyl)phenyl)acetamide (6f)

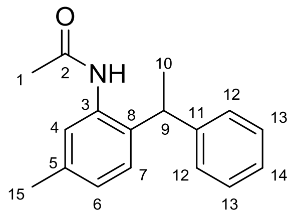

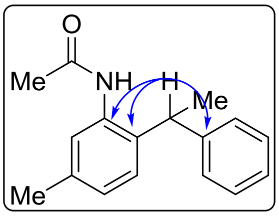

HMBC analysis

General Procedure B: [Ir(cod)$_2$]OTf was employed as the pre-catalyst. A solution of styrene (200 mol%) in anhydrous 1,4-dioxane was added to the reaction tube. The reaction was conducted for 24 hours and afforded acetanilide **6f** (36.3 mg, quantitative yield, 0.9:0.1 mixture of rotamers *A*:*B*, >25:1 branched:linear) as an off-white solid; ν_{max} / cm^{-1}: 3410 (s), 3283 (m), 3147 (s), 2978 (m), 2901 (m), 1683 (s), 1525 (s), 1471 (s), 1294 (s); ^{1}H NMR ($CDCl_3$, 400 MHz): δ 7.52 (0.9H, s, C**4**-H, *A*), 7.42-7.10 (6.2H, m, ArC-H, *A*+*B*), 7.04 (1H, d, *J* = 8.0 Hz, C**6**-H, *A*), 6.73 (1H, br. s, N-H, *A*+*B*), 4.30-4.20 (0.1H, m, C**9**-H, *B*), 4.13 (0.9H, q, *J* = 7.0 Hz, C**9**-H), 2.34 (3H, s, C**15**-H_3, *A*+*B*), 1.94 (2.7H, s, C**1**-H_3, *A*), 1.75 (0.3H, s, C**1**-H_3, *B*), 1.60 (3H, d, *J* = 7.0 Hz, C**10**-H_3, *A*+*B*); ^{13}C NMR ($CDCl_3$, 100 MHz, *major rotamer signals only*): δ 168.2 (C**2**), 145.5 (C**11**), 137.0 (C**5**), 135.0 (C**3**), 133.8 (C**8**), 129.0 (C**13**), 127.2 (C**12**), 127.1 (C**7**), 126.7 (C**14**), 126.3 (C**6**), 125.5 (C**4**), 40.5 (C**9**), 24.0 (C**1**), 21.7 (C**10**), 21.1 (C**15**); HRMS: (ESI$^+$) Calculated for $C_{17}H_{20}NO$: 254.1539. Found $[M+H]^+$: 254.1531, m.p. = 117-119 ˚C (hexane/CH_2Cl_2). *The ortho-regiochemistry of compound* ***6f*** *was confirmed by HMBC analysis (as indicated above) and by the multiplicity of C4-H signal in ^{1}H NMR (singlet). Branch selectivity was further confirmed by ^{13}C-DEPT NMR analysis that showed 4 CH/CH_3 signals in the alkyl region.*

N-(5-Methoxy-2-(1-phenylethyl)phenyl)acetamide (6g) and *iso*-6g isomer

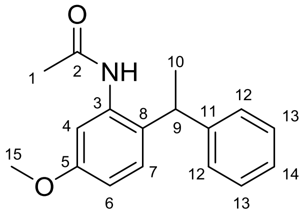

6g

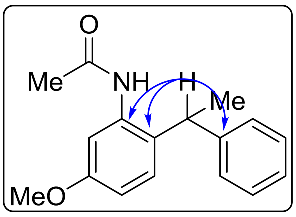

HMBC analysis

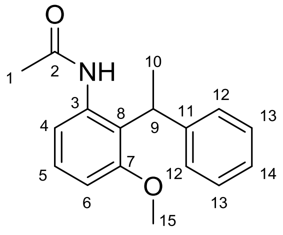

***iso*-6g**

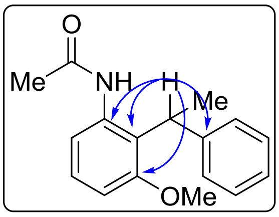

HMBC analysis

General Procedure B: [Ir(cod)$_2$]OTf was employed as the pre-catalyst. A solution of styrene (100 mol%) in anhydrous 1,4-dioxane was added to the reaction tube. The reaction was conducted for 48 hours. Purification by FCC afforded acetanilide *iso*-**6g** (5.00 mg, 13% yield, >25:1 branched:linear) as

an amber wax. Continued elution provided regioisomer **6g** (31.6 mg, 82% yield, 0.9:0.1 mixture of rotamers *A*:*B*, >25:1 branched:linear) as a colorless solid.

Data for **6g**: ν_{max} / cm^{-1}: 3398 (s), 2981 (m), 2931 (m), 1681 (s), 1474 (s), 1380 (s), 1263 (s), 1096 (s); ^{1}H NMR ($CDCl_3$, 400 MHz): δ 7.43 (0.9H, s, C**4**-H, *A*), 7.40-7.03 (6.1H, m, ArC-H, *A*+*B*), 6.87-6.62 (2H, m, C**6**-H and N-H, *A*+*B*), 4.28-4.13 (0.1H, m, C**9**-H, *B*), 4.09 (0.9H, q, *J* = 7.0 Hz, C**9**-H, *A*), 3.79 (3H, s, C**15**-H_3, *A*+*B*), 1.92 (2.7H, s, C**1**-H_3, *A*), 1.70 (0.3H, br. s, C**1**-H_3, *B*), 1.60 (3H, d, *J* = 7.0 Hz, C**10**-H_3, *A*+*B*); ^{13}C NMR ($CDCl_3$, 100 MHz, *major rotamer signals only*): δ 168.1 (C**2**), 158.6 (C**5**), 145.6 (C**11**), 136.2 (C**3**), 129.1 (C**13**), 127.9 (*2 signals*, C**7** and C**8**), 127.1 (C**12**), 126.8 (C**14**), 111.1 (C**6**), 109.5 (C**4**), 55.3 (C**15**), 40.3 (C**9**), 24.2 (C**1**), 21.9 (C**10**); HRMS: (ESI^+) Calculated for $C_{17}H_{20}NO_2$: 270.1489. Found $[M+H]^+$: 270.1480, m.p. = 125-126 ˚C (hexane/CH_2Cl_2). *The ortho-regiochemistry of compound* ***6g*** *was confirmed by HMBC analysis (as indicated above) and by the multiplicity of C4-H signal in* ^{1}*H NMR (singlet).*

Data for iso-**6g**: ν_{max} / cm^{-1}: 3675 (s), 2987 (m), 2901 (m), 1691 (s), 1450 (s), 1382 (s), 1264 (s), 1066 (s); ^{1}H NMR ($CDCl_3$, 400 MHz): δ 7.48-7.09 (7H, m, ArC-H), 6.76 (1H, d, *J* = 8.0 Hz, C**6**-H), 6.57 (1H, br. s, N-H), 5.03 (1H, q, *J* = 7.0 Hz, C**9**-H), 3.85 (3H, s, C**15**-H_3), 1.75 (3H, s, C**1**-H_3), 1.58 (3H, d, *J* = 7.0 Hz, C**10**-H_3); ^{13}C NMR ($CDCl_3$, 100 MHz): δ 167.9 (C**2**), 157.3 (C**7**), 144.2 (C**11**), 136.3 (C**3**), 128.9 (C**13**), 127.5 (C**5**), 126.8 (C**12**), 126.4 (C**14**), 125.9 (C**8**), 117.4 (C**4**), 107.7 (C**6**), 56.0 (C**15**), 32.7 (C**9**), 24.0 (C**1**), 16.7 (C**10**); HRMS: (ESI^+) Calculated for $C_{17}H_{20}NO_2$: 270.1489. Found $[M+H]^+$: 270.1477. *The ortho-regiochemistry of compound iso-**6g** was confirmed by HMBC analysis (as indicated above).*

N-(3-(1-Phenylethyl)naphthalen-2-yl)acetamide (6h) ***and iso*****-6h isomer**

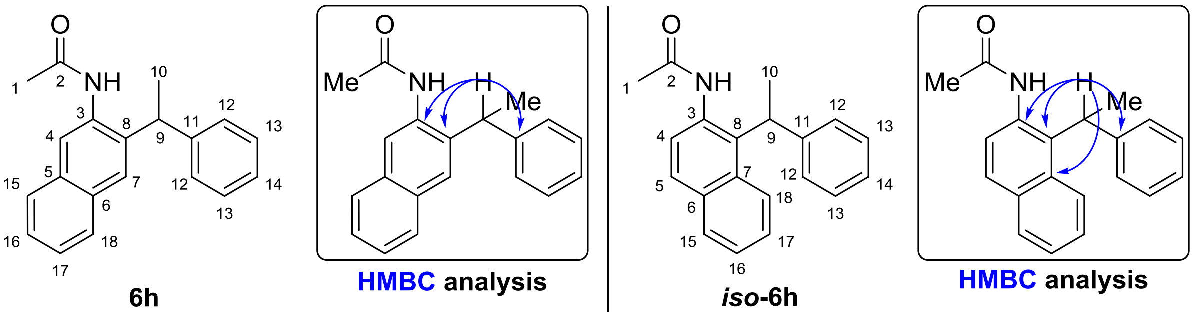

6h | **HMBC analysis** | ***iso*-6h** | **HMBC analysis**

<u>General Procedure B:</u> [Ir(cod)$_2$]OTf was employed as the pre-catalyst. A solution of styrene (200 mol%) in anhydrous 1,4-dioxane was added to the reaction tube. The reaction was conducted for 24 hours. Purification by FCC afforded acetanilide **6h** (36.8 mg, 81% yield, 0.9:0.1 mixture of rotamers *A*:*B*, >25:1 branched:linear) as an off-white solid. Continued elution provided regioisomer *iso*-**6h** (8.70 mg, 19% yield, >25:1 branched:linear) as a colorless solid.

Data for **6h**: ν_{max} / cm^{-1}: 3416 (s), 3160 (s), 2981 (m), 2873 (m), 1686 (s), 1526 (s), 1376 (s), 1256 (s); ^{1}H NMR ($CDCl_3$, 400 MHz): δ 8.30 (0.9H, s, C**4**-H, *A*), 8.00-7.72 (3.1H, m, C**7**-H, C**15**-H and C**18**-H *of A* + C**4**-H, C**7**-H, C**15**-H and C**18**-H *of B*), 7.60-7.40 (2H, m, C**16**-H and C**17**-H, *A*+*B*), 7.41-7.09 (5H, m, 2 × C**12**-H, 2 × C**13**-H and C**14**-H, *A*+*B*), 6.91 (1H, br. s, N-H, *A*+*B*), 4.29 (1H, q, *J* = 7.0 Hz, C**9**-H, *A*+*B*), 1.96 (2.7H, s, C**1**-H_3, *A*), 1.75 (3H, d, *J* = 7.0 Hz, C**10**-H_3, *A*+*B*), 1.69 (0.3H, br. s, C**1**-H_3, *B*); ^{13}C NMR ($CDCl_3$, 100 MHz, *major rotamer signals only*): δ 168.2 (C**2**), 145.1 (C**11**), 135.1 (C**8**), 133.4 (C**3**), 132.7 (C**5**), 131.0 (C**6**), 129.3 (C**13**), 127.5 (C**18**), 127.4 (C**15**), 127.3 (C**12**), 127.0 (C**14**), 126.3 (C**7**), 126.0 (C**16**), 125.5 (C**17**), 121.9 (C**4**), 41.3 (C**9**), 24.3 (C**1**), 22.1 (C**10**); HRMS: (ESI^+) Calculated for $C_{20}H_{20}NO$: 290.1539. Found $[M+H]^+$: 290.1530, m.p. = 151-153 ˚C (hexane/CH_2Cl_2). *The ortho-regiochemistry of compound* ***6h*** *was confirmed by* HMBC *analysis (as indicated above) and by the multiplicity of C4-H and C7-H signals in ^{1}H NMR (singlets). The structure of compound* ***6h*** *was definitively confirmed by single crystal X-ray diffraction of crystals obtained from CH_2Cl_2-hexane (Figure 1)* ***(CCDC 1413026)***.

.

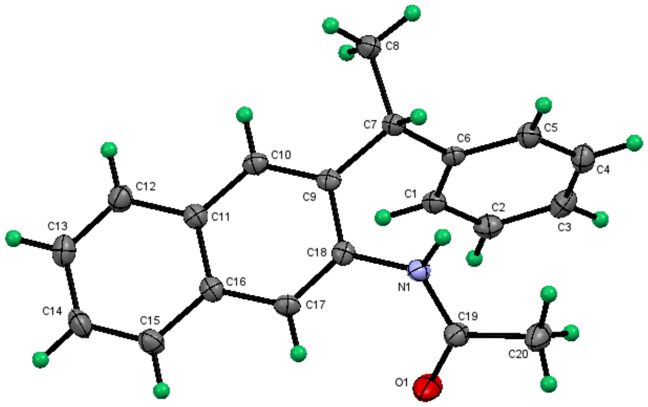

Figure 1: ORTEP view of 6h

Data for iso-**6h**: ν_{max} / cm^{-1}: 3405 (s), 2981 (m), 2891 (m), 1693 (s), 1495 (s), 1381 (s), 1161 (s); ^{1}H NMR ($CDCl_3$, 400 MHz): δ 8.17 (1H, d, *J* = 8.5 Hz, C**18**-H), 7.87 (1H, d, *J* = 8.0 Hz, C**15**-H), 7.83-7.75 (2H, 2 × d, *J* = 8.5 Hz, C**4**-H and C**5**-H), 7.58-7.42 (2H, m, C**16**-H and C**17**-H), 7.42-7.17 (5H, m, 2 × C**12**-H, 2 × C**13**-H and C**14**-H), 6.74 (1H, br. s, N-H), 5.26 (1H, q, *J* = 7.0 Hz, C**9**-H), 1.86 (3H, s, C**1**-H_3), 1.75 (3H, d, *J* = 7.0 Hz, C**10**-H_3); ^{13}C NMR ($CDCl_3$, 100 MHz): δ 168.3 (C**2**), 144.0 (C**11**), 133.1 (C**6**), 132.6 (*2 signals*, C**3** and C**8**), 132.2 (C**7**), 129.1 (C**13**), 129.0 (C**15**), 127.8 (C**5**), 126.8 (C**12**), 126.6 (*2 signals*, C**14** and C**17**), 125.1 (C**16**), 124.7 (C**4**), 123.3 (C**18**), 35.6 (C**9**), 23.8 (C**1**), 16.9 (C**10**); HRMS: (ESI^+) Calculated for $C_{20}H_{20}NO$: 290.1539. Found $[M+H]^+$: 290.1533, m.p. = 188-189 ˚C (hexane/CH_2Cl_2). Found $[M+H]^+$: 270.1477. *The ortho-regiochemistry of compound iso-**6h** was confirmed by* HMBC *analysis (as indicated above). The structure of compound iso-**6h** was definitively confirmed by single crystal X-ray diffraction of crystals obtained from CH_2Cl_2-hexane (Figure 2)* ***(CCDC 1413027)***.

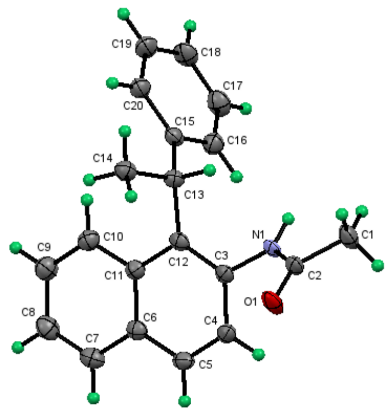

Figure 2: ORTEP view of *iso-6h*

_N_-(4-(1-Phenylethyl)benzo[*d*][1,3]dioxol-5-yl)acetamide (6i) *and iso*-6i isomer

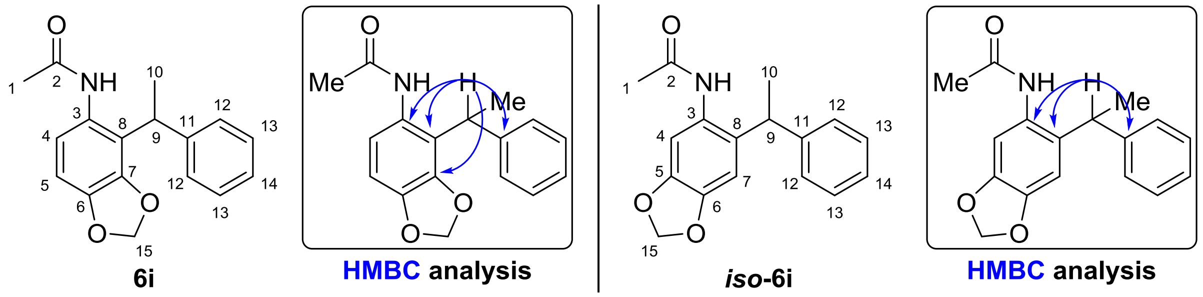

General Procedure B: The reaction was conducted using 0.28 mmol of **5i**. [Ir(cod)$_2$]OTf was employed as the pre-catalyst. A solution of styrene (100 mol%) in anhydrous 1,4-dioxane was added to the reaction tube. The reaction was conducted for 24 hours and afforded a 0.8:0.2 mixture of acetanilides **6i** and *iso*-**6i** (0.02:0.18 mixture of rotamers *A*:*B*) (67.4 mg, 83% yield, >25:1 branched:linear) as an off-white solid; ν_{max} / cm^{-1}: 3404 (s), 3054 (m), 2978 (m), 2880 (m), 1685 (s), 1448 (s), 1264 (s), 1047 (s); ^{1}H NMR (CDCl$_3$, 400 MHz): δ 7.39-7.08 (5H, m, ArC-H, **6i** and *iso*-**6i**), 7.03 (0.2H, s, C**4**-H, *A*+*B*, *iso*-**6i**), 6.95 (0.8H, d, *J* = 8.0 Hz, C**4**-H, **6i**), 6.90 (0.02H, s, C**7**-H, *B*, *iso*-**6i**), 6.85 (0.18H, s, C**7**-H, *A*, *iso*-**6i**), 6.78 (0.2H, br. s, N-H, *A*+*B*, *iso*-**6i**), 6.72 (0.8H, br. s, N-H, **6i**), 6.67 (0.8H, d, *J* = 8.0 Hz, C**5**-H, **6i**), 6.05-5.98 (0.4H, m, C**15**-H$_2$, *A*+*B*, *iso*-**6i**), 5.97-5.86 (1.6H, m, C**15**-H$_2$, **6i**), 4.36 (0.8H, q, *J* = 7.0 Hz, C**9**-H, **6i**), 4.20 (0.02H, q, *J* = 7.0 Hz, C**9**-H, *B*, *iso*-**6i**), 4.10 (0.18H, q, *J* = 7.0 Hz, C**9**-H, *A*, *iso*-**6i**), 1.97-1.83 (2.94H, m, C**1**-H$_3$ *for* **6i** and C**1**-H$_3$, *A*, *for iso*-**6i**), 1.73-162 (2.46H, m, C**10**-H$_3$ *for* **6i** and C**1**-H$_3$, *B*, *for iso*-**6i**), 1.54 (0.6H, d, *J* = 7.0 Hz, C**10**-H$_3$, *A*+*B*, *iso*-**6i**); ^{13}C NMR (CDCl$_3$, 100 MHz, *iso-**6i** major rotamer signals only*): δ 168.9 (C**2**, **6i**), 168.7 (C**2**, *iso*-**6i**), 146.1 (C**7**, **6i**), 146.0 (C**5**, *iso*-**6i**), 145.8 (C**6**, *iso*-**6i**), 145.5 (C**6**, **6i**), 145.4 (C**11**, *iso*-**6i**), 143.9 (C**11**, **6i**), 132.1 (C**8**, *iso*-**6i**), 128.9 (*2 signals*, C**3** *for* **6i** and C**13** *for iso*-**6i**), 128.8 (C**13**, **6i**), 128.6 (C**3**, *iso*-**6i**), 127.2 (C**12**, *iso*-**6i**), 127.1 (C**12**, **6i**), 126.6 (*2 signals*, C**14** *for* **6i** and C**14** *for iso*-**6i**), 122.6 (C**8**, **6i**), 119.8 (C**4**, **6i**), 107.3 (C**4**, *iso*-**6i**), 107.2 (C**7**, *iso*-**6i**), 106.5 (C**5**, **6i**), 101.3 (C**15**, *iso*-**6i**), 101.0 (C**15**, **6i**), 40.3 (C**9**, *iso*-**6i**), 36.7 (C**9**, **6i**), 23.7 (C**1**, *iso*-**6i**), 23.6 (C**1**, **6i**), 21.8 (C**10**, *iso*-**6i**), 18.6 (C**10**, **6i**); HRMS: (ESI$^+$) Calculated for $C_{17}H_{18}NO_3$: 284.1281. Found [M+H]$^+$: 284.1279. *The*

*ortho-regiochemistry of compounds **6i** and iso-**6i** was confirmed by HMBC analysis (as indicated above).*

N-(3-Fluoro-2-(1-phenylethyl)phenyl)acetamide (6j) *and iso*-6j isomer

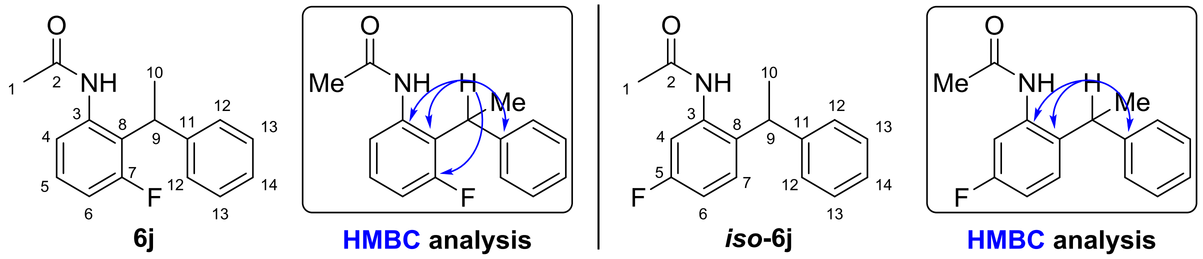

General Procedure B: $[Ir(cod)_2]OTf$ was employed as the pre-catalyst. A solution of styrene (200 mol%) in anhydrous 1,4-dioxane was added to the reaction tube. The reaction was conducted for 24 hours. Purification by FCC afforded acetanilide *iso*-**6j** (12.3 mg, 34% yield, 0.9:0.1 mixture of rotamers *A*:*B*, >25:1 branched:linear) as an amber wax. Continued elution provided regioisomer **6j** (18.8 mg, 51% yield, >25:1 branched:linear) as an amber wax.

Data for **6j**: ν_{max} / cm^{-1}: 3411 (s), 3055 (m), 2980 (m), 1696 (s), 1513 (s), 1423 (s), 1264 (s); ^{1}H NMR ($CDCl_3$, 400 MHz): δ 7.57 (1H, d, *J* = 8.0 Hz, C**4**-H), 7.42-7.24 (5H, m, ArC-H), 7.19 (1H, ddd, *J* = 8.0, 8.0 Hz, $^4J_{H-F}$ = 6.0 Hz, C**5**-H), 6.91 (1H, dd, *J* = 8.0 Hz, $^3J_{H-F}$ = 9.0 Hz, C**6**-H), 6.73 (1H, br. s, N-H), 4.73 (1H, q, *J* = 7.0 Hz, C**9**-H), 1.81 (3H, s, C**1**-H_3), 1.65 (3H, d, *J* = 7.0 Hz, C**10**-H_3); ^{13}C NMR ($CDCl_3$, 100 MHz): δ 168.1 (C**2**), 160.9 (d, $^1J_{C-F}$ = 244.0 Hz, C**7**), 142.9 (d, $^4J_{C-F}$ = 1.0 Hz, C**11**), 136.7 (d, $^3J_{C-F}$ = 5.5 Hz, C**3**), 129.1 (C**13**), 128.0 (d, $^3J_{C-F}$ = 10.0 Hz, C**5**), 126.9 (*2 signals*, C**12** and C**14**), 124.6 (d, $^2J_{C-F}$ = 15.5 Hz, C**8**), 120.4 (C**4**), 112.2 (d, $^2J_{C-F}$ = 24.0 Hz, C**6**), 33.8 (d, $^3J_{C-F}$ = 5.0 Hz, C**9**), 24.0 (C**1**), 17.5 (C**10**); HRMS: (ESI$^+$) Calculated for $C_{16}H_{17}FNO$: 258.1289. Found $[M+H]^+$: 258.1279. *The ortho-regiochemistry of compound **6j** was confirmed by the coupling constants to fluorine observed for C**8** [$^2J_{C-F}$ = 15.5 Hz] and C**9** [$^3J_{C-F}$ = 5.0 Hz]. Further confirmation was provided by HMBC analysis (as indicated above).*

Data for iso-**6j**: ν_{max} / cm^{-1}: 3404 (s), 3055 (m), 2978 (m), 2934 (m), 1695 (s), 1527 (s), 1428 (s), 1264 (s); ^{1}H NMR ($CDCl_3$, 400 MHz): δ 7.68 (0.9H, d, $^3J_{H-F}$ = 11.0 Hz, C**4**-H, *A*), 7.60-7.50 (0.1H, m, C**4**-H, *B*), 7.44-7.21 (4H, m, ArC-H, *A*+*B*), 7.16 (2H, d, *J* = 7.5 Hz, C**12**-H, *A*+*B*), 6.92 (1H, dd, *J* = 8.0 Hz, $^3J_{H-F}$ = 9.0 Hz, C**6**-H, *A*+*B*), 6.82 (0.9H, br. s, N-H, *A*), 6.70 (0.1H, br. s, N-H, *B*), 4.46 (0.1H, q, *J* = 7.0 Hz, C**9**-H, *B*), 4.09 (0.9H, q, *J* = 7.0 Hz, C**9**-H, *A*), 1.91 (2.7H, s, C**1**-H_3, *A*+*B*), 1.73-1.47 (3.3H, m, C**10**-H_3 *of A* and C**1**-H_3 and C**10**-H_3 *of B*); ^{13}C NMR ($CDCl_3$, 100 MHz, *major rotamer signals only*): δ 168.0 (C**2**), 161.6 (d, $^1J_{C-F}$ = 244.0 Hz, C**5**), 144.8 (C**11**), 136.6 (d, $^3J_{C-F}$ = 11.0 Hz, C**3**), 130.6 (d, $^4J_{C-F}$ = 1.0 Hz, C**8**), 129.3 (C**13**), 128.2 (d, $^3J_{C-F}$ = 9.0 Hz, C**7**), 127.1 (*2 signals*, C**12** and C**14**), 111.4 (d, $^2J_{C-F}$ = 21.0 Hz, C**6**), 111.0 (d, $^2J_{C-F}$ = 26.0 Hz, C**4**), 40.6 (C**9**), 24.2 (C**1**), 21.9 (C**10**);

HRMS: (ESI^+) Calculated for $C_{16}H_{17}FNO$: 258.1289. Found $[M+H]^+$: 258.1285. *The ortho-regiochemistry of compound iso-**6j** was confirmed by the coupling constants to fluorine observed for C**8** [$^4J_{C\text{-}F}$ = 1.0 Hz]. No coupling constant was observed for C**9**. Further confirmation was provided by HMBC analysis (as indicated above).*

N-(4-Methyl-2-(1-phenylethyl)phenyl)acetamide (6k)

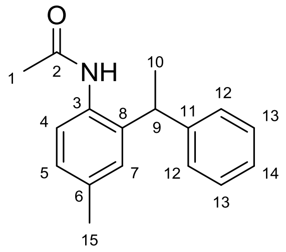

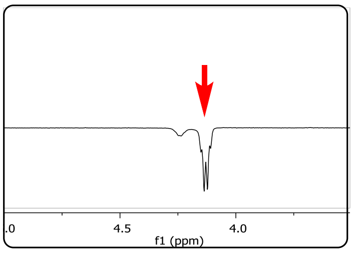

nOe analysis

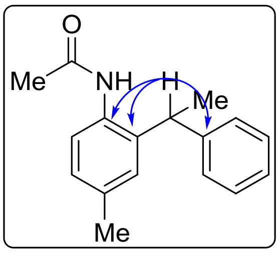

HMBC analysis

General Procedure B: [Ir(cod)$_2$]OTf was employed as the pre-catalyst. A solution of styrene (200 mol%) in anhydrous 1,4-dioxane was added to the reaction tube. The reaction was conducted for 24 hours and afforded acetanilide **6k** (30.9 mg, 85% yield, 0.9:0.1 mixture of rotamers *A*:*B*, >25:1 branched:linear) as an off-white solid; ν_{max} / cm^{-1}: 3406 (s), 3283 (m), 3143 (s), 2971 (m), 1682 (s), 1516 (s), 1373 (s); ^{1}H NMR ($CDCl_3$, 400 MHz): δ 7.50 (0.9H, d, *J* = 8.0 Hz, C**4**-H, *A*), 7.39-7.12 (6.1H, m, ArC-H, *A*+*B*), 7.07 (0.9H, d, *J* =8.0 Hz, C**5**-H, *A*); 6.98 (0.1H, d, *J* =8.0 Hz, C**5**-H, *B*), 6.67 (1H, br. s, N-H, *A*+*B*), 4.30-4.21 (0.1H, m, C**9**-H, *B*), 4.15 (0.9H, q, *J* = 7.0 Hz, C**9**-H, *A*), 2.40 (0.3H, s, C**15**-H_3, *B*), 2.37 (2.7H, s, C**15**-H_3, *A*), 1.94 (2.7H, s, C**1**-H_3, *A*), 1.75 (0.3H, s, C**1**-H_3, *B*), 1.61 (3H, d, *J* = 7.0 Hz, C**10**-H_3, *A*+*B*); ^{13}C NMR ($CDCl_3$, 100 MHz, *major rotamer signals only*): δ 168.3 (C**2**), 145.5 (C**11**), 137.1 (C**8**), 135.3 (C**6**), 132.5 (C**3**), 129.0 (C**13**), 128.0 (C**7**), 127.7 (C**5**), 127.2 (C**12**), 126.6 (C**14**), 125.3 (C**4**), 40.7 (C**9**), 23.9 (C**1**), 21.6 (C**10**), 21.3 (C**15**); HRMS: (ESI^+) Calculated for $C_{17}H_{20}NO$: 254.1539. Found $[M+H]^+$: 254.1532, m.p. = 140-141 ˚C (hexane/CH_2Cl_2). *The regiochemistry of compound **6k** was confirmed by HMBC analysis (as indicated above). Selective irradiation of signals for C**9**-H (as shown above) and C**1**-H_3 of rotamer A in a 1D gradient nOe experiment revealed a negative peak for the respective signals of rotamer B.*

N-(2-(1-Phenylethyl)-4-(trifluoromethyl)phenyl)acetamide (6l)

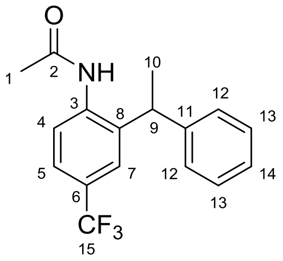

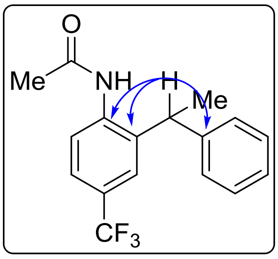

HMBC analysis

General Procedure B: [Ir(cod)$_2$]OTf was employed as the pre-catalyst. A solution of styrene (450 mol%) in anhydrous 1,4-dioxane was added to the reaction tube. The reaction was conducted for 48 hours and afforded acetanilide **6l** (9.30 mg, 21% yield, >25:1 branched:linear) as an off-white solid; ν_{max} / cm^{-1}: 3410 (s), 3254 (m), 2987 (m), 2889 (m), 1698 (s), 1595 (s), 1331 (s), 1264 (s), 1125 (s); ^{1}H NMR (CDCl$_3$, 400 MHz): δ 8.03 (1H, d, *J* = 8.5 Hz, C**4**-H), 7.68 (1H, d, *J* = 2.0 Hz, C**7**-H), 7.54 (1H, dd, *J* = 2.0, 8.0 Hz, C**5**-H); 7.35 (2H, dd, *J* = 7.0, 7.5 Hz, C**13**-H), 7.27 (1H, d, *J* = 7.0 Hz, C**14**-H), 7.16 (2H, d, *J* = 7.5 Hz, C**12**-H), 6.88 (1H, br. s, N-H), 4.15 (1H, q, *J* = 7.0 Hz, C**9**-H), 1.92 (3H, s, C**1**-H$_3$), 1.66 (3H, d, *J* = 7.0 Hz, C**10**-H$_3$); ^{13}C NMR (CDCl$_3$, 100 MHz): δ 168.0 (C**2**), 144.0 (C**11**), 138.6 (C**3**), 135.2 (C**8**), 129.5 (C**13**), 127.3 (C**14**), 127.1 (C**12**), 126.7 (q, $^2J_{C\text{-}F}$ = 31.0 Hz, C**6**), 124.5 (q, $^3J_{C\text{-}F}$ = 3.5 Hz, C**5**), 124.2 (q, $^3J_{C\text{-}F}$ = 3.5 Hz, C**7**), 124.2 (q, $^1J_{C\text{-}F}$ = 272.0 Hz, C**15**), 123.5 (C**4**), 41.0 (C**9**), 24.2 (C**1**), 21.6 (C**10**); HRMS: (ESI$^+$) Calculated for $C_{17}H_{17}F_3NO$: 308.1257. Found [M+H]$^+$: 308.1262, m.p. = 103-104 °C (hexane/CH_2Cl_2). *The regiochemistry of compound **6l** was confirmed by HMBC analysis (as indicated above).*

***N*-(4-Bromo-2-(1-phenylethyl)phenyl)acetamide (6m)**

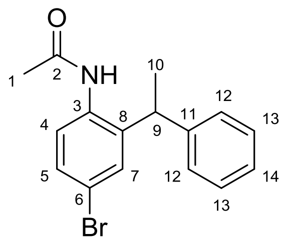

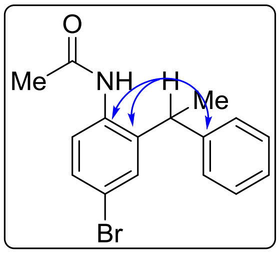

HMBC analysis

General Procedure B: [Ir(cod)$_2$]OTf was employed as the pre-catalyst. A solution of styrene (450 mol%) in anhydrous 1,4-dioxane was added to the reaction tube. The reaction was conducted for 48 hours and afforded acetanilide **6m** (27.2 mg, 60% yield, 0.9:0.1 mixture of rotamers *A*:*B*, >25:1 branched:linear) as an amber wax; ν_{max} / cm^{-1}: 3675 (s), 2987 (m), 2972 (s), 2901 (m), 1689 (s), 1494 (s), 1394 (s), 1229 (s), 1066 (s); ^{1}H NMR (CDCl$_3$, 400 MHz): δ 7.63 (0.9H, d, *J* = 8.5 Hz, C**4**-H, *A*), 7.53 (1H, s, C**7**-H, *A*+*B*), 7.46-7.20 (4.1H, m, ArC-H, *A*+*B*), 7.15 (2H, d, *J* =7.5 Hz, 2 × C**12**-H, *A*+*B*); 6.67 (1H, br. s, N-H, *A*+*B*), 4.30-4.16 (0.1H, m, C**9**-H, *B*), 4.10 (0.9H, q, *J* = 7.0 Hz, C**9**-H, *A*), 1.92 (2.7H, s, C**1**-H$_3$, *A*), 1.60 (3.3H, d, *J* = 7.0 Hz, 3 × C**10**-H$_3$ *for A* + 3 × C**1**-H$_3$ and 3 × C**10**-H$_3$ *for B*); ^{13}C NMR (CDCl$_3$, 100 MHz, *major rotamer signals only*): δ 168.1 (C**2**), 144.3 (C**11**), 138.3 (C**8**), 134.4 (C**3**), 130.3 (C**7**), 130.2 (C**5**), 129.3 (C**13**), 127.1 (*2 signals*, C**12** and C**14**), 126.1 (C**4**), 118.6 (C**6**), 40.8 (C**9**), 24.1 (C**1**), 21.6 (C**10**); HRMS: (ESI$^+$) Calculated for $C_{16}H_{17}(^{79}Br)NO$: 318.0488. Found [M+H]$^+$: 318.0491. *The regiochemistry of compound **6m** was confirmed by HMBC analysis (as indicated above).*

_N_-(2-(1-(4-Fluorophenyl)ethyl)-5-methylphenyl)acetamide (7a)

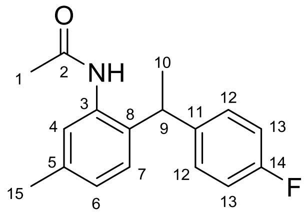

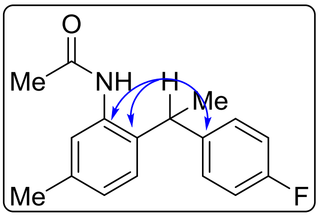

HMBC analysis

General Procedure B: [Ir(cod)$_2$]OTf was employed as the pre-catalyst. A solution of 4-fluorostyrene (200 mol%) in anhydrous 1,4-dioxane was added to the reaction tube. The reaction was conducted for 48 hours and afforded acetanilide **7a** (39.2 mg, quantitative yield, 0.9:0.1 mixture of rotamers *A*:*B*, >25:1 branched:linear) as a colorless solid; ν_{max} / cm^{-1}: 3684 (s), 2987 (m), 2972 (m), 2901 (m), 1690 (s), 1507 (s), 1393 (s), 1229 (s), 1066 (s); ^{1}H NMR (CDCl$_3$, 400 MHz): δ 7.46 (0.9H, s, C**4**-H, *A*), 7.31 (0.1H, s, C**4**-H, *B*), 7.25 (1H, d, *J* = 8.0 Hz, C**7**-H, *A*+*B*), 7.21-6.87 (5H, m, C**6**-H, 2 × C**12**-H, 2 × C**13**-H, *A*+*B*), 6.73 (1H, br. s, N-H, *A*+*B*), 4.36-4.18 (0.1H, m, C**9**-H, *B*), 4.14 (0.9H, q, *J* = 7.0 Hz, C**9**-H, *A*), 2.33 (2.7H, s, C**15**-H$_3$, *A*), 2.18 (0.3H, s, C**15**-H$_3$, *B*), 1.97 (2.7H, s, C**1**-H$_3$, *A*), 1.76 (0.3H, s, C**1**-H$_3$, *B*), 1.57 (3H, d, *J* = 7.0 Hz, C**10**-H$_3$, *A*+*B*); ^{13}C NMR (CDCl$_3$, 100 MHz, *major rotamer signals only*): δ 168.3 (C**2**), 161.4 (d, $^1J_{C\text{-}F}$ = 245.0 Hz, C**14**), 141.3 (d, $^4J_{C\text{-}F}$ = 3.0 Hz, C**11**), 137.1 (C**5**), 134.8 (C**3**), 134.1 (C**8**), 128.7 (d, $^3J_{C\text{-}F}$ = 8.0 Hz, C**12**), 127.1 (C**7**), 126.6 (C**6**), 125.9 (C**4**), 115.7 (d, $^2J_{C\text{-}F}$ = 21.0 Hz, C**13**), 39.5 (C**9**), 24.0 (C**1**), 21.9 (C**10**), 21.0 (C**15**); HRMS: (ESI$^+$) Calculated for $C_{17}H_{19}FNO$: 272.1445, Found [M+H]$^+$: 272.1444; m.p. = 119-120 ˚C (hexane/CH_2Cl_2). *The regiochemistry of compound **7a** was confirmed by HMBC analysis (as indicated above).*

_N_-(2-(1-(3-Chlorophenyl)ethyl)-5-methylphenyl)acetamide (7b)

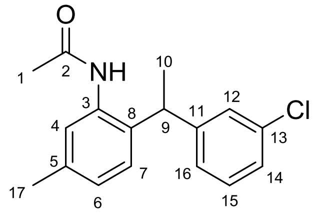

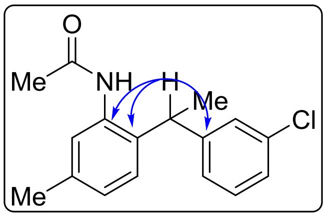

HMBC analysis

General Procedure B: [Ir(cod)$_2$]OTf was employed as the pre-catalyst. A solution of 3-chlorostyrene (200 mol%) in anhydrous 1,4-dioxane was added to the reaction tube. The reaction was conducted for 48 hours and afforded acetanilide **7b** (39.8 mg, 97% yield, 0.9:0.1 mixture of rotamers *A*:*B*, >25:1 branched:linear) as an off-white solid; ν_{max} / cm^{-1}: 3675 (s), 3416 (m), 3238 (m), 2972 (m), 2901 (m), 1673 (s), 1460 (s), 1407 (s), 1264 (s), 1075 (s), 893 (s); ^{1}H NMR (CDCl$_3$, 400 MHz): δ 7.41 (0.9H, s, C**4**-H, *A*), 7.35-7.10 (4.1H, m, C**7**-H, C**12**-H, C**14**-H and C**15**-H *of A* + C**4**-H, C**7**-H, C**12**-H, C**14**-H and C**15**-H *of B*), 7.10-6.99 (2H, m, C**6**-H and C**16**-H, *A*+*B*), 6.94 (0.1H, br. s, N-H, *B*), 6.75 (0.9H, br. s, N-H, *A*), 4.31-4.19 (0.1H, m, C**9**-H, *B*), 4.13 (0.9H, q, *J* = 7.0 Hz, C**9**-H, *A*), 2.33 (3H, s, C**17**-H$_3$, *A*+*B*), 1.99 (2.7H, s, C**1**-H$_3$, *A*), 1.75 (0.3H, s, C**1**-H$_3$, *B*), 1.57 (3H, d, *J* = 7.0 Hz, C**10**-H$_3$, *A*+*B*);

^{13}C NMR ($CDCl_3$, 100 MHz, *major rotamer signals only*): δ 168.4 (C**2**), 147.8 (C**11**), 137.3 (C**5**), 134.7 (*2 signals*, C**3** and C**13**), 134.0 (C**8**), 130.2 (C**15**), 127.4 (C**12**), 127.2 (C**7**), 126.9 (C**14**), 126.8 (C**6**), 126.3 (C**4**), 125.5 (C**16**), 39.9 (C**9**), 23.9 (C**1**), 21.6 (C**10**), 21.0 (C**17**); HRMS: (ESI^+) Calculated for $C_{17}H_{19}(^{35}Cl)NO$: 288.1150. Found $[M+H]^+$: 288.1143; m.p. = 81-82 ˚C (hexane/CH_2Cl_2). *The regiochemistry of compound **7b** was confirmed by HMBC analysis (as indicated above).*

N-(5-Methyl-2-(1-(p-tolyl)ethyl)phenyl)acetamide (7c)

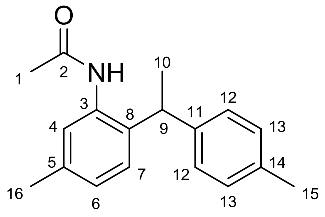

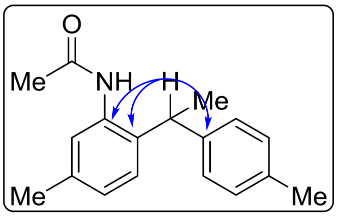

HMBC analysis

General Procedure B: [Ir(cod)$_2$]OTf was employed as the pre-catalyst. A solution of 4-methylstyrene (200 mol%) in anhydrous 1,4-dioxane was added to the reaction tube. The reaction was conducted for 48 hours and afforded acetanilide **7c** (31.0 mg, 81% yield, 0.9:0.1 mixture of rotamers *A*:*B*, >25:1 branched:linear) as a yellow oil; ν_{max} / cm^{-1}: 3675 (s), 3058 (m), 2986 (m), 2901 (m), 1688 (s), 1512 (s), 1421 (s), 1264 (s), 1047 (s); ^{1}H NMR ($CDCl_3$, 400 MHz): δ 7.54 (0.9H, s, C**4**-H, *A*), 7.40-7.27 (1.1H, s + d, J = 8.0 Hz, C**7**-H *of A* + C**4**-H and C**7**-H *of B*), 7.21-6.92 (5H, m, C**6**-H, 2 × C**12**-H, 2 × C**13**-H, *A*+*B*), 6.72 (0.9H, br. s, N-H, *A*), 6.63 (0.1H, s, N-H, *B*), 4.24-4.14 (0.1H, m, C**9**-H, *B*), 4.08 (0.9H, q, J = 7.0 Hz, C**9**-H, *A*), 2.34 (3H, s, C**16**-H_3, *A*+*B*), 2.32 (3H, s, C**15**-H_3, *A*+*B*), 1.95 (2.7H, s, C**1**-H_3, *A*), 1.70 (0.3H, s, C**1**-H_3, *B*), 1.58 (3H, d, J = 7.0 Hz, C**10**-H_3, *A*+*B*); ^{13}C NMR ($CDCl_3$, 100 MHz, *major rotamer signals only*): δ 168.1 (C**2**), 142.4 (C**11**), 136.9 (C**5**), 136.3 (C**14**), 135.0 (C**3**), 133.6 (C**8**), 129.7 (C**13**), 127.1 (*2 signals*, C**7** and C**12**), 126.2 (C**6**), 125.3 (C**4**), 40.2 (C**9**), 24.1 (C**1**), 21.8 (C**10**), 21.1 (C**16**), 21.0 (C**15**); HRMS: (ESI^+) Calculated for $C_{18}H_{22}NO$: 268.1696, Found $[M+H]^+$: 268.1689. *The regiochemistry of compound **7c** was confirmed by HMBC analysis (as indicated above).*

N-(2-(1-(2-Fluorophenyl)ethyl)-5-methylphenyl)acetamide (7d)

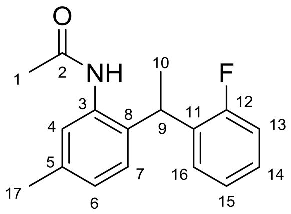

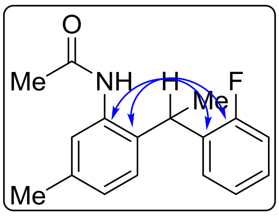

HMBC analysis

General Procedure B: [Ir(cod)$_2$]OTf was employed as the pre-catalyst. A solution of 2-fluorostyrene (200 mol%) in anhydrous 1,4-dioxane was added to the reaction tube. The reaction was conducted for 48 hours and afforded acetanilide **7d** (34.8 mg, 90% yield, 0.9:0.1 mixture of rotamers *A*:*B*, >25:1

branched:linear) as an off-white solid; ν_{max} / cm^{-1}: 3675 (s), 3418 (m), 2987 (m), 2972 (m), 2901 (m), 1691 (s), 1579 (s), 1393 (s), 1241 (s), 1056 (s); ^1H NMR ($CDCl_3$, 400 MHz): δ 7.49 (1H, s, C**4**-H, *A*+*B*), 7.31 (1H, d, *J* = 8.0 Hz, C**7**-H, *A*+*B*), 7.18 (1H, dddd, *J* = 3.5, 8.0, 9.0 Hz, $^4J_{H\text{-}F}$ = 4.0 Hz, C**14**-H, *A*+*B*), 7.13-6.97 (4H, m, C**6**-H, C**13**-H, C**15**-H and C**16**-H, *A*+*B*), 6.89 (1H, br. s, N-H, *A*+*B*), 4.64-4.52 (0.1H, m, C**9**-H, *B*), 4.49 (0.9H, q, *J* = 7.0 Hz, C**9**-H, *A*), 2.32 (3H, s, C**17**-H_3, *A*+*B*), 2.05 (2.7H, s, C**1**-H_3, *A*), 1.73 (0.3H, s, C**1**-H_3, *B*), 1.60 (3H, d, *J* = 7.0 Hz, C**10**-H_3, *A*+*B*); ^{13}C NMR ($CDCl_3$, 100 MHz, *major rotamer signals only*): δ 168.5 (C**2**), 159.9 (d, $^1J_{C\text{-}F}$ = 243.5 Hz, C**12**), 137.0 (C**5**), 134.6 (C**3**), 133.4 (C**8**), 132.2 (d, $^2J_{C\text{-}F}$ = 14.0 Hz, C**11**), 128.5 (d, $^3J_{C\text{-}F}$ = 4.0 Hz, C**16**), 128.1 (d, $^3J_{C\text{-}F}$ = 8.5 Hz, C**14**), 126.7 (C**6**), 126.6 (C**7**), 125.8 (C**4**), 124.8 (d, $^4J_{C\text{-}F}$ = 3.0 Hz, C**15**), 115.3 (d, $^2J_{C\text{-}F}$ = 23.0 Hz, C**13**), 31.7 (d, $^3J_{C\text{-}F}$ = 3.0 Hz, C**9**), 24.0 (C**1**), 21.1 (C**17**), 20.5 (C**10**); HRMS: (ESI^+) Calculated for $C_{17}H_{19}FNO$: 272.1445, Found $[M+H]^+$: 272.1443; m.p. = 122-123 ˚C (hexane/CH_2Cl_2). *The regiochemistry of compound **7d** was confirmed by HMBC analysis (as indicated above).*

***N*-(2-(1-(2-Chlorophenyl)ethyl)-5-methylphenyl)acetamide (7e)**

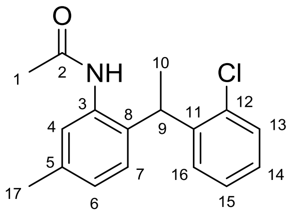

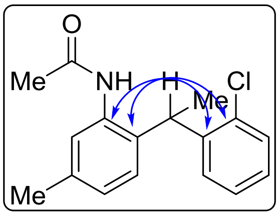

HMBC analysis

General Procedure B: $[Ir(cod)_2]OTf$ was employed as the pre-catalyst. A solution of 2-chlorostyrene (450 mol%) in anhydrous 1,4-dioxane was added to the reaction tube. The reaction was conducted for 48 hours and afforded acetanilide **7e** (18.2 mg, 44% yield, 0.9:0.1 mixture of rotamers *A*:*B*, >25:1 branched:linear) as an amber wax; ν_{max} / cm^{-1}: 3675 (s), 3420 (m), 3225 (s), 2987 (m), 2901 (m), 1691 (s), 1394 (s), 1264 (s), 1066 (s), 893 (s); ^1H NMR ($CDCl_3$, 400 MHz): δ 7.56 (1H, s, C**4**-H, *A*+*B*), 7.38 (1H, ddd, *J* = 3.5, 7.0, 7.0 Hz, C**15**-H, *A*+*B*), 7.33 (1H, d, *J* = 8.0 Hz, C**7**-H, *A*+*B*), 7.18-7.10 (2H, m, C**13**-H and C**14**-H, *A*+*B*), 7.04 (1H, d, *J* = 8.0 Hz, C**6**-H, *A*+*B*), 6.95 (1H, dd, *J* = 3.5, 7.0 Hz, C**16**-H, *A*+*B*), 6.66 (1H, br. s, N-H, *A*+*B*), 4.74-4.64 (0.1H, m, C**9**-H, *B*), 4.61 (0.9H, q, *J* = 7.0 Hz, C**9**-H, *A*), 2.33 (3H, s, C**17**-H_3, *A*+*B*), 2.02 (2.7H, s, C**1**-H_3, *A*), 1.70 (0.3H, s, C**1**-H_3, *B*), 1.55 (3H, d, *J* = 7.0 Hz, C**10**-H_3, *A*+*B*); ^{13}C NMR ($CDCl_3$, 100 MHz, *major rotamer signals only*): δ 168.4 (C**2**), 142.9 (C**11**), 137.2 (C**5**), 134.9 (C**3**), 132.8 (C**8**), 132.5 (C**12**), 129.4 (C**15**), 128.9 (C**16**), 127.9 (C**14**), 127.8 (C**13**), 126.6 (C**7**), 126.2 (C**6**), 125.4 (C**4**), 36.0 (C**9**), 24.1 (C**1**), 21.1 (C**17**), 20.5 (C**10**); HRMS: (ESI^+) Calculated for $C_{17}H_{19}(^{35}Cl)NO$: 288.1150. Found $[M+H]^+$: 288.1146. *The regiochemistry of compound **7e** was confirmed by HMBC analysis (as indicated above).*

N-(2-(Hexan-2-yl)-5-methylphenyl)acetamide (7f)

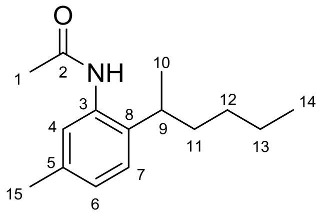

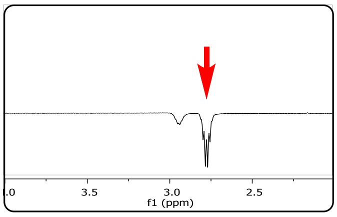

nOe analysis

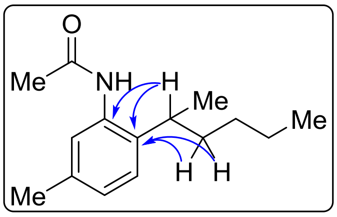
HMBC analysis

General Procedure B: [Ir(cod)$_2$]BARF was employed as the pre-catalyst. A solution of 1-hexene (600 mol%) in anhydrous 1,2-dichlorobenzene was added to the reaction tube. The reaction was conducted for 48 hours and afforded acetanilide **7f** (32.0 mg, 96% yield, 0.8:0.2 mixture of rotamers *A*:*B*, >25:1 branched:linear) as an amber wax; ν_{max} / cm^{-1}: 3675 (s), 3054 (m), 2967 (m), 2927 (m), 2886 (m), 1687 (s), 1524 (s), 1393 (s), 1264 (s), 1051 (s); ^{1}H NMR (CDCl$_3$, 400 MHz): δ 7.39 (0.8H, s, C**4**-H, *A*), 7.24-6.86 (3.2H, m, C**6**-H, C**7**-H and N-H *of A* + C**4**-H, C**6**-H, C**7**-H and N-H *of B*), 3.03-2.88 (0.2H, m, C**9**-H, *B*), 2.79 (0.8H, tq, *J* = 7.0, 7.0 Hz, C**9**-H, *A*), 2.31 (3H, s, C**15**-H$_3$, *A*+*B*), 2.17 (2.4H, s, C**1**-H$_3$, *A*), 1.88 (0.6H, s, C**1**-H$_3$, *B*), 1.64-1.44 (2H, m, C**11**-H$_2$, *A*+*B*), 1.37-1.02 (7H, m, C**10**-H$_3$, C**12**-H$_2$, C**13**-H$_2$, *A*+*B*), 0.85 (3H, t, *J* = 7.0 Hz, C**14**-H$_3$, *A*+*B*); ^{13}C NMR (CDCl$_3$, 100 MHz, *major rotamer signals only*): δ 168.7 (C**2**), 137.4 (C**8**), 135.9 (C**5**), 134.1 (C**3**), 127.2 (C**6**), 126.0 (*2 signals*, C**4** and C**7**), 37.5 (C**11**), 33.0 (C**9**), 29.9 (C**12**), 24.1 (C**1**), 22.8 (C**13**), 21.5 (C**10**), 21.0 (C**15**), 14.0 (C**14**); HRMS: (ESI$^+$) Calculated for $C_{15}H_{24}NO$: 234.1852, Found [M+H]$^+$: 234.1846. *The branch selectivity of compound **7f** was confirmed by ^{13}C-DEPT NMR analysis, which showed 5 CH/CH$_3$ signals in the aliphatic region. Ortho-regioselectivity was confirmed by HMBC analysis (as indicated above). Selective irradiation of signals for C**9**-H (as shown above) and C**1**-H$_3$ of rotamer A in a 1D gradient nOe experiment revealed a negative peak for the respective signals of rotamer B.*

N-(2-Isopropyl-5-methylphenyl)acetamide (7g)

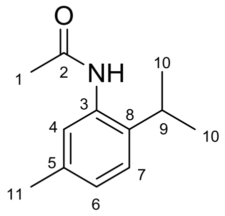

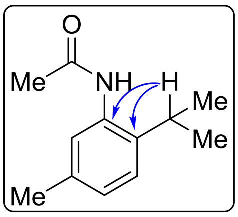
HMBC analysis

An oven-dried re-sealable tube, fitted with a magnetic stirrer, was charged with 3'-methylacetanilide **7g** (21.3 mg, 0.143 mmol), [Ir(cod)$_2$]BARF (9.1 mg, 5 mol%) and d^{F}ppb (5.6 mg, 5 mol%). The tube was fitted with a rubber septum and evacuated and backfilled with nitrogen three times. Anhydrous 1,2-dichlorobenzene (1.5 M concentration with respect to substrate) was added and the resulting solution was purged with propylene for 5 minutes. The rubber septum was removed and the vessel was sealed immediately with a Young's tap. The reaction mixture was stirred at 120 °C for 48 hours and then concentrated *in vacuo*. The crude material was purified by FCC (10% EtOAc/toluene →

30% EtOAc/toluene) to provide acetanilide **7g** (25.2 mg, 92% yield, 0.8:0.2 mixture of rotamers *A*:*B* >25:1 branched:linear) as a colorless solid; ν_{max} / cm^{-1}: 3680 (s), 3053 (m), 2966 (m), 1670 (s), 1528 (s), 1421 (s), 1264 (s), 1058 (s); ^{1}H NMR ($CDCl_3$, 400 MHz): δ 7.38 (0.8H, s, C**4**-H, *A*), 7.26-6.85 (3.2H, m, C**6**-H, C**7**-H and N-H *of A* + C**4**-H, C**6**-H, C**7**-H and N-H *of B*), 3.20-3.05 (0.2H, m, C**9**-H, *B*), 2.98 (0.8H, sept, *J* = 7.0 Hz, C**9**-H, *A*), 2.29 (3H, s, C**11**-H_3, *A*+*B*), 2.17 (2.4H, s, C**1**-H_3, *A*), 1.87 (0.6H, s, C**1**-H_3, *B*), 1.20 (6H, d, *J* = 7.0 Hz, 2 × C**10**-H_3, *A*+*B*); ^{13}C NMR ($CDCl_3$, 100 MHz, *major rotamer signals only*): δ 168.8 (C**2**), 138.2 (C**8**), 136.0 (C**5**), 133.7 (C**3**), 127.2 (C**6**), 126.0 (C**4**), 125.5 (C**7**), 27.7 (C**9**), 24.1 (C**1**), 23.2 (C**10**), 20.9 (C**11**); HRMS: (ESI^+) Calculated for $C_{12}H_{18}NO$: 192.1383, Found $[M+H]^+$: 192.1380; m.p. = 105-107 ˚C (hexane/CH_2Cl_2). *The branch selectivity of compound **7g** was confirmed by ^{13}C-DEPT NMR analysis, which showed 4 CH/CH_3 signals in the aliphatic region. Ortho-regioselectivity was confirmed by HMBC analysis (as indicated above).*

N-(5-Methyl-2-(3-methylbutan-2-yl)phenyl)acetamide (7h)

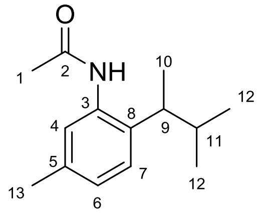

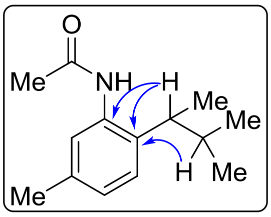

HMBC analysis

General Procedure B: [Ir(cod)$_2$]BARF was employed as the pre-catalyst. A solution of 3-methyl-1-butene (600 mol%) in anhydrous 1,2-dichlorobenzene was added to the reaction tube. The reaction was conducted for 48 hours and afforded acetanilide **7h** (10.3 mg, 33% yield, 0.8:0.2 mixture of rotamers *A*:*B*, >25:1 branched:linear) as an amber wax; ν_{max} / cm^{-1}: 3675 (s), 2987 (m), 2901 (m), 1688 (s), 1450 (s), 1393 (s), 1264 (s), 1056 (s); ^{1}H NMR ($CDCl_3$, 400 MHz): δ 7.41 (0.8H, s, C**4**-H, *A*), 7.21-7.05 (1.2H, m, C**7**-H *of A* + C**4**-H and C**7**-H *of B*), 7.04-6.80 (2H, m, C**6**-H and N-H, *A*+*B*), 2.77-2.61 (0.2H, m, C**9**-H, *B*), 2.52 (0.8H, dq, *J* = 7.0, 7.0 Hz, C**9**-H, *A*), 2.31 (3H, s, C**13**-H_3, *A*+*B*), 2.18 (2.4H, s, C**1**-H_3, *A*), 1.88 (0.6H, s, C**1**-H_3, *B*), 1.74 (1H, dqq, *J* = 6.5, 6.5, 6.5 Hz, C**11**-H, *A*+*B*), 1.19 (3H, d, *J* = 7.0 Hz, C**10**-H_3, *A*+*B*), 0.94 (3H, d, *J* = 6.5 Hz, C**12**-H_3, *A*+*B*), 0.76 (2.4H, d, *J* = 6.5 Hz, C**12**-H_3, *A*), 0.71 (0.6H, d, *J* = 6.5 Hz, C**12**-H_3, *B*); ^{13}C NMR ($CDCl_3$, 130 MHz, *major rotamer signals only*): δ 168.6 (C**2**), 136.8 (C**8**), 135.9 (C**5**), 134.2 (C**3**), 127.0 (C**6**), 126.8 (C**7**), 125.9 (C**4**), 40.0 (C**9**), 34.1 (C**11**), 24.2 (C**1**), 21.4 (C**12**), 21.0 (C**13**), 20.0 (C**12**), 18.3 (C**10**); HRMS: (ESI^+) Calculated for $C_{14}H_{22}NO$: 220.1696, Found $[M+H]^+$: 220.1700. *The branch selectivity of compound **7h** was confirmed by ^{13}C-DEPT NMR analysis, which showed 7 CH/CH_3 signals in the aliphatic region. Ortho-regioselectivity was confirmed by HMBC analysis (as indicated above).*

N-(5-Methyl-2-(4-methylpentan-2-yl)phenyl)acetamide (7i)

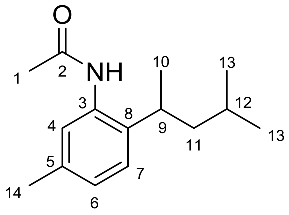

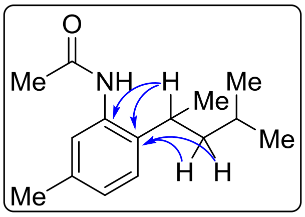

HMBC analysis

General Procedure B: [Ir(cod)$_2$]BARF (13.6 mg, 7.5 mol%) and d^{F}ppb (8.42 mg, 7.5 mol%) were employed. A solution of 4-methyl-1-pentene (600 mol%) in anhydrous 1,2-dichlorobenzene was added to the reaction tube. The reaction was conducted for 48 hours and afforded acetanilide **7i** (33.1 mg, 99% yield, 0.8:0.2 mixture of rotamers *A*:*B*, >25:1 branched:linear) as a colorless solid; ν_{max} / cm^{-1}: 3675 (s), 3054 (m), 2987 (m), 2901 (m), 1689 (s), 1469 (s), 1407 (s), 1264 (s), 1075 (s); ^{1}H NMR (CDCl$_3$, 400 MHz): δ 7.37 (0.8H, s, C**4**-H, *A*), 7.22-6.87 (3.2H, m, C**6**-H, C**7**-H and N-H *of A* + C**4**-H, C**6**-H, C**7**-H and N-H *of B*), 3.14-2.99 (0.2H, m, C**9**-H, *B*), 2.89 (0.8H, tq, J = 7.0, 7.0 Hz, C**9**-H, *A*), 2.30 (3H, s, C**14**-H$_3$, *A*+*B*), 2.16 (2.4H, s, C**1**-H$_3$, *A*), 1.88 (0.6H, s, C**1**-H$_3$, *B*), 1.57-1.27 (3H, m, 2 × C**11**-H$_2$ and C**12**-H, *A*+*B*), 1.17 (3H, d, J = 7.0 Hz, C**10**-H$_3$, *A*+*B*), 0.85 (6H, t, J = 5.5 Hz, 2 × C**13**-H$_3$, *A*+*B*); ^{13}C NMR (CDCl$_3$, 100 MHz, *major rotamer signals only*): δ 168.7 (C**2**), 137.6 (C**8**), 135.9 (C**5**), 133.9 (C**3**), 127.3 (C**6**), 126.1 (*2 signals*, C**4** and C**7**), 47.4 (C**11**), 30.5 (C**9**), 25.5 (C**12**), 24.0 (C**1**), 22.8 (C**13**), 22.7 (C**13**), 21.4 (C**10**), 20.9 (C**14**); HRMS: (ESI$^+$) Calculated for $C_{15}H_{24}NO$: 234.1852, Found [M+H]$^+$: 234.1850; m.p. = 88-89 ˚C (hexane/CH_2Cl_2). *The branch selectivity of compound **7i** was confirmed by ^{13}C-DEPT NMR analysis, which showed 7 CH/CH_3 signals in the aliphatic region. Ortho-regioselectivity was confirmed by HMBC analysis (as indicated above).*

Mechanistic Studies

General Procedure C for deuterium exchange experiments

An oven-dried re-sealable tube, fitted with a magnetic stirrer, was charged with acetanilide substrate (0.143 mmol, 100 mol%), [Ir(cod)$_2$]OTf (3.9 mg, 5 mol%) and d^{F}ppb (5.6 mg, 5 mol%). The tube was fitted with a rubber septum and purged with nitrogen. A solution of the deuterium oxide (77 µL, 4.29 mmol, 3000 mol%) in anhydrous 1,4-dioxane (1.5 M concentration with respect to substrate) was added *via* syringe and the tube was sealed with a Young's tap. The reaction vessel was placed into a pre-heated heating block at 120 °C and stirred for 48 hours. The reaction mixture was cooled to room temperature and concentrated *in vacuo*. Purification of the residue by FCC (30% EtOAc/hexane) afforded the pure product.

***Deuterio*-7c**

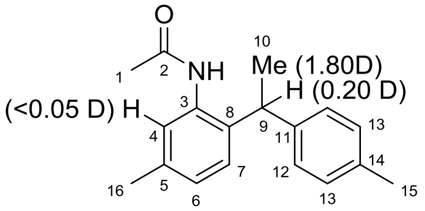

General Procedure B: [Ir(cod)$_2$]OTf was employed as the pre-catalyst. A solution of 4-methylstyrene-*β,β*-d_2 (200 mol%) in anhydrous 1,4-dioxane was added to the reaction tube. The reaction was conducted for 48 hours and afforded acetanilide *deuterio*-**7c** (29.7 mg, 77% yield, 0.9:0.1 mixture of rotamers *A*:*B*, >25:1 branched:linear) as a yellow oil; ^{1}H NMR (CDCl$_3$, 400 MHz): δ 7.53 (0.9H, s, C**4**-H, *A*), 7.40-7.22 (1.1H, s + d, *J* = 8.0 Hz, C**7**-H *of A* + C**4**-H and C**7**-H *of B*), 7.19-6.93 (5H, m, C**6**-H, 2 × C**12**-H, 2 × C**13**-H, *A*+*B*), 6.74 (0.9H, br. s, N-H, *A*), 6.64 (0.1H, s, N-H, *B*), 4.25-4.01 (0.80H, m, C**9**-H, *A*+*B*), 2.33 (3H, s, C**16**-H$_3$, *A*+*B*), 2.31 (3H, s, C**15**-H$_3$, *A*+*B*), 1.94 (2.7H, s, C**1**-H$_3$, *A*), 1.69 (0.3H, s, C**1**-H$_3$, *B*), 1.64-1.47 (1.2H, m, C**10**-H$_3$, *A*+*B*); ^{2}H NMR (CH$_2$Cl$_2$, 500 MHz): δ 4.26-3.95 (0.20D, m, C**9**-D, *A*+*B*), 1.50 (1.80D, s, C**10**-D, *A*+*B*). *Deuterium incorporation was calculated by integration of ^{1}H NMR and ^{2}H NMR signals.*

***Deuterio*-5f**

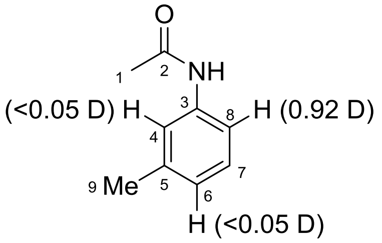

General Procedure C: The reaction afforded acetanilide *deuterio*-**5f** as a colorless solid; ^{1}H NMR (DMSO-d_6, 400 MHz): δ 9.79 (1H, br. s, N-H), 7.37 (1H, s, C**4**-H), 7.31 (0.08H, d, *J* = 8.0 Hz, C**8**-H), 7.12 (1H, d, *J* = 7.5 Hz, C**6**-H), 6.90-6.71 (1H, m, C**7**-H), 2.23 (3H, s, C**9**-H$_3$), 1.99 (3H, s, C**1**-H$_3$); ^{2}H NMR (DMSO, 500 MHz): δ 7.54-7.13 (0.92D, m, C**8**-D). *Deuterium incorporation was calculated by integration of ^{1}H NMR signals.*

Further deuterium exchange experiments are shown below:

Exposure of aniline **5f** to the Ir-catalyst system, in the absence of the alkene, but in the presence of D_2O (3000 mol%), resulted in 92% deuterium incorporation at C8-H and <5% incorporation at C4-H (see above). For acetanilides **5j** and **5g**, which give lower levels of *ortho*-selectivity (1.5:1 and 6.3:1 respectively, see Table 2 in the main paper), exchange was observed at both *ortho*-positions. However, in both cases, the *ortho*-regioselectivity of alkene hydroarylation does not reflect the observed levels of deuterium incorporation. This indicates that, in these cases, *ortho*-regioselectivity (C8 vs C4) is determined at the stage of C-C reductive elimination, rather than by *ortho*-regioselective

C-H oxidative addition. This interpretation is consistent with our earlier work on benzamide and ketone directed processes.[1a] For **5g**, deuterium incorporation was also observed at C6-H, which suggests that, for this electron rich system, the methoxy group is able to direct oxidative addition of the Ir(I)-catalyst; however, products of C-C bond formation at this position were not observed. When the exchange experiment was conducted on reaction product **6g**, deuterium incorporation was observed at C6-H, but not at the remaining *ortho*-C-H (C4-H). This reflects the recalcitrance of the initially formed adduct **6g** to undergo a second *ortho*-alkylation event. Exposure of N-methylated derivative **N-Me-5a** to the Ir(I)-system in the presence of D_2O resulted in no deuterium incorporation on the arene and preferential exchange at the N-methyl group. This is consistent with the low reactivity observed for this substrate during optimization (see Table 1, Entry 12 in the main paper). Variable levels of deuterium incorporation were observed at the acetanilide NH in all cases as a result of facile exchange with D_2O. Additionally, we have confirmed that no C-H deuteration occurs in the absence of catalyst, even for activated systems such as acetanilide **5g**.

***Deuterio*-5j**

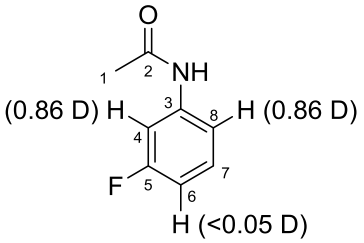

General Procedure C: The reaction afforded acetanilide *deuterio*-**5j** as a colorless solid; ^{1}H NMR (DMSO-d_6, 400 MHz): δ 10.12 (1H, br. s, N-H), 7.57 (0.14H, d, $^3J_{H-F}$ = 12.0 Hz, C**4**-H), 7.35-7-27 (1H, m, C**7**-H), 7.25 (0.14H, d, J = 8.0 Hz, C**8**-H), 6.84 (1H, dd, J = 8.5 Hz, $^3J_{H-F}$ = 9.0 Hz, C**6**-H), 2.05 (3H, s, C**1**-H_3); ^{2}H NMR (DMSO, 500 MHz): δ 7.52 (0.86D, s, C**4**-D), 7.20 (0.86D, s, C**8**-D). *Deuterium incorporation was calculated by integration of ^{1}H NMR and ^{2}H NMR signals.*

***Deuterio*-5g**

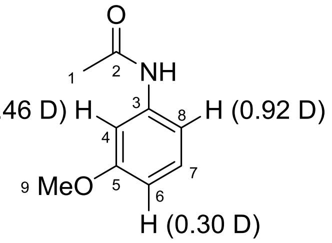

General Procedure C: The reaction afforded acetanilide *deuterio*-**5g** as an off-white solid; ^{1}H NMR ($CDCl_3$, 400 MHz): δ 7.51 (1H, br. s, N-H), 7.25 (0.54H, s, C**4**-H), 7.21-7.13 (1H, m, C**7**-H), 6.99-6.93 (0.08H, m, C**8**-H), 6.64 (0.70H, d, J = 8.5 Hz, C**6**-H), 3.77 (3H, s, C**9**-H_3), 2.15 (3H, s, C**1**-H_3); ^{2}H NMR (CH_2Cl_2, 500 MHz): δ 7.27 (0.46D, s, C**4**-D), 7.05 (0.92D, s, C**8**-D), 6.69 (0.30D, s, C**6**-D). *Deuterium incorporation was calculated by integration of ^{1}H NMR and ^{2}H NMR signals.*

***Deuterio*-6g**

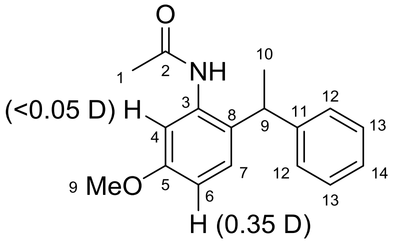

General Procedure C: The reaction afforded acetanilide *deuterio*-**6g** (0.9:0.1 mixture of rotamers *A*:*B*, >25:1 branched:linear) as a colorless solid; ^{1}H NMR ($CDCl_3$, 400 MHz): δ 7.43 (0.9H, s, C**4**-H, *A*), 7.40-7.03 (6.1H, m, ArC-H, *A*+*B*), 6.87-6.62 (1.65H, m, C**6**-H and N-H, *A*+*B*), 4.28-4.13 (0.1H, m, C**9**-H, *B*), 4.07 (0.9H, q, J = 7.0 Hz, C**9**-H, *A*), 3.78 (3H, s, C**15**-H_3, *A*+*B*), 1.91 (2.7H, s, C**1**-H_3, *A*), 1.67 (0.3H, br. s, C**1**-H_3, *B*), 1.59 (3H, d, J = 7.0 Hz, C**10**-H_3, *A*+*B*); ^{2}H NMR (CH_2Cl_2, 500 MHz): δ 6.81 (0.35D, s, C**6**-D). *Deuterium incorporation was calculated by integration of ^{1}H NMR signals of spectra recorded both in $CDCl_3$ and CD_3OD.*

***Deuterio*- N-Me-5a**

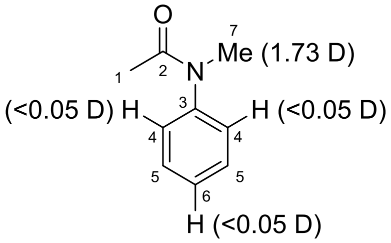

General Procedure C: The reaction afforded acetanilide *deuterio*- **N-Me-5a** as an off-white solid; ^{1}H NMR (DMSO-d_6, 400 MHz): δ 7.40 (2H, d, J = 7.5 Hz, C**4**-H), 7.36-7.21 (3H, m, C**5**-H and C**6**-H), 3.11 (1.27H, s, C**7**-H_3), 1.72 (3H, s, C**1**-H_3); ^{2}H NMR (DMSO, 500 MHz): δ 3.03 (1.73D, s, C**7**-D). *Deuterium incorporation was calculated by integration of ^{1}H NMR signals.*

Ligand Bite Angle and Electronic Effects

General procedure D for synthesis of fluorinated bidentate ligands

To a solution of the appropriate Grignard reagent (Ar^FMgBr) in anhydrous Et_2O (0.25 M) [freshly prepared from the corresponding Ar^FBr (615 mol%), and magnesium turnings (500 mol%)] was added *via* syringe a solution of corresponding bis(dichloro)phosphine (100 mol%) in Et_2O (1 M). The mixture was stirred at room temperature overnight. Water was then added (15 mL/mmol) and the mixture was filtered through a pad of Celite®, washing the cake with CH_2Cl_2 (3 × 10 mL/mmol). The filtrate was concentrated *in vacuo*, re-dissolved in CH_2Cl_2 (10 mL/mmol) and washed with water (10 mL/mmol) and brine (10 mL/mmol). The organic portion was dried over Na_2SO_4, filtered and the solvent was removed under reduced pressure. The crude mixture was re-dissolved in a minimum of

CH_2Cl_2 and filtered through a short pad of alumina. The filtrate was concentrated *in vacuo* to provide the corresponding bidentate ligand. Any further purification is indicated where appropriate.

<u>General procedure E</u> for preparation of Ir-complexes

Under an N_2 atmosphere, a flame-dried round-bottomed flask was charged with $[Ir(cod)_2]BARF$ (7.63 mg, 6.00 μmol, 100 mol%), bidentate phosphine ligand (6.00 μmol, 100 mol%) and anhydrous CH_2Cl_2 (1 mL). The reaction was stirred at room temperature for 3 hours and then the solvent was removed *in vacuo*. The resulting solid was dried under high vacuum for 1 hour to remove 1,4-cyclooctadiene. Purification by recrystallization (hexane/ CH_2Cl_2) afforded pure iridium complexes.

Preparation of 1,3-Bis[bis(pentafluorophenyl)phosphino]propane (d^{F}ppp)

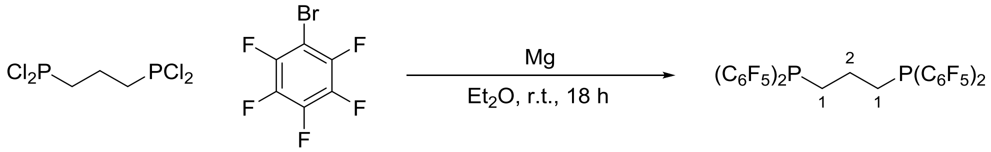

<u>General Procedure D:</u> Bromopentafluorobenzene (0.77 mL, 6.15 mmol), magnesium (122 mg, 5.00 mmol) and 1,3-bis(dichlorophosphanyl)propane (246 mg, 1.00 mmol) were employed in the reaction.[9] Purification by FCC (hexane → 5% EtOAc/hexane) afforded the d^{F}ppp ligand (463 mg, 60% yield) as an off white solid; ν_{max} / cm^{-1}: 1641 (m), 1515 (s), 1463 (s), 1383 (m), 1081 (s); ^{1}H NMR (400 MHz, $CDCl_3$): δ 2.68 – 2.63 (4H, m, 2 × C**1**-$\underline{H}_2$), 1.66 – 1.54 (2H, m, C**2**-$\underline{H}_2$); ^{13}C NMR (125 MHz, $CDCl_3$): δ 147.7 (d, $^1J_{C\text{-}F}$ = 247.0 Hz, $\underline{C}_{ortho}$-F), 142.5 (d, $^1J_{C\text{-}F}$ = 258.0 Hz, $\underline{C}_{para}$-F), 137.6 (d, $^1J_{C\text{-}F}$ = 256.0 Hz, $\underline{C}_{meta}$-F), 108.2 (m, C_q), 24.6 (m, C**1**), 23.3 (t, $^2J_{C\text{-}P}$ = 24.0 Hz, C**2**); ^{19}F NMR (377 MHz, $CDCl_3$): δ – 130.0 (8F, m, C_{ortho}-$\underline{F}$), – 148.9 (4F, tt, *J* = 3.5, 20.0 Hz, C_{para}-$\underline{F}$), – 159.5 (8F, m, C_{meta}-$\underline{F}$); ^{31}P{^{1}H} NMR (123 MHz, $CDCl_3$): δ – 47.1 (tt, *J* = 11.0, 27.0 Hz); HRMS: (EI$^+$) Calculated for $C_{27}H_6F_{20}P_2$: 771.9625 Found $[M]^+$: 771.9628; m.p.: 104 – 105 °C (hexane/CH_2Cl_2).

[Ir(cod)d^{F}ppe]BARF

The complex was characterized by single crystal X-ray diffraction of crystals obtained from CH_2Cl_2-hexane (Figure 3) ***(CCDC 1413028)***.

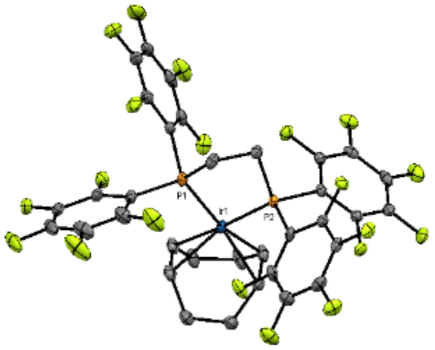

Figure 3: ORTEP view of [Ir(cod)d^{F}ppe]BARF complex (hydrogens atoms and BARF counterion are omitted for clarity).

[Ir(cod)d^{F}ppb]BARF

The complex was characterized by single crystal X-ray diffraction of crystals obtained from CH_2Cl_2-hexane (Figure 4) ***(CCDC 1413029)***.

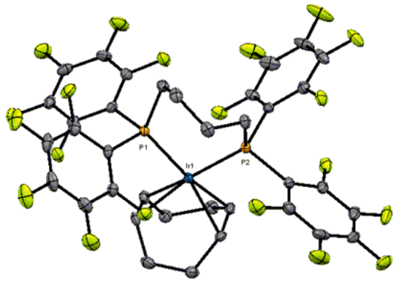

Figure 4: ORTEP view of [Ir(cod)d^{F}ppb]BARF complex (hydrogens atoms and BARF counterion are omitted for clarity).

Estimation of ligand bite angles:

The graph in Scheme 3 (main paper) uses average bite angle values reported in reference 20 (main paper) for dppm (72°), dppe (85°), dppp (91°), dppb (98°). Bite angles were determined for d^{F}ppe (83.4^o) and d^{F}ppb (94.0^o) by analysis of the X-ray structures shown above. These values are, on average, 97% the size of the reported values for dppe and dppb. Extrapolation of this trend provided estimated values for d^{F}ppm and d^{F}ppp of 69.8^o and 88.3^o, respectively.

Results for ligand effect studies:

Yields and selectivities were determined by ^{1}H NMR using 1,3,5-trimethoxybenzene as an internal standard.

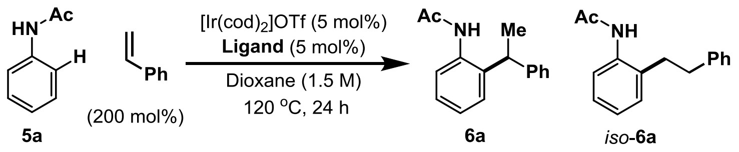

entry	Ligand	β (°)	Conversion (%)	6a (%)	*iso*-6a (%)	Branch selectivity (%)
1	dppm	72	**14**	**<5**	**14**	**0**
2	dppe	85	**48**	**6**	**42**	**13**
3	dppp	91	**44**	**13**	**31**	**30**
4	dppb	98	**20**	**16**	**4**	**80**
5	d^{F}ppm	69.8	**49**	**20**	**29**	**41**
6	d^{F}ppe	83.4	**82**	**53**	**29**	**65**
7	d^{F}ppp	88.3	**85**	**79**	**6**	**93**
8	d^{F}ppb	94.0	**85**	**85**	<5	**100**

Product Derivatizations

N-(2-Bromo-2-(1-phenylethyl)phenyl)acetamide (10)

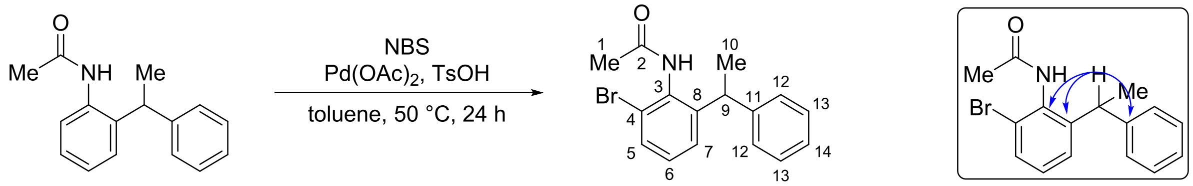

HMBC analysis

The title compound was prepared following a literature procedure.[10] A re-sealable tube was charged with **6a** (25.0 mg, 0.10 mmol, 100 mol%), *N*-bromosuccinimide (20.4 mg, 0.11 mmol, 110 mol%), $Pd(OAc)_2$ (1.17 mg, 5 mol%), *p*-toluenesulfonic acid monohydrate (9.93 mg, 0.05 mmol, 50 mol%) and toluene (0.4 mL). The tube was sealed and the mixture was heated at 50 °C for 24 hours. The reaction was cooled to room temperature, diluted with EtOAc (5 mL), and washed with saturated aq. Na_2CO_3 (2 × 2 mL) and brine (3 mL). The organic layer was dried over Na_2SO_4 and concentrated *in vacuo* to provide the crude material. Purification by FCC (10% EtOAc/hexane → 30% EtOAc/hexane) afforded acetanilide **10** (28.0 mg, 88% yield, 0.9:0.1 mixture of rotamers *A*:*B*, 7:1 *ortho*:*para* bromination) as a colorless wax; ν_{max} / cm^{-1}: 3241 (s), 3025 (m), 2967 (s), 2931 (m), 1662 (s), 1518 (s), 1444 (s), 1371 (s), 1283 (s), 1028 (s); ^{1}H NMR ($CDCl_3$, 400 MHz, *ortho-brominated product signals only*): δ 7.50 (0.9H, d, *J* = 8.0 Hz, C**5**-H, *A*), 7.44 (0.1H, d, *J* = 8.0 Hz, C**5**-H, *B*), 7.39-6.97 (7H, m, ArC-H, *A*+*B*), 6.57 (0.9H, br. s, N-H, *A*), 6.44 (0.1H, br. s, N-H, *B*), 4.39 (0.1H, q, *J* = 7.0 Hz, C**9**-H, *B*), 4.31 (0.9H, q, *J* = 7.0 Hz, C**9**-H, *A*), 2.13 (2.7H, s, C**1**-H_3, *A*), 1.80 (0.3H, s, C**1**-H_3, *B*), 1.61 (0.3H, d, *J* = 7.0 Hz, C**10**-H_3, *B*), 1.56 (2.7H, d, *J* = 7.0 Hz, C**10**-H_3, *A*); ^{13}C NMR ($CDCl_3$, 100 MHz, *major rotamer signals only*): δ 168.9 (C**2**), 146.1 (C**8**), 145.6 (C**11**), 133.8 (C**3**), 131.0 (C**5**), 129.1 (C**6**), 128.6 (C**13**), 127.3 (C**12**), 126.9 (C**7**), 126.3 (C**14**), 124.1 (C**4**), 40.9 (C**9**),

23.2 (C**1**), 21.6 (C**10**); HRMS: (ESI$^+$) Calculated for $C_{16}H_{17}(^{79}Br)NO$: 318.0488. Found $[M+H]^+$: 318.0489. *The structure of compound **10** was confirmed by HMBC (as indicated above). For spectroscopic data for the corresponding para-brominated product see compound **6m**.*

2-Methyl-4-(1-phenylethyl)-1-(*o*-tolyl)-1*H*-benzo[*d*]imidazole (11)

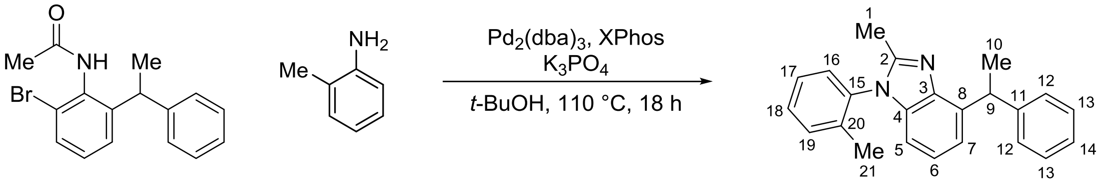

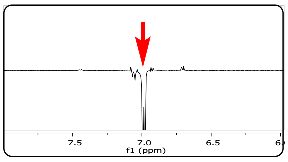

nOe analysis

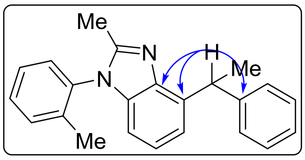

HMBC analysis

The title compound was prepared following a literature procedure.[11] An oven-dried re-sealable tube, fitted with a magnetic stirrer, was charged with acetanilide **10** (23.3 mg, 0.07 mmol, 100 mol%), $Pd_2(dba)_3$ (0.67 mg, 1 mol%), XPhos (2.79 mg, 8 mol%) and K_3PO_4 (38.9 mg, 0.18 mmol, 250 mol%). The tube was fitted with a rubber septum and purged with nitrogen. Freshly distilled *o*-toluidine (12.0 µL, 0,110 mmol, 150 mol%), and anhydrous *t*-BuOH (0.15 mL) were added *via* syringe and the tube was sealed with a Young's tap. The reaction vessel was placed into a pre-heated heating block at 110 °C and stirred for 18 hours. The reaction mixture was cooled to room temperature and diluted with CH_2Cl_2 (5 mL). The solution was filtered through a short pad of Celite® and the filtrate was concentrated *in vacuo*. Purification of the residue by FCC (20% EtOAc/hexane → 30% EtOAc/hexane) afforded pure benzimidazole **11** (21.3 mg, 89% yield, 0.55:0.45 mixture of rotamers *A*:*B*) as an off-white solid; ν_{max} / cm^{-1}: 3675 (s), 2987 (m), 2972 (s), 2901 (m), 1394 (s), 1264 (s), 1056 (s); ^{1}H NMR ($CDCl_3$, 400 MHz): δ 7.55-7.13 (9H, m, ArC-H, *A*+*B*), 7.11-7.03 (1H, m, C**6**-H, *A*+*B*), 6.99 (0.55H, d, *J* = 7.5 Hz, C**7**-H, *A*), 6.94 (0.45H, d, *J* = 7.5 Hz, C**7**-H, *B*), 6.71 (1H, d, *J* = 7.5 Hz, C**5**-H, *A*+*B*), 5.16 (1H, q, *J* = 7.0 Hz, C**9**-H, *A*+*B*), 2.41 (1.35H, s, C**1**-H_3, *B*), 2.40 (1.65H, s, C**1**-H_3, *A*), 2.00 (1.65H, s, C**21**-H_3, *A*), 1.98 (1.35H, s, C**21**-H_3, *B*), 1.80 (3H, d, *J* = 7.0 Hz, C**10**-H_3, *A*+*B*); ^{13}C NMR ($CDCl_3$, 100 MHz): δ 150.9 (*2 signals*, C**2**, *A*+*B*), 146.1 (C**11**, *B*), 145.8 (C**11**, *A*), 141.0 (*2 signals*, C**3**, *A*+*B*), 137.8 (C**8**, *B*), 137.6 (C**8**, *A*), 136.4 (*2 signals*, C**15**, *A*+*B*), 136.0 (*2 signals*, C**4**, *A*+*B*), 135.0 (*2 signals*, C**20**, *A*+*B*), 131.4 (*2 signals*, C**19**, *A*+*B*), 129.5 (*2 signals*, C**18**, *A*+*B*), 128.4 (*2 signals*, C**17**, *A*+*B*), 128.2 (*2 signals*, C**13**, *A*+*B*), 128.0 (C**12**, *B*), 127.9 (C**12**, *A*), 127.3 (*2 signals*, C**14**, *A*+*B*), 125.9 (C**16**, *B*), 125.8 (C**16**, *A*), 122.5 (*2 signals*, C**6**, *A*+*B*), 119.9 (C**7**, *B*), 119.8 (C**7**, *A*), 107.6 (*2 signals*, C**5**, *A*+*B*), 39.0 (C**9**, *B*), 38.9 (C**9**, *A*), 21.6 (C**10**, *B*), 21.5 (C**10**, *A*), 17.4 (*2 signals*, C**21**, *A*+*B*), 14.1 (*2 signals*, C**1**, *A*+*B*); HRMS: (ESI$^+$) Calculated for $C_{23}H_{23}N_2$: 327.1856. Found $[M+H]^+$: 327.1861; m.p. = 94-96 ˚C (hexane/CH_2Cl_2). *The structure of compound*

*11 was confirmed by HMBC analysis (as indicated above). The product structure was further confirmed by the presence of the characteristic C**2** peaks at 150.9 ppm in ^{13}C NMR spectrum. Selective irradiation of signal for C7-<u>H</u> (as shown above) of rotamer B in a 1D gradient nOe experiment revealed a negative peak for the respective signal of rotamer A.*

2-(1-Phenylethyl)aniline (12)

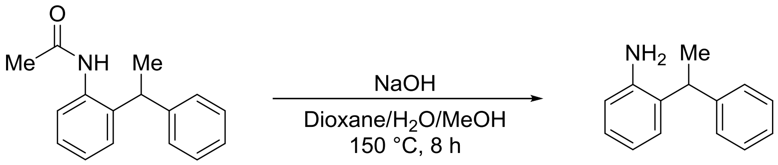

A re-sealable tube was charged with **6a** (100.0 mg, 0.42 mmol, 100 mol%), NaOH (669 mg, 16.71 mmol, 4000 mol%), 1,4-dioxane (3 mL), water (3 mL) and MeOH (3 mL). The tube was sealed and the reaction was stirred at 150 °C for 8 hours. The mixture was cooled to room temperature and the solvent was removed *in vacuo*. The residue was re-dissolved in water (5 mL) and extracted with EtOAc (2 mL). The aqueous phase was acidified with 1M *aq.* HCl to pH ≈ 3 and extracted again with EtOAc (3 × 5 mL). The combined organic extracts were dried over Na_2SO_4 and concentrated *in vacuo* to provide the crude material. Purification by FCC (40% EtOAc/hexane → 50% EtOAc/hexane) afforded aniline **12** (77.1 mg, 93% yield) as an off-white solid; ^{1}H NMR ($CDCl_3$, 400 MHz): δ 7.37-7.15 (6H, m), 7.10 (1H, ddd, *J* = 1.5, 7.5, 7.5 Hz), 6.86 (1H, ddd, *J* = 1.5, 7.5, 7.5 Hz), 6.65 (1H, dd, *J* = 1.5, 7.5 Hz), 4.09 (1H, q, *J* = 7.0 Hz), 3.43 (2H, br. s), 1.64 (3H, s); ^{13}C NMR ($CDCl_3$, 100 MHz): δ 145.6, 144.3, 129.8, 128.7, 127.4, 127.3, 127.2, 126.4, 118.7, 116.2, 40.2, 21.8, m.p. = 56-58 ˚C [CH_2Cl_2/hexane] (Lit.[12a] 58-59 ˚C, petroleum ether). *The spectroscopic proprieties were consistent with the data available in literature.*[12b]

Ethyl (*Z*)-3-((2-(1-phenylethyl)phenyl)amino)but-2-enoate

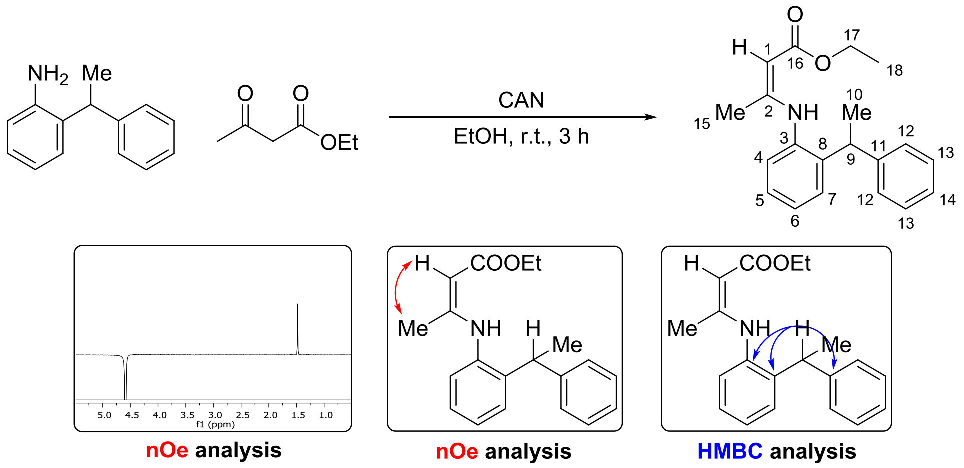

The title compound was prepared following a literature procedure.[13] To a stirred solution of **12** (50.0 mg, 0.25 mmol, 100 mol%) and ethyl acetoacetate (32.0 µL, 0.25 mmol, 100 mol%) in EtOH (0.5

mL) was added ceric ammonium nitrate (6.95 mg, 0.01 mmol, 5 mol%) in one portion. The mixture was stirred at room temperature for 3 hours. The reaction was diluted with CH_2Cl_2 (5 mL) and washed with water (1 mL). The organic layer was dried over Na_2SO_4 and concentrated *in vacuo* to provide the crude material. Purification by FCC (5% EtOAc/hexane → 10% EtOAc/hexane) afforded the desired enamine (51.6 mg, 67% yield) as a yellow oil; ν_{max} / cm^{-1}: 3685 (s), 3225 (m), 2987 (m), 2972 (s), 2901 (m), 1611 (s), 1594 (s), 1393 (s), 1264 (s), 1056 (s); ^1H NMR ($CDCl_3$, 400 MHz): δ 10.08 (1H, br. s, N-H), 7.37 (1H, dd, *J* = 1.5, 7.5 Hz, C7-H), 7.34-7.09 (7H, m, ArC-H), 7.04 (1H, dd, *J* = 1.5, 7.5 Hz, C**4**-H), 4.60 (1H, s, C**1**-H), 4.42 (1H, q, *J* = 7.0 Hz, C**9**-H), 4.17 (2H, q, *J* = 7.0 Hz, C**17**-H_2), 1.61 (3H, d, *J* = 7.0 Hz, C**10**-H_3), 1.48 (3H, s, C**15**-H_3), 1.31 (3H, t, *J* = 7.0 Hz, C**18**-H_3); ^{13}C NMR ($CDCl_3$, 100 MHz): δ 170.6 (C**16**), 160.3 (C**2**), 145.3 (C**11**), 142.8 (C**8**), 137.3 (C**3**), 128.3 (C**13**), 128.2 (C**4**), 127.6 (C**12**), 127.5 (C**7**), 126.8 (C**6**), 126.6 (C**5**), 126.0 (C**14**), 84.8 (C**1**), 58.6 (C**17**), 39.8 (C**9**), 21.5 (C**10**), 19.6 (C**15**), 14.6 (C**18**); HRMS: (ESI^+) Calculated for $C_{20}H_{24}NO_2$: 310.1802. Found $[M+H]^+$: 310.1801. *The structure of the title compound was confirmed by HMBC analysis (as indicated above). The geometry of the double bond was confirmed by 1D gradient nOe analysis (as shown above): selective irradiation of signal for C1-H revealed a positive peak for C**15**-H_3 signal.*

Ethyl 2-methyl-7-(1-phenylethyl)-1*H*-indole-3-carboxylate (13)

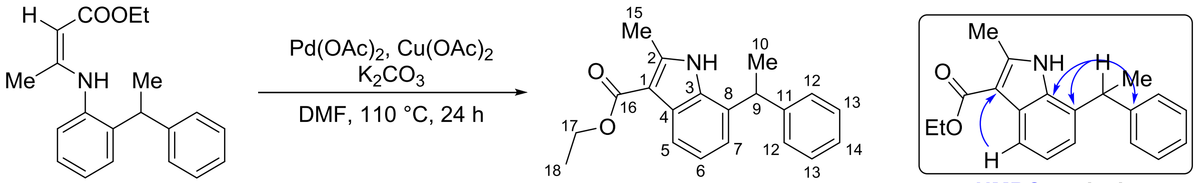

HMBC analysis

The title compound was prepared following a literature procedure.[14] An oven-dried re-sealable tube, fitted with a magnetic stirrer, was charged with ethyl (*Z*)-3-((2-(1-phenylethyl)phenyl)amino)but-2-enoate (47.6 mg, 0.15 mmol, 100 mol%), $Pd(OAc)_2$ (3.45 mg, 10 mol%), $Cu(OAc)_2$ (83.8 mg, 0.46 mmol, 300 mol%) and K_2CO_3 (63.8 mg, 0.46 mmol, 300 mol%). The tube was fitted with a rubber septum and purged with nitrogen. Anhydrous DMF (1.9 mL) was added *via* syringe and the tube was sealed with a Young's tap. The reaction vessel was placed into a pre-heated heating block at 110 °C and stirred for 24 hours. The reaction mixture was cooled to room temperature and diluted with EtOAc (5 mL). The solution was filtered through a short pad of SiO_2, washing exhaustively with EtOAc (20 mL), and the filtrate was concentrated *in vacuo*. Purification of the residue by FCC (20% EtOAc/hexane → 30% EtOAc/hexane) afforded pure indole **13** (35.3 mg, 77% yield) as a brown solid; ν_{max} / cm^{-1}: 3675 (s), 3447 (m), 2987 (m), 2972 (s), 2901 (m), 1688 (s), 1393 (s), 1264 (s), 1066 (s); ^1H NMR ($CDCl_3$, 400 MHz): δ 8.02 (1H, dd, *J* = 1.5, 7.5 Hz, C**5**-H), 7.91 (1H, br. s, N-H), 7.37-7.15 (7H, m, ArC-H), 4.48-4.23 (3H, m, C**9**-H and C**17**-H_2), 2.57 (3H, s, C**15**-H_3), 1.74 (3H, d, *J* = 7.0 Hz, C**10**-H_3), 1.42 (3H, t, *J* = 7.0 Hz, C**18**-H_3); ^{13}C NMR ($CDCl_3$, 100 MHz): δ 166.1 (C**16**), 145.4 (C**11**), 143.4 (C**2**), 133.1 (C**3**), 128.9 (C**13**), 127.7 (C**4**), 127.5 (C**8**), 127.3 (C**12**), 126.6 (C**14**), 121.8

(C**6**), 120.5 (C**7**), 119.7 (C**5**), 104.7 (C**1**), 59.4 (C**17**), 40.9 (C**9**), 21.4 (C**10**), 14.6 (C**18**), 14.2 (C**15**); HRMS: (ESI$^+$) Calculated for $C_{20}H_{22}NO_2$: 308.1645. Found $[M+H]^+$: 308.1645; m.p. = 119-120 ˚C (hexane/CH_2Cl_2). *The structure of compound **13** was confirmed by HMBC analysis (as indicated above).*

8-(1-Phenylethyl)quinoline (14)

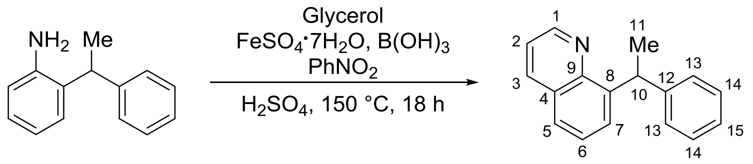

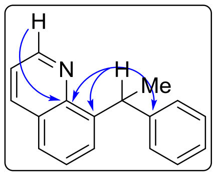

HMBC analysis

The title compound was prepared following a literature procedure.[15] A re-sealable tube was charged with **12** (300 mg, 1.52 mmol, 100 mol%), $FeSO_4{\cdot}7H_2O$ (50.7 mg, 0.18 mmol, 12 mol%), $PhNO_2$ (90.0 μL, 0.91 mmol, 60 mol%), boric acid (94.0 mg, 1.52 mmol, 100 mol%) and glycerol (560 mg, 6.08 mmol, 400 mol%). The mixture was cooled to 0 °C and conc. H_2SO_4 (0.3 mL) was added dropwise. The resulting solution was warmed room temperature, the tube was sealed and the mixture was heated at 150 °C for 18 hours. The reaction was cooled to room temperature and poured into ice-water (5 mL). The solution was basified with NaOH pellets to pH ≈ 10 and extracted with Et_2O (3 × 5 mL). The combined organic extracts were washed with brine (2 mL), dried over Na_2SO_4 and concentrated *in vacuo* to provide the crude material. Purification by FCC (5% EtOAc/hexane → 10% EtOAc/hexane) afforded quinoline **14** (239 mg, 68% yield) as an amber viscous oil; ν_{max} / cm^{-1}: 3675 (s), 3474 (m), 3224 (m), 2987 (m), 2972 (s), 2901 (m), 1393 (s), 1264 (s), 1056 (s); ^{1}H NMR ($CDCl_3$, 400 MHz): δ 8.96 (1H, dd, *J* = 2.0, 4.0 Hz, C**1**-H), 8.13 (1H, dd, *J* = 2.0, 8.5 Hz, C**3**-H), 7.69-7.61 (1H, m, C**5**-H), 7.52-7.42 (2H, m, C**6**-H and C**7**-H), 7.42-7.34 (3H, m, C**2**-H and C**13**-H), 7.28 (2H, dd, *J* = 7.5, 7.5 Hz, C**14**-H), 7.18 (1H, t, *J* = 7.5 Hz, C**15**-H), 5.73 (1H, q, *J* = 7.0 Hz, C**10**-H), 1.74 (3H, d, *J* = 7.0 Hz, C**11**-H_3); ^{13}C NMR ($CDCl_3$, 100 MHz): δ 149.4 (C**1**), 146.5 (C**12**), 146.1 (C**9**), 145.6 (C**8**), 136.3 (C**3**), 128.4 (C**4**), 128.1 (C**14**), 128.0 (C**13**), 127.4 (C**7**), 126.4 (C**6**), 126.0 (C**5**), 125.8 (C**15**), 120.9 (C**2**), 37.6 (C**10**), 21.5 (C**11**); HRMS: (ESI$^+$) Calculated for $C_{17}H_{16}N$: 234.1277. Found $[M+H]^+$: 234.1276. *The structure of compound **14** was confirmed by HMBC analysis (as indicated above). The product structure was further confirmed by the presence of the characteristic C**1** peak at 149.4 ppm in ^{13}C NMR spectrum.*

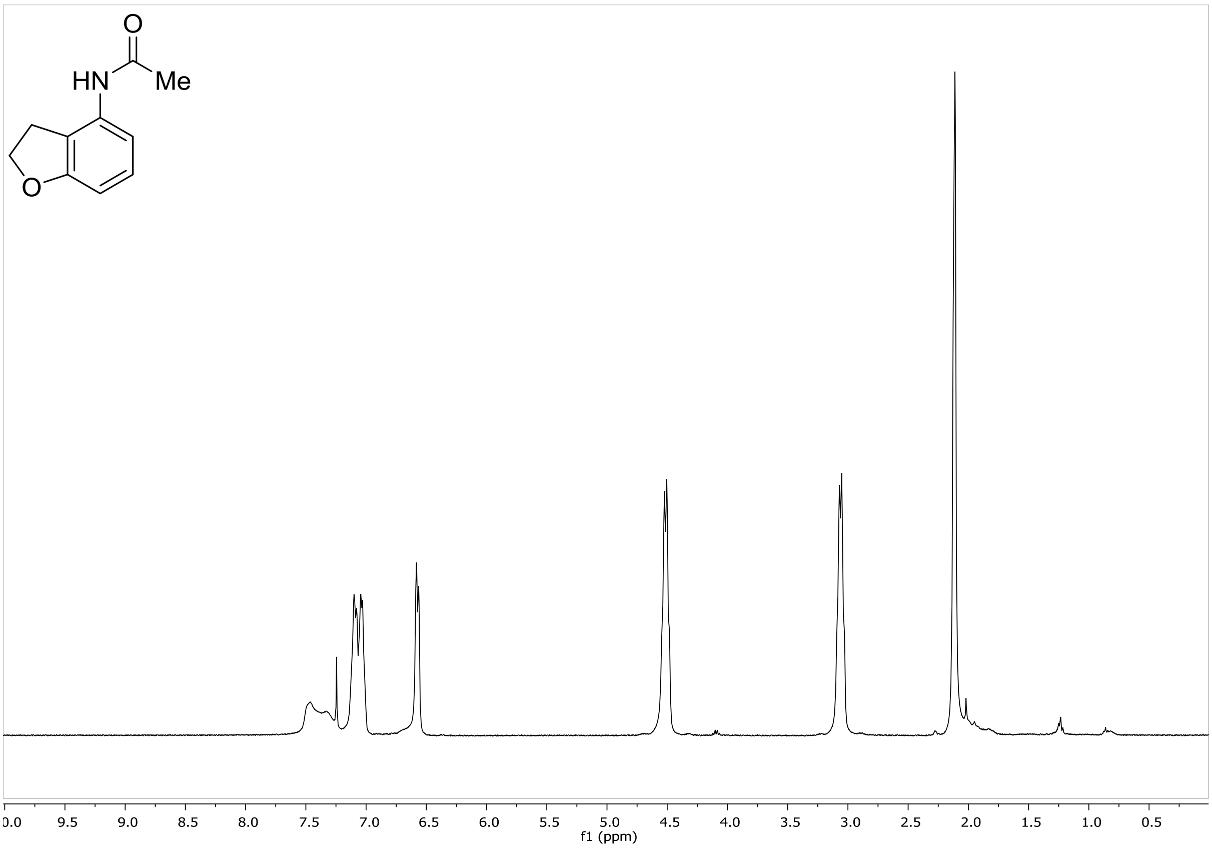

O
HN
Me
O
0.0 9.5 9.0 8.5 8.0 7.5 7.0 6.5 6.0 5.5 5.0 4.5 4.0 3.5 3.0 2.5 2.0 1.5 1.0 0.5
f1 (ppm)

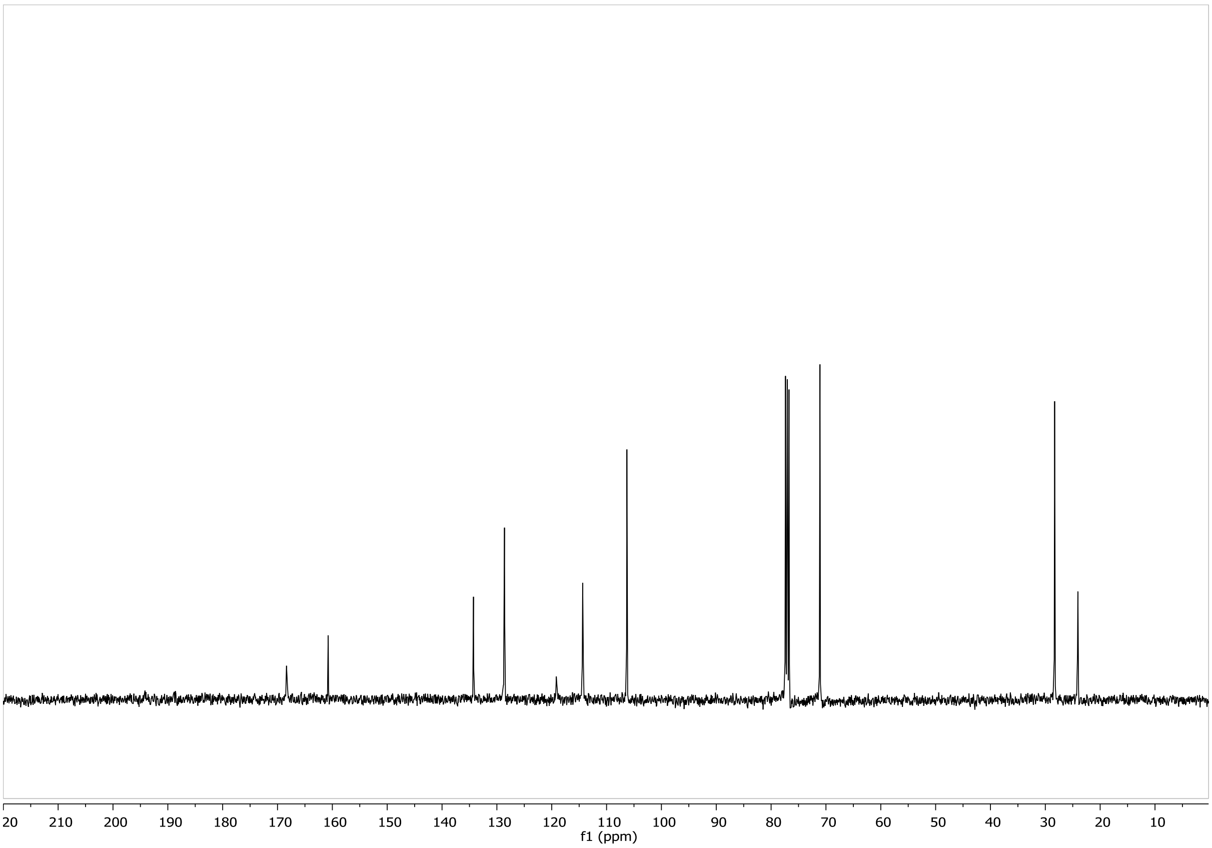

20 210 200 190 180 170 160 150 140 130 120 110 100 90 80 70 60 50 40 30 20 10
f1 (ppm)

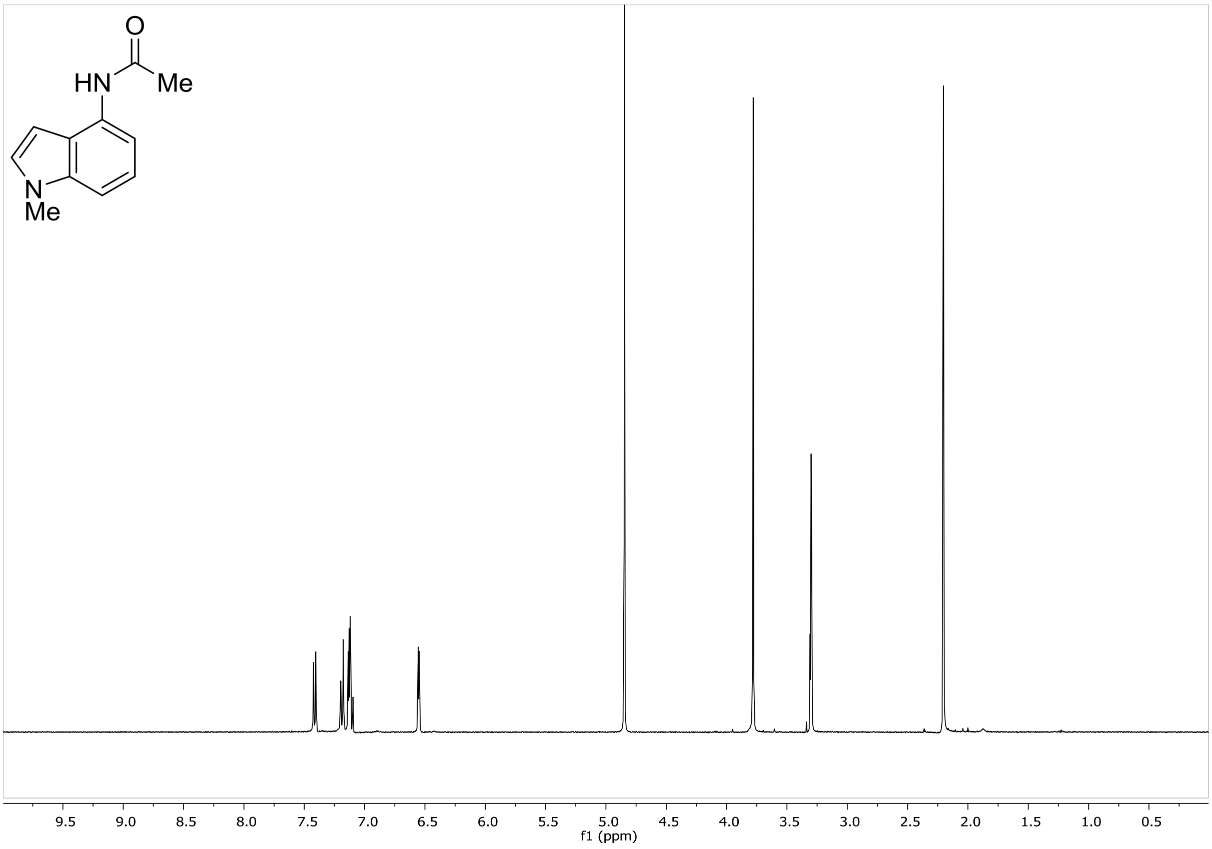
O
HN
Me
N
Me
9.5 9.0 8.5 8.0 7.5 7.0 6.5 6.0 5.5 5.0 4.5 4.0 3.5 3.0 2.5 2.0 1.5 1.0 0.5
f1 (ppm)

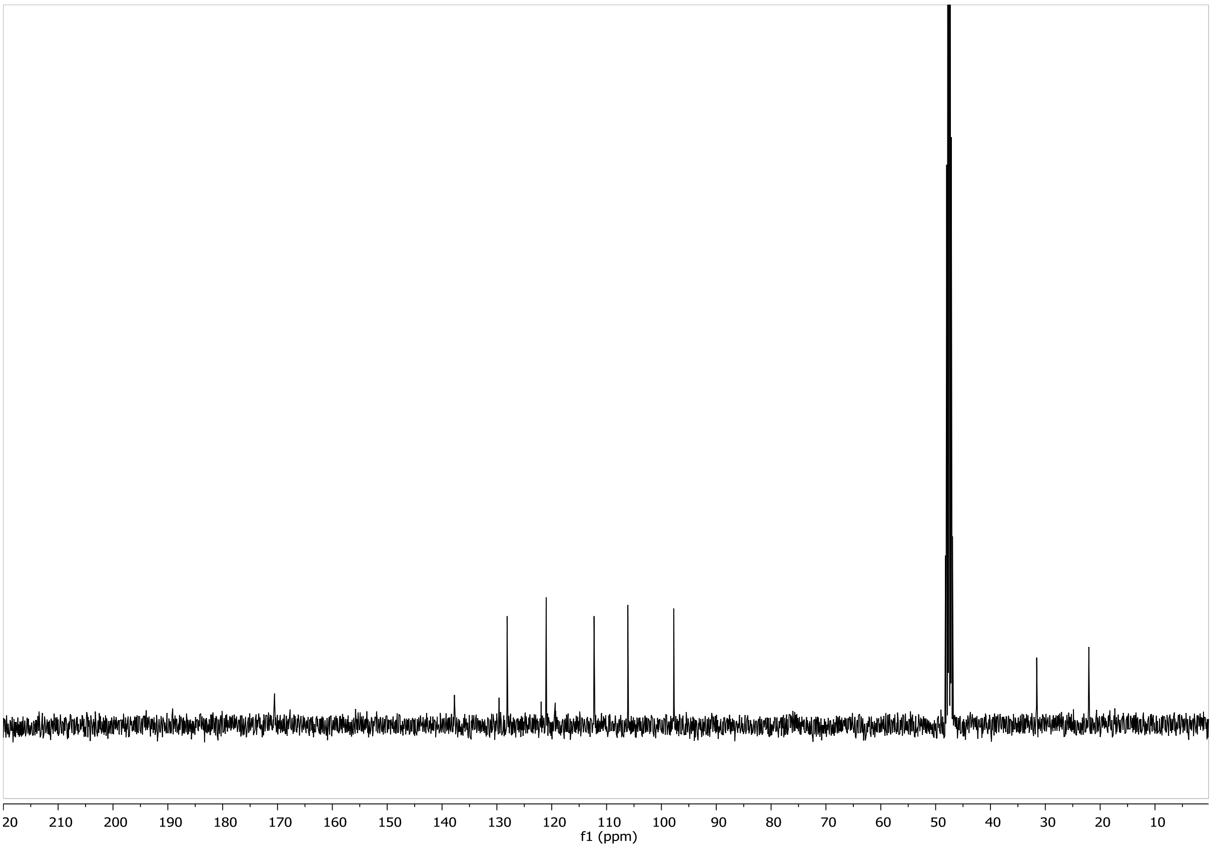
20 210 200 190 180 170 160 150 140 130 120 110 100 90 80 70 60 50 40 30 20 10
f1 (ppm)

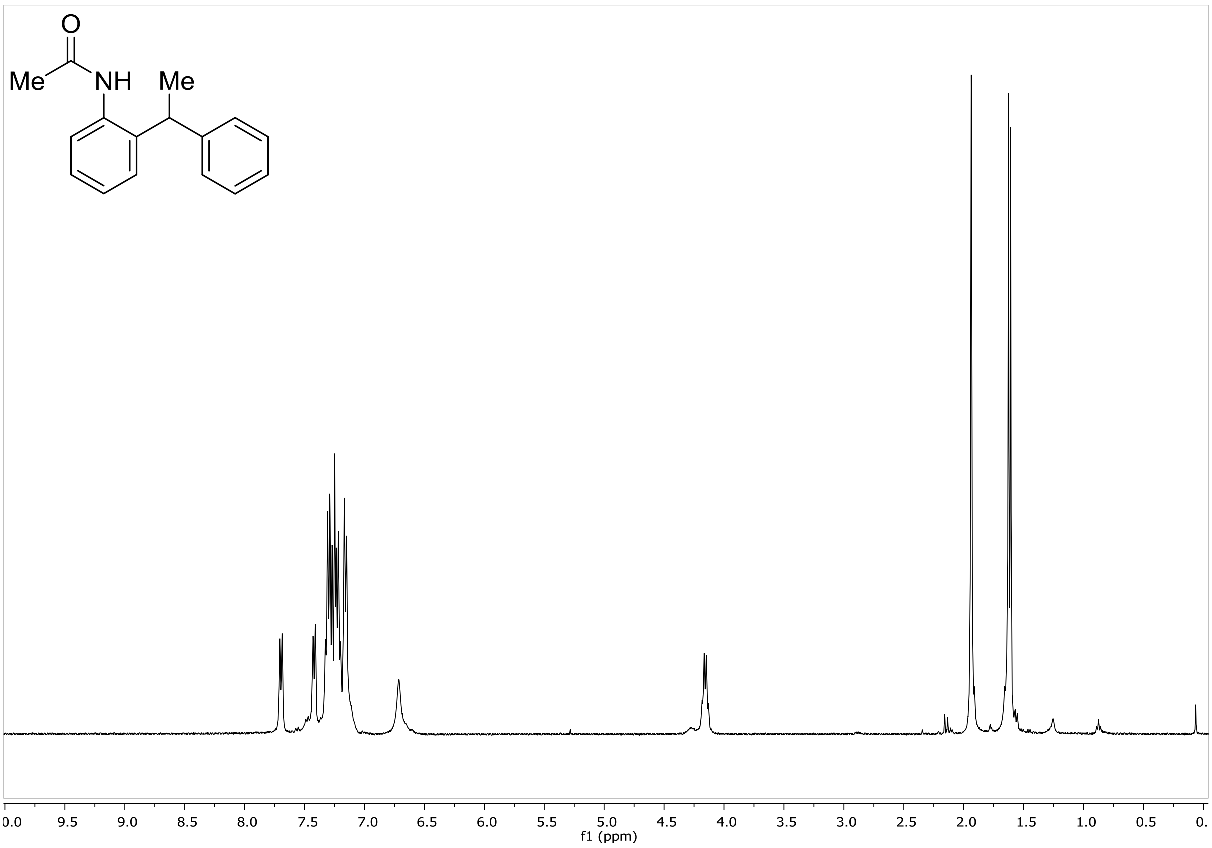
O
Me
NH
Me
f1 (ppm)

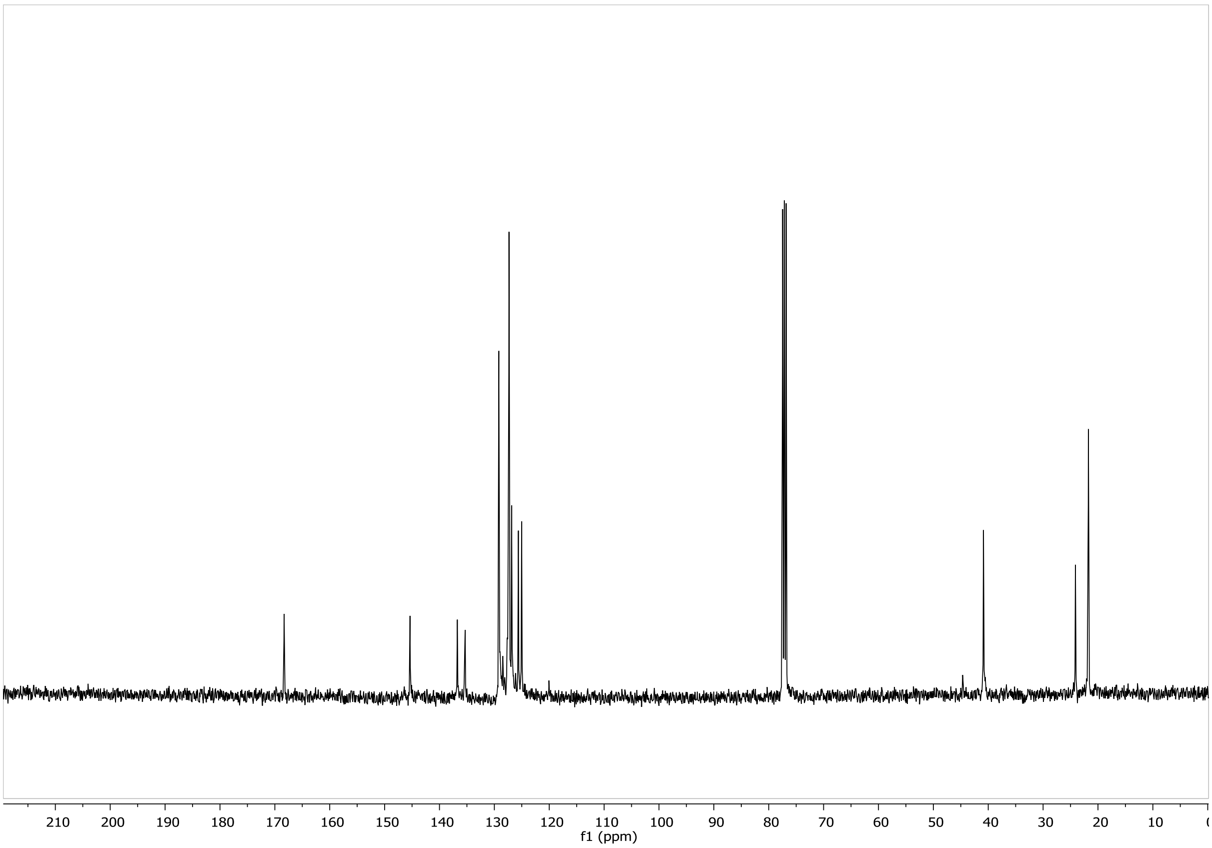
f1 (ppm)

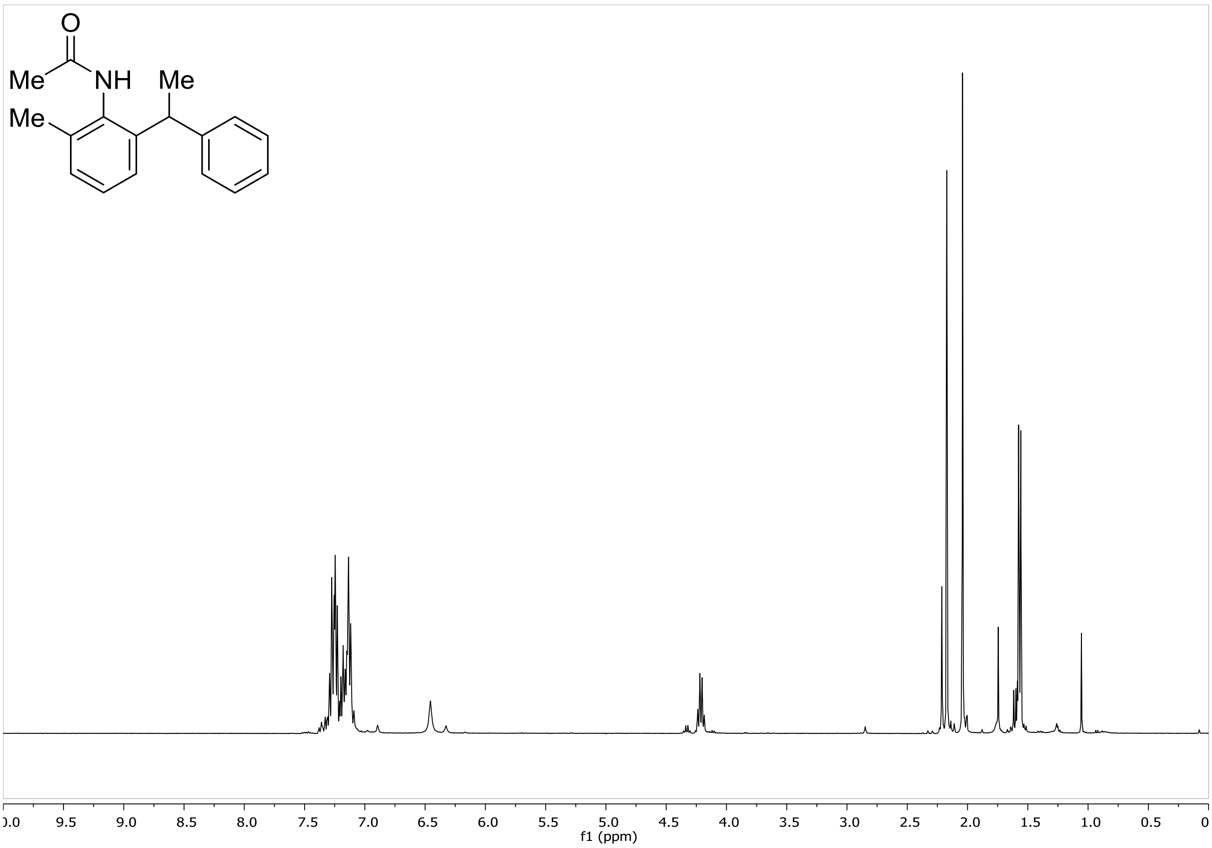
O
Me
NH
Me
Me
f1 (ppm)

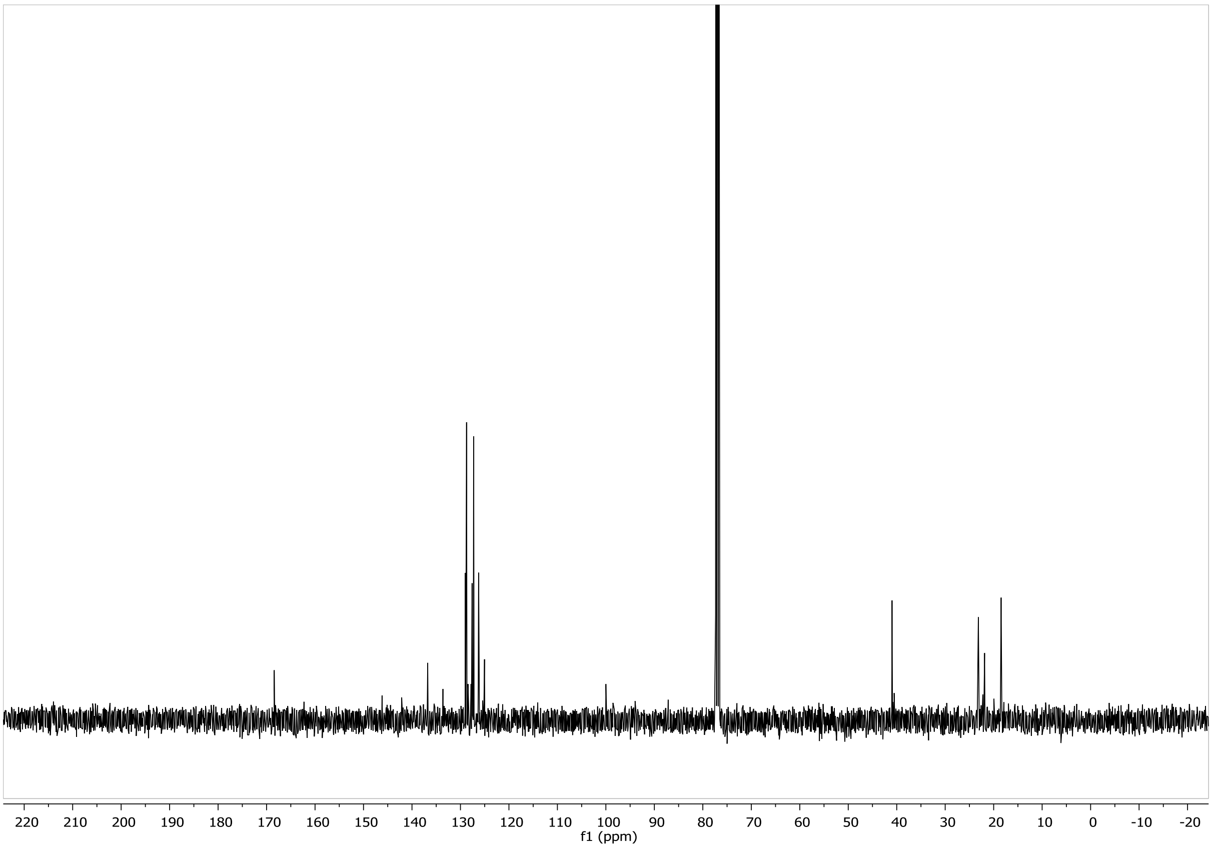
f1 (ppm)

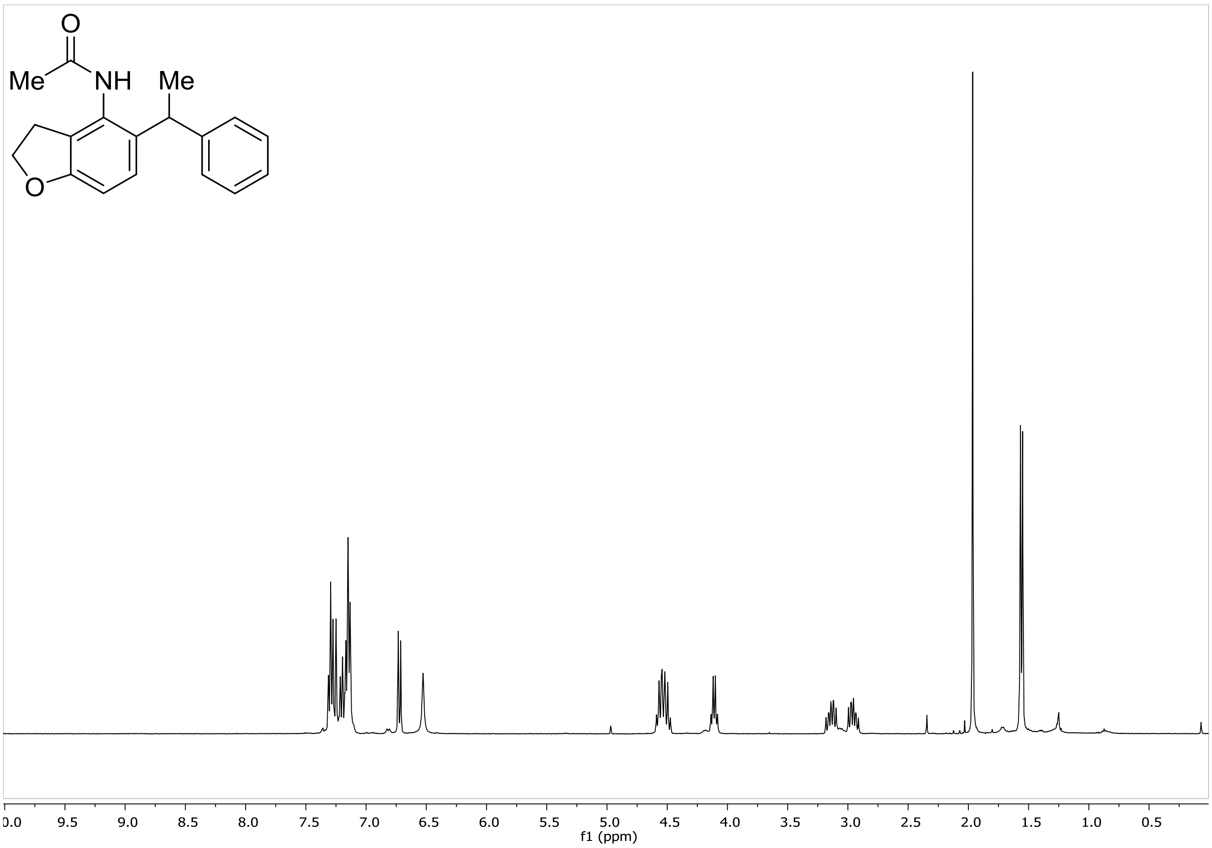
O
Me
NH
Me
O
f1 (ppm)

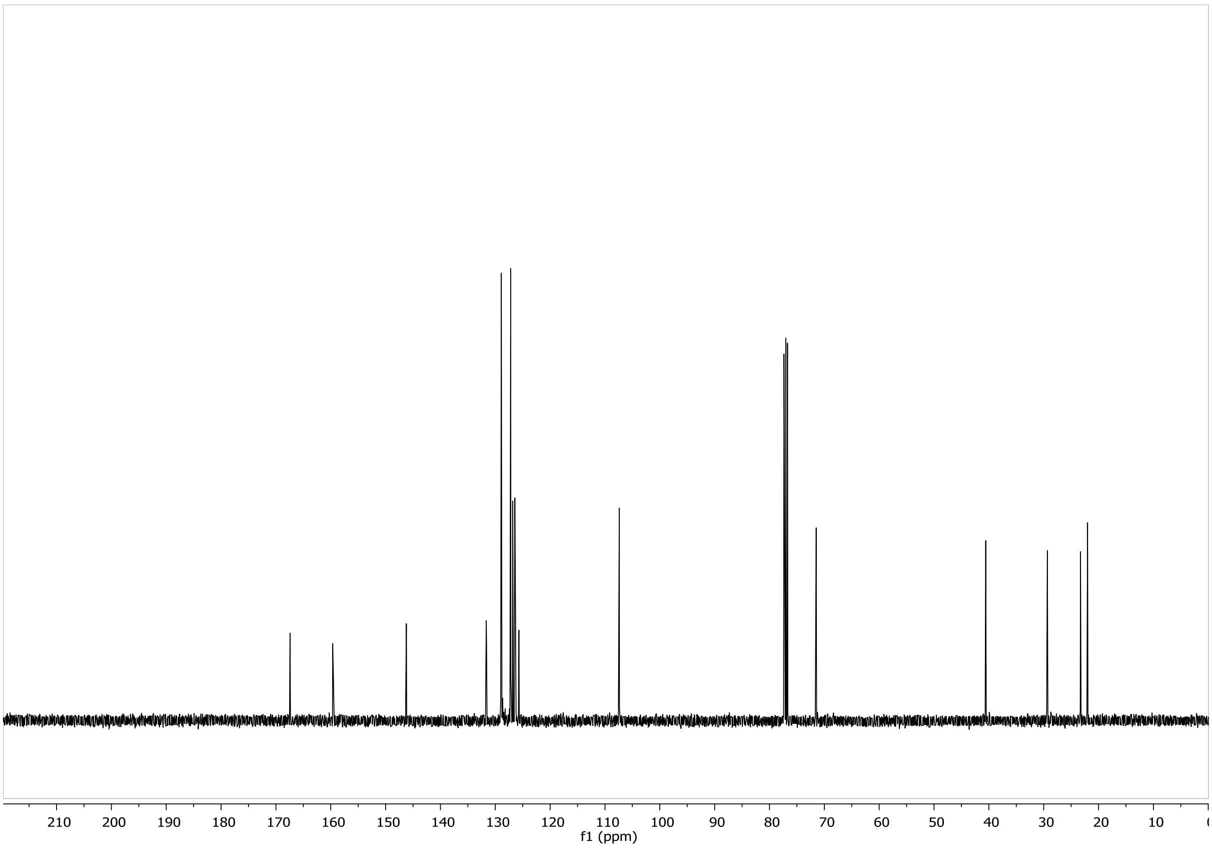
f1 (ppm)

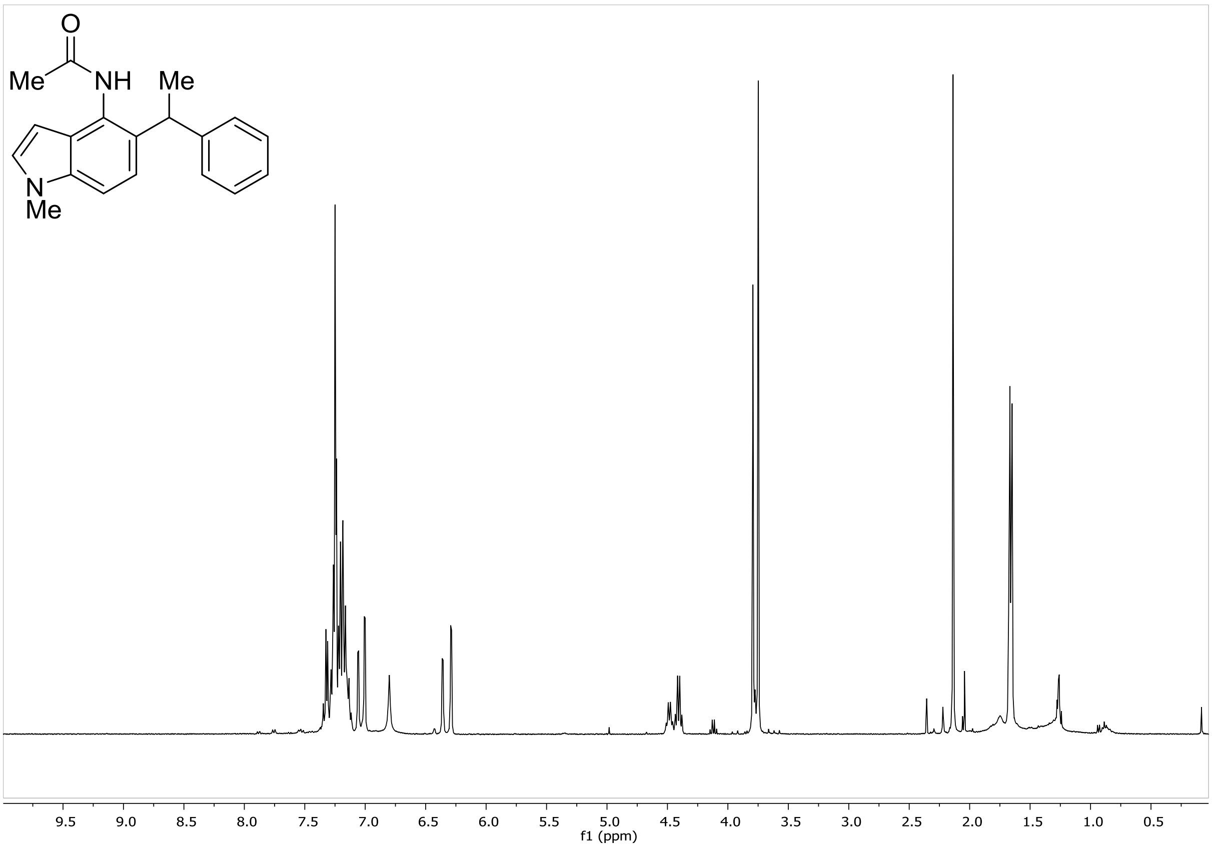
O
Me
NH
Me
N
Me
9.5 9.0 8.5 8.0 7.5 7.0 6.5 6.0 5.5 5.0 4.5 4.0 3.5 3.0 2.5 2.0 1.5 1.0 0.5
f1 (ppm)

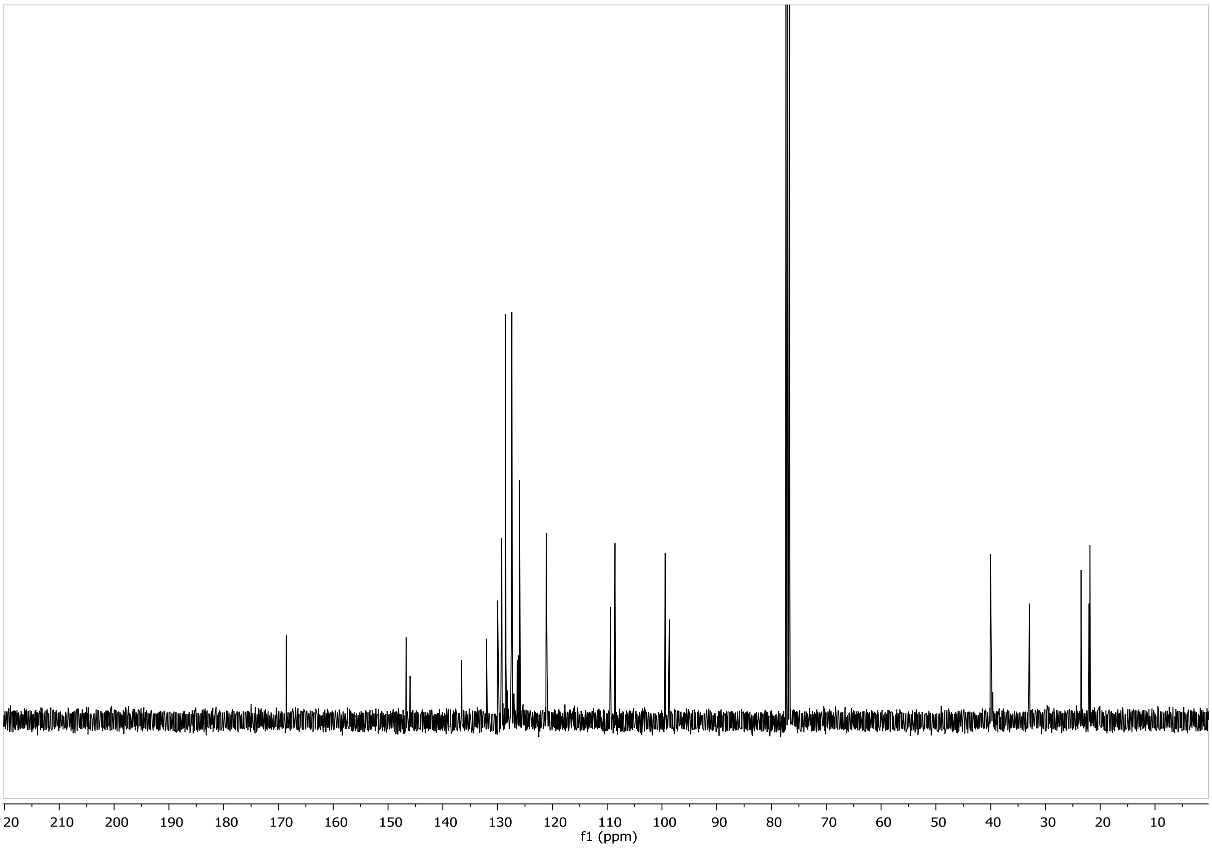
20 210 200 190 180 170 160 150 140 130 120 110 100 90 80 70 60 50 40 30 20 10
f1 (ppm)

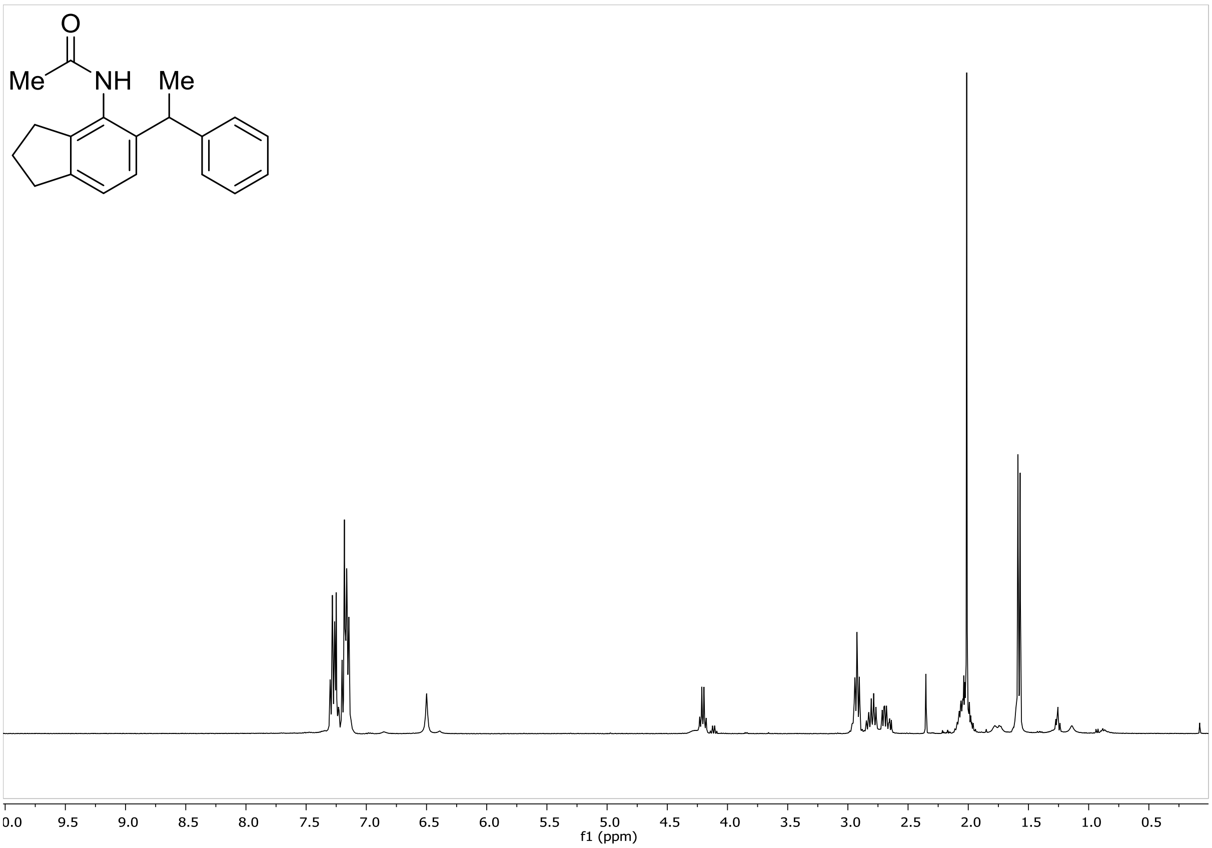
O
Me
NH
Me
0.0 9.5 9.0 8.5 8.0 7.5 7.0 6.5 6.0 5.5 5.0 4.5 4.0 3.5 3.0 2.5 2.0 1.5 1.0 0.5
f1 (ppm)

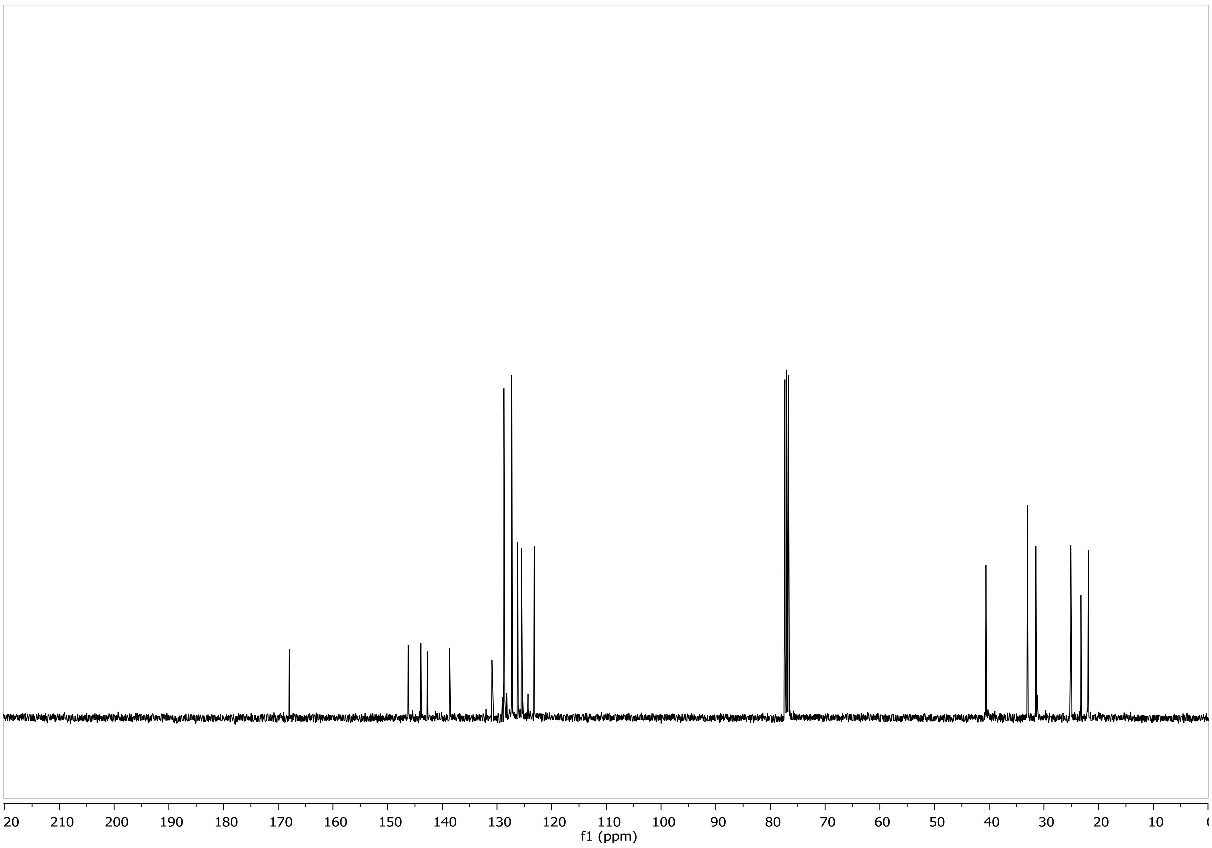
20 210 200 190 180 170 160 150 140 130 120 110 100 90 80 70 60 50 40 30 20 10
f1 (ppm)

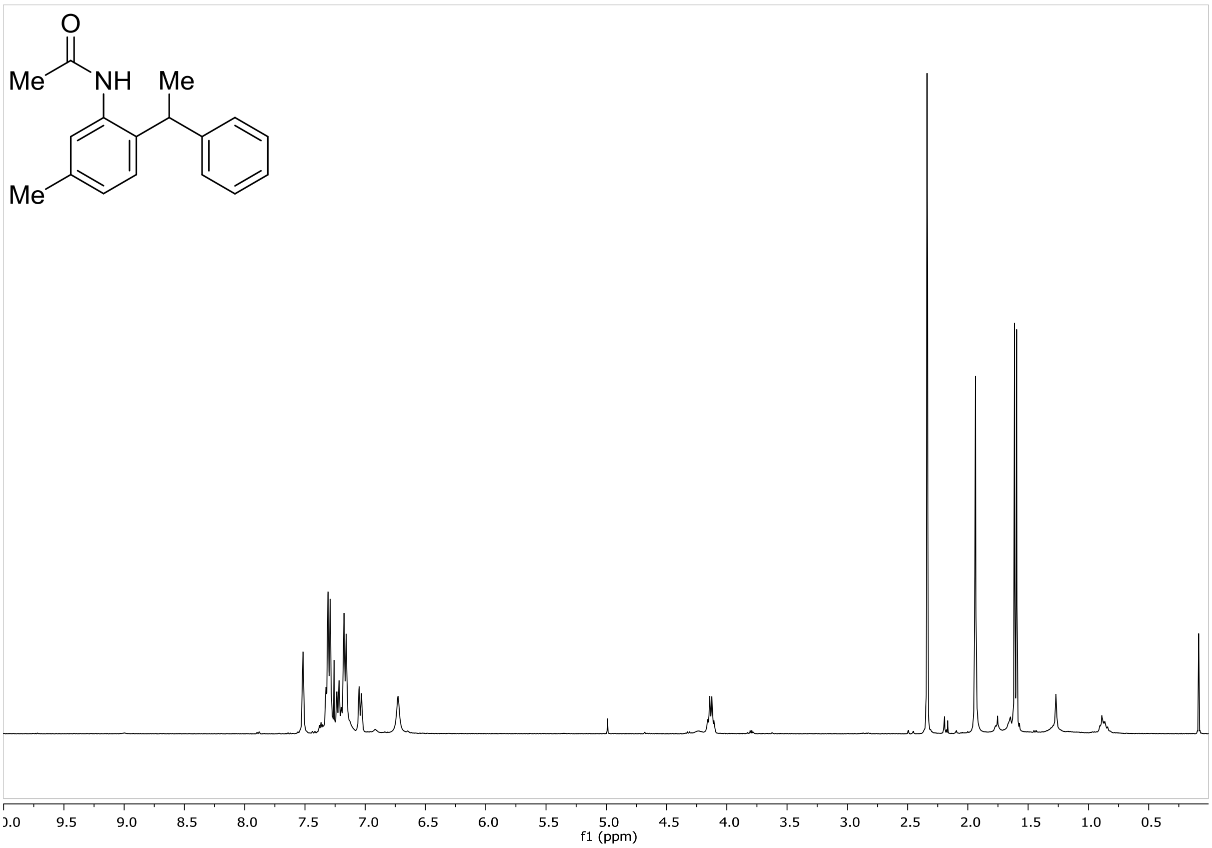
O
Me
NH
Me
Me
f1 (ppm)

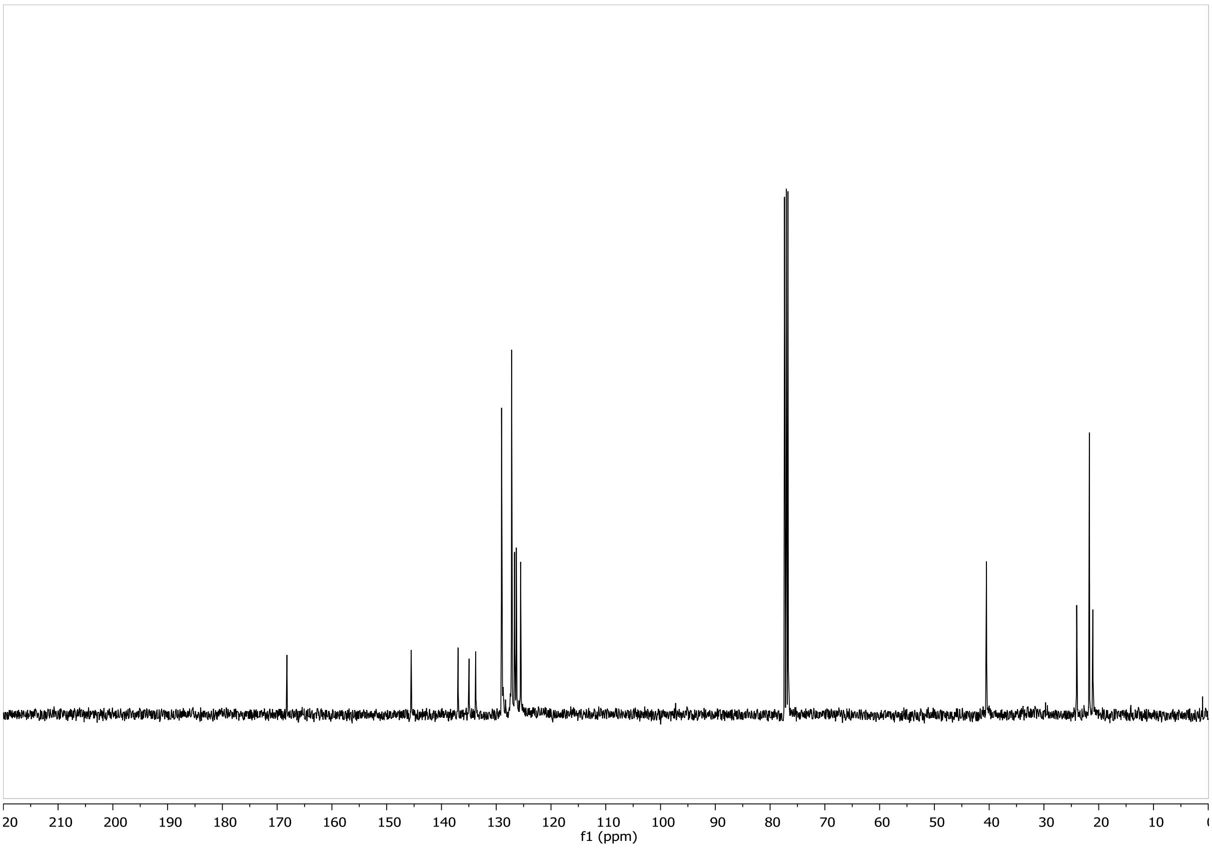
f1 (ppm)

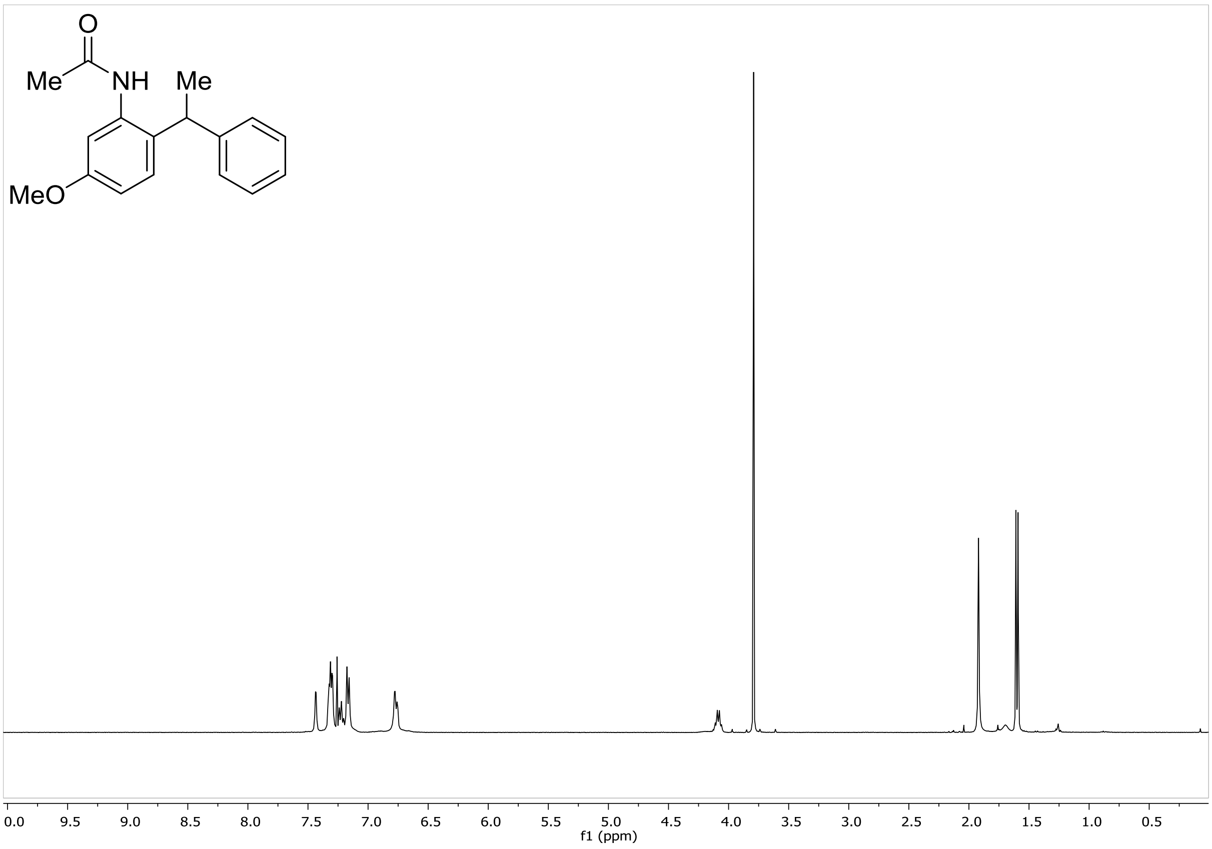
O
Me
NH
Me
MeO
0.0 9.5 9.0 8.5 8.0 7.5 7.0 6.5 6.0 5.5 5.0 4.5 4.0 3.5 3.0 2.5 2.0 1.5 1.0 0.5
f1 (ppm)

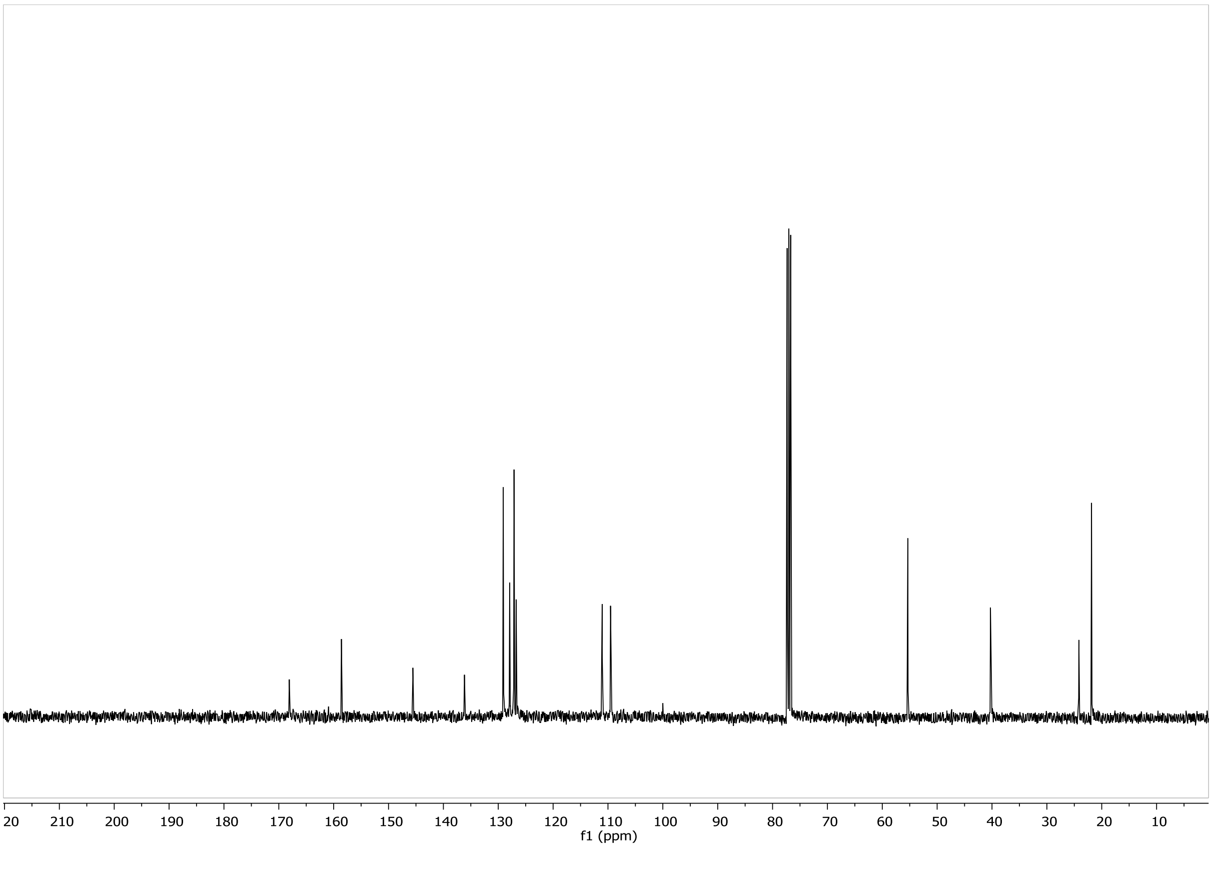
20 210 200 190 180 170 160 150 140 130 120 110 100 90 80 70 60 50 40 30 20 10
f1 (ppm)

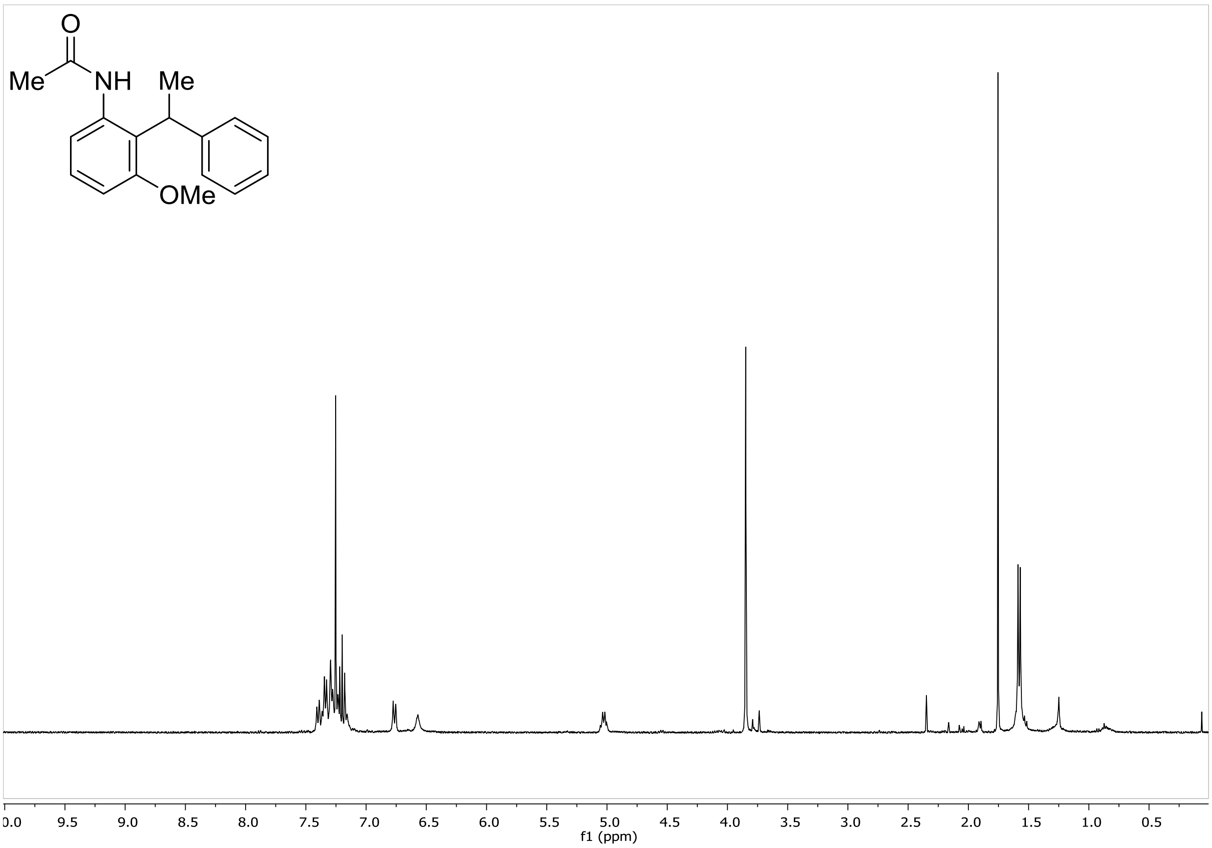
O
Me
NH
Me
OMe
f1 (ppm)

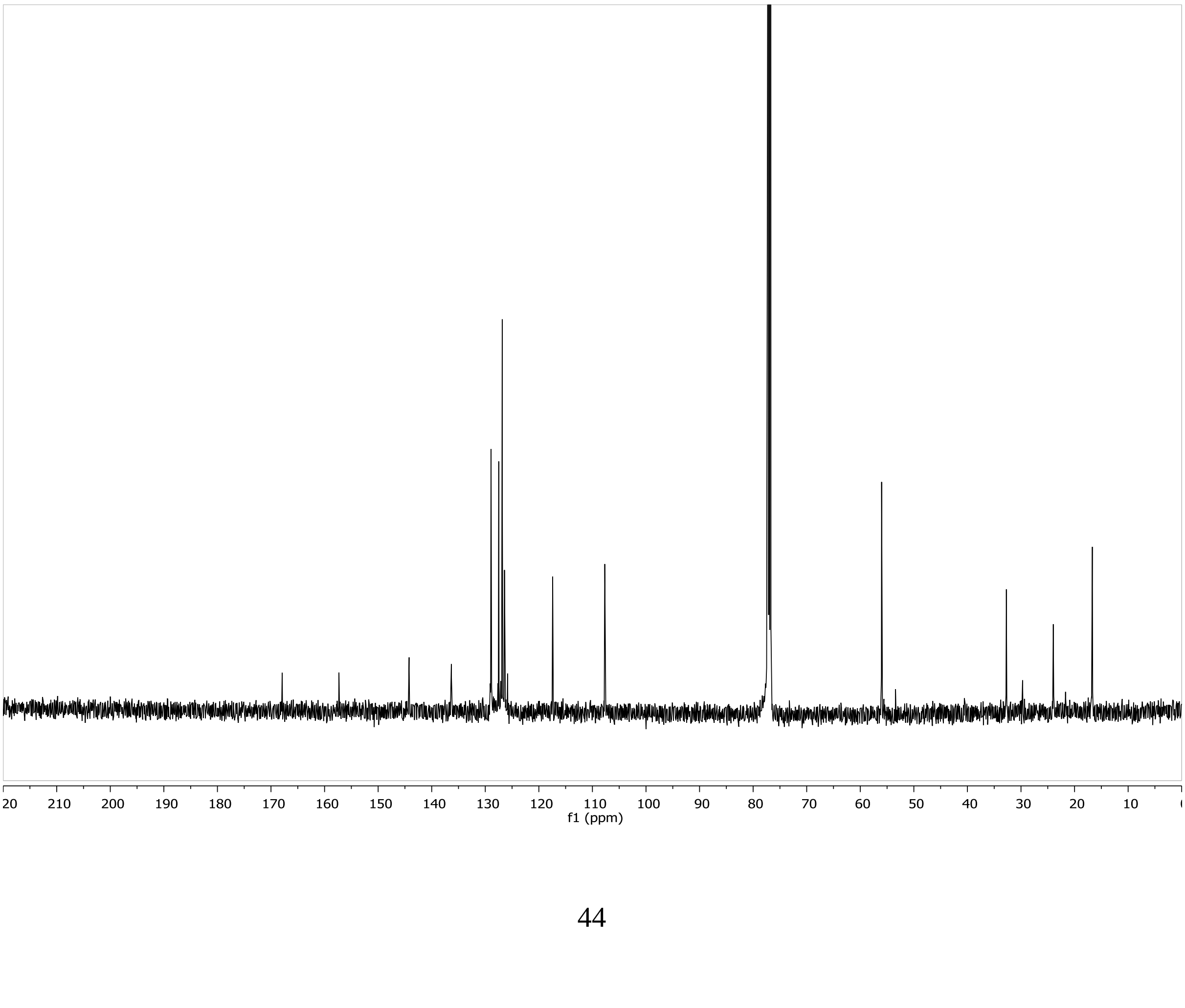
f1 (ppm)

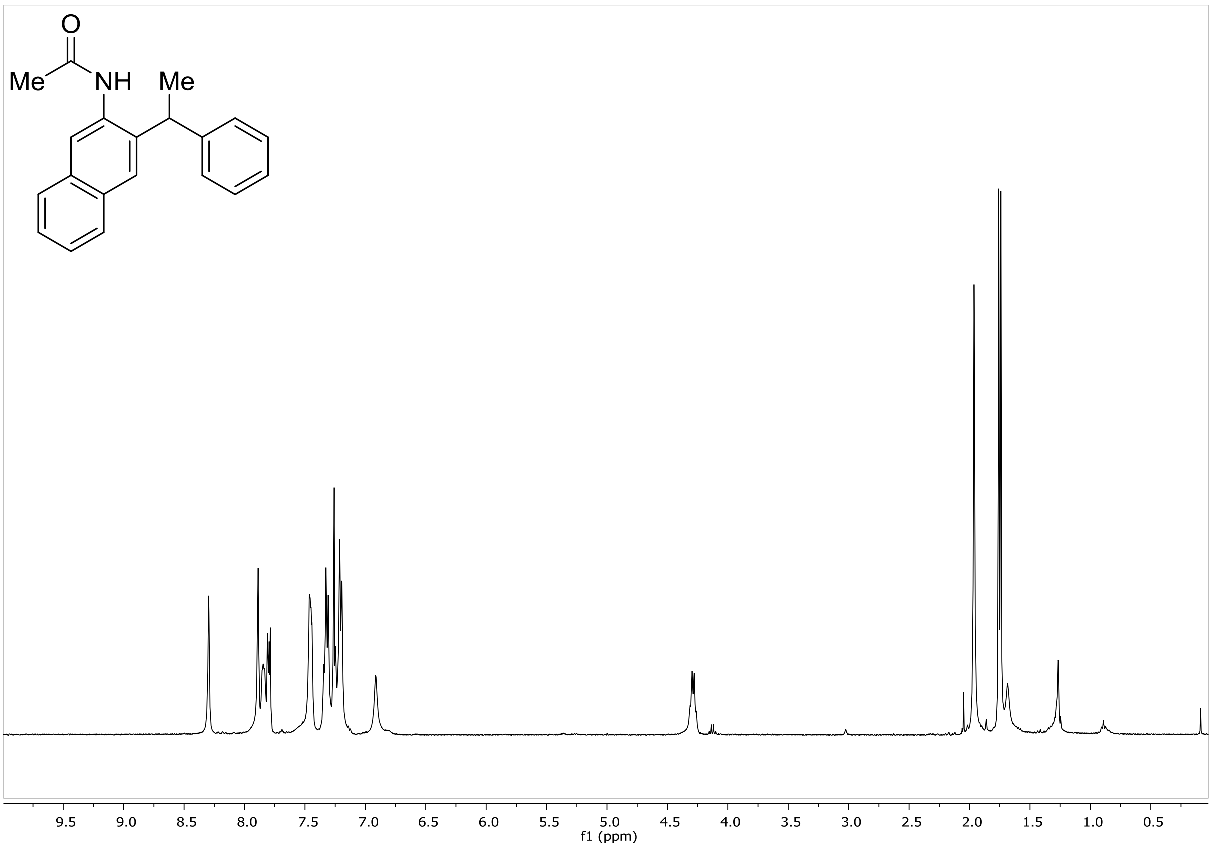

Me
NH
Me
O
9.5
9.0
8.5
8.0
7.5
7.0
6.5
6.0
5.5
5.0
4.5
4.0
3.5
3.0
2.5
2.0
1.5
1.0
0.5
f1 (ppm)

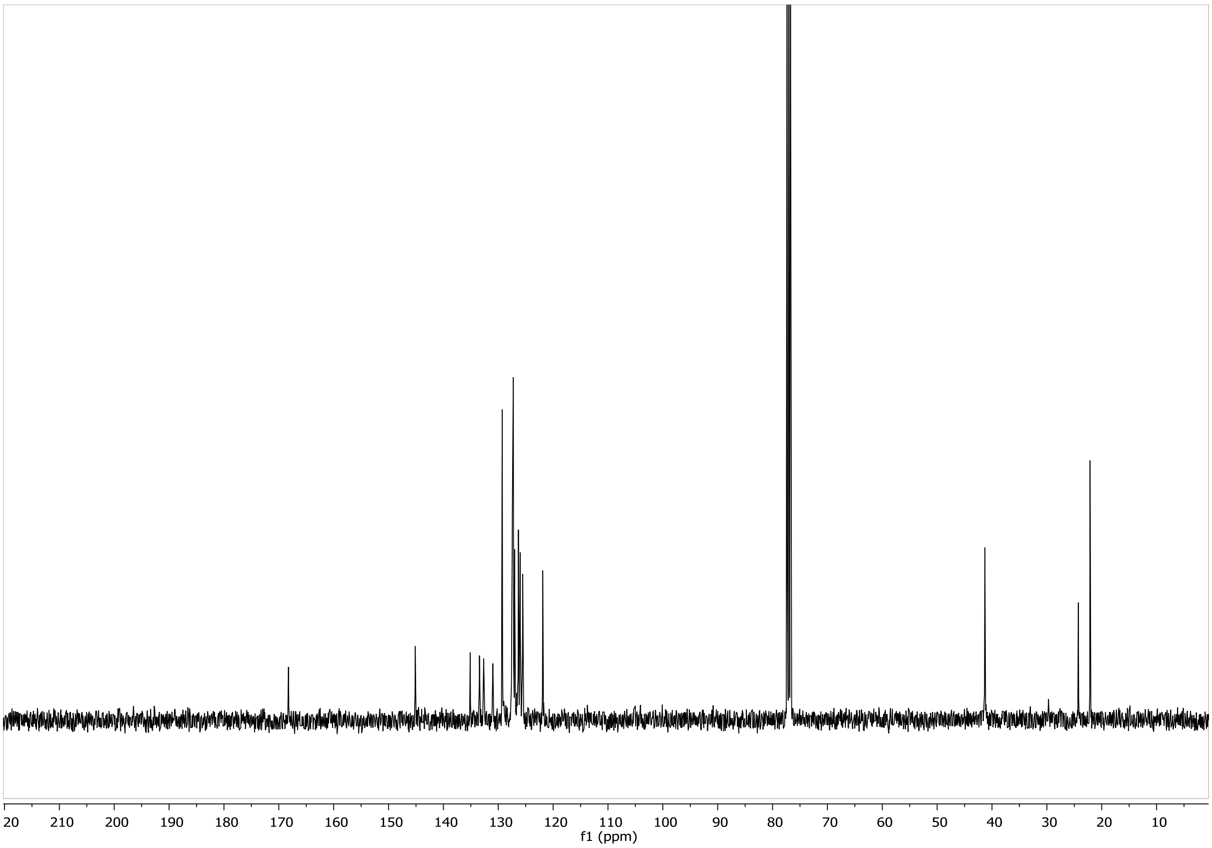

20
210
200
190
180
170
160
150
140
130
120
110
100
90
80
70
60
50
40
30
20
10
f1 (ppm)

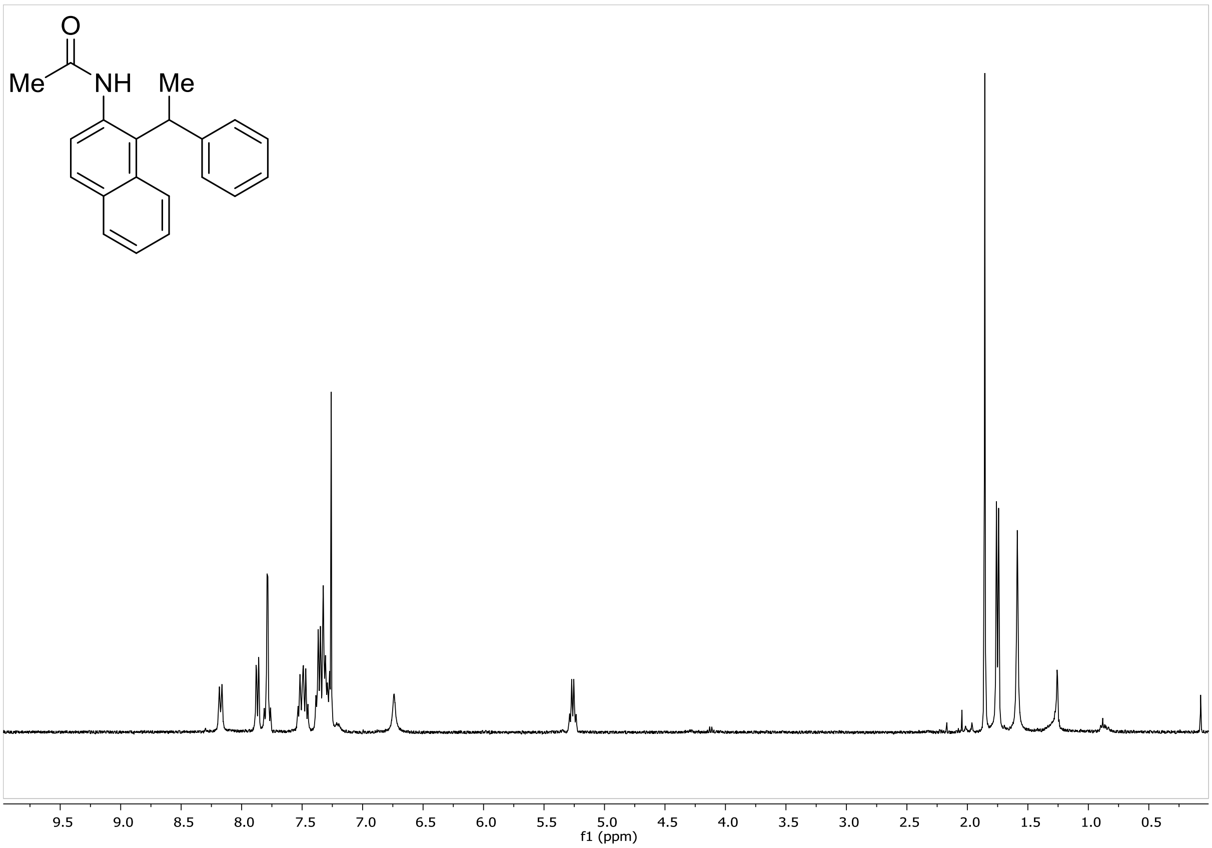
O
Me
NH
Me
9.5 9.0 8.5 8.0 7.5 7.0 6.5 6.0 5.5 5.0 4.5 4.0 3.5 3.0 2.5 2.0 1.5 1.0 0.5
f1 (ppm)

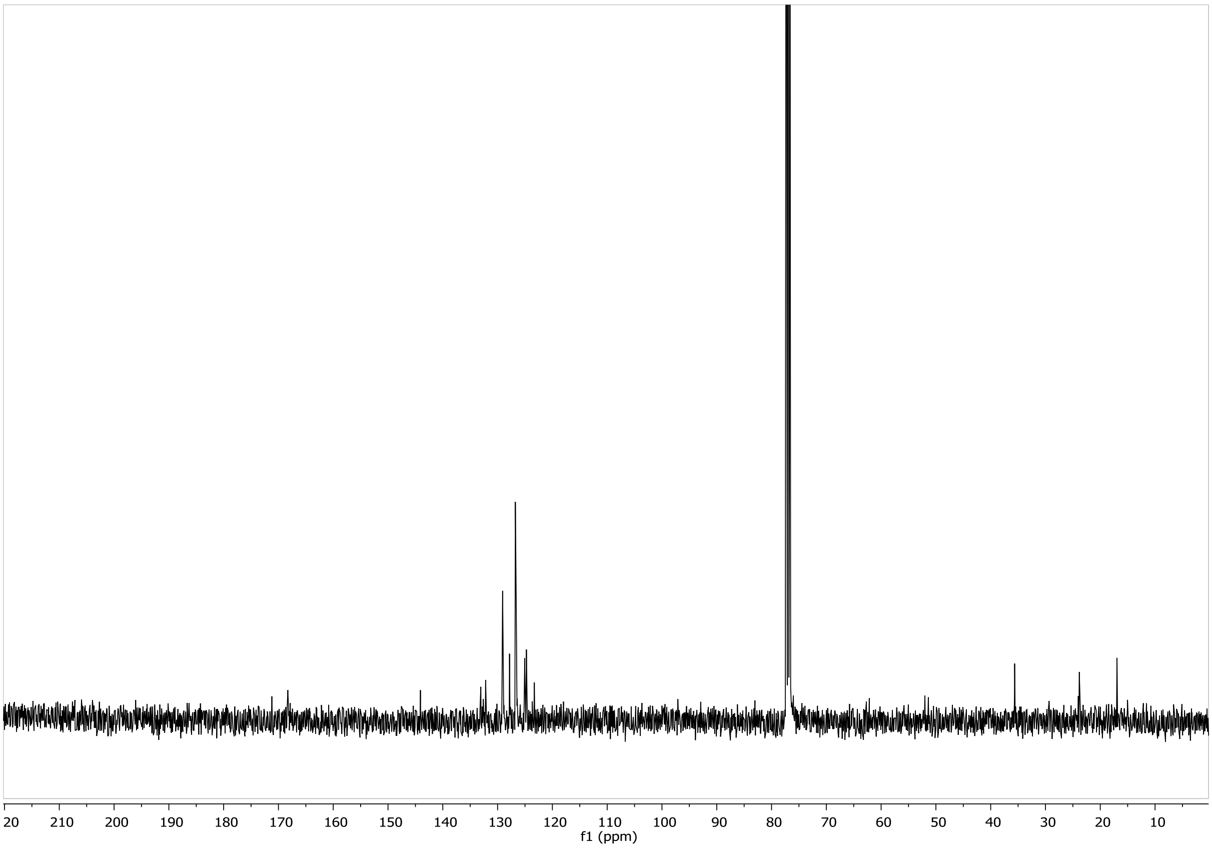
20 210 200 190 180 170 160 150 140 130 120 110 100 90 80 70 60 50 40 30 20 10
f1 (ppm)

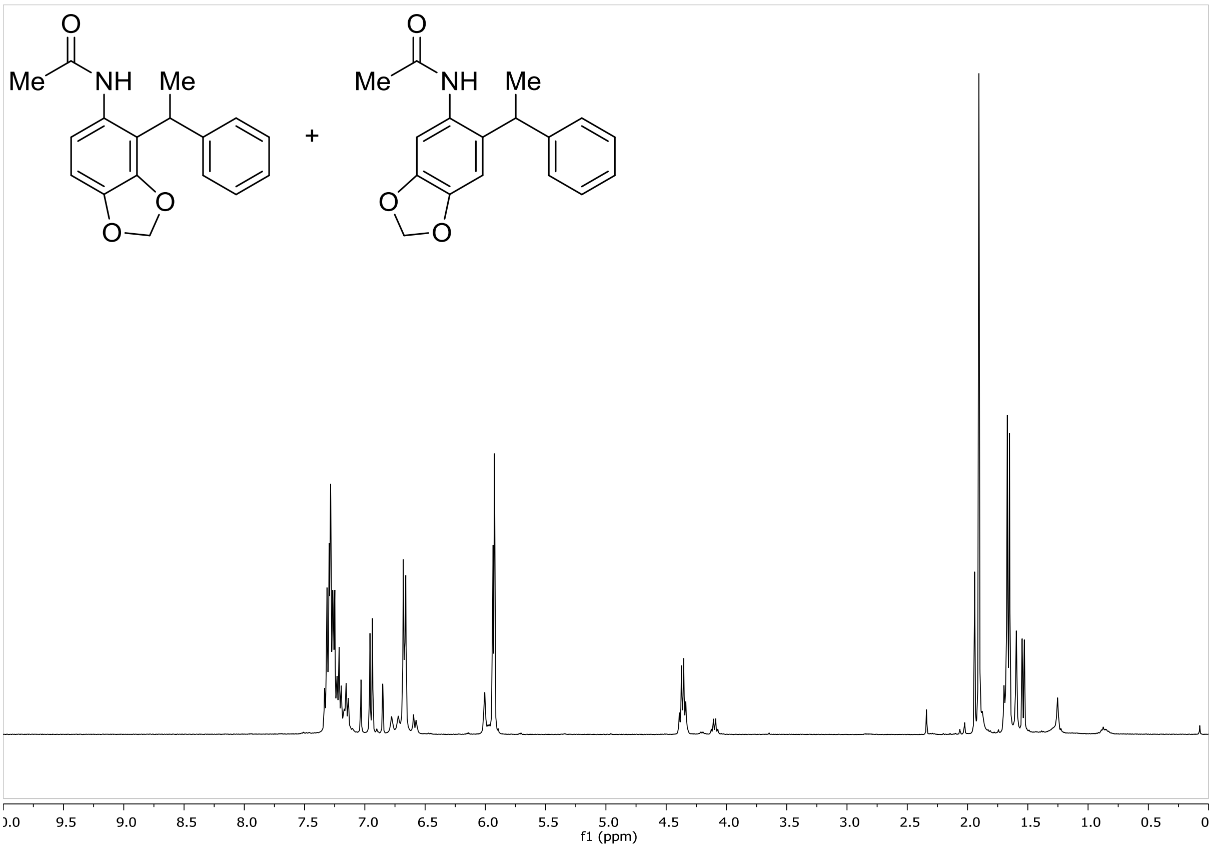
O
Me NH Me
O
O
+
O
Me NH Me
O
O
9.5 9.0 8.5 8.0 7.5 7.0 6.5 6.0 5.5 5.0 4.5 4.0 3.5 3.0 2.5 2.0 1.5 1.0 0.5 0
f1 (ppm)

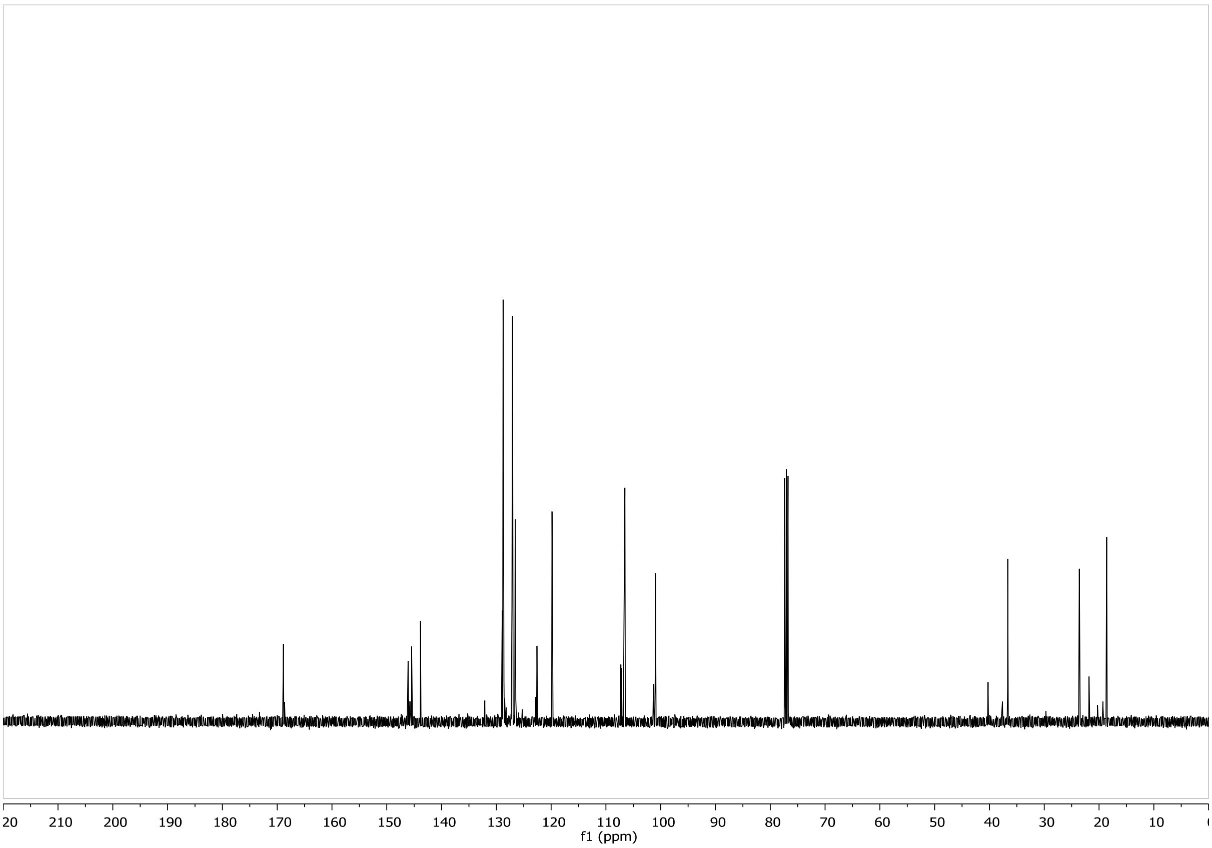
210 200 190 180 170 160 150 140 130 120 110 100 90 80 70 60 50 40 30 20 10
f1 (ppm)

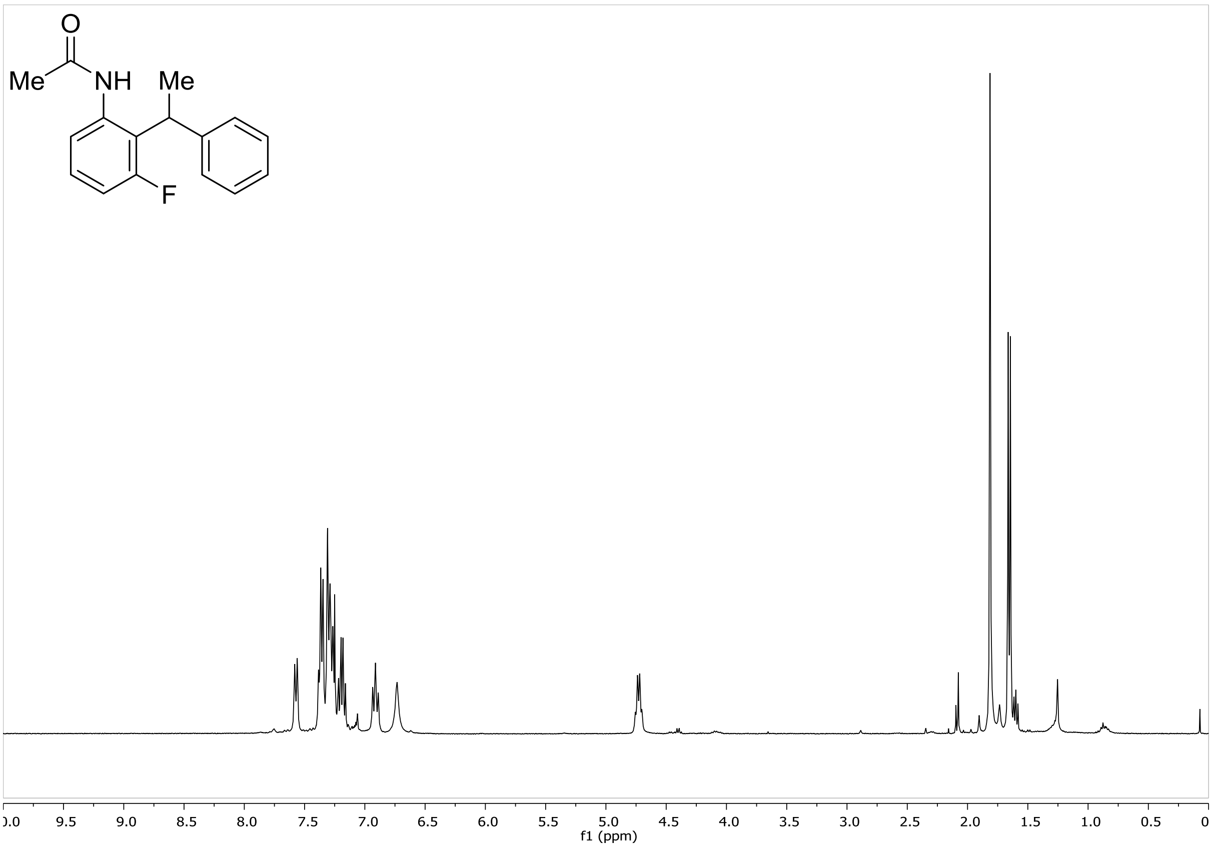
O
Me
NH
Me
F
f1 (ppm)

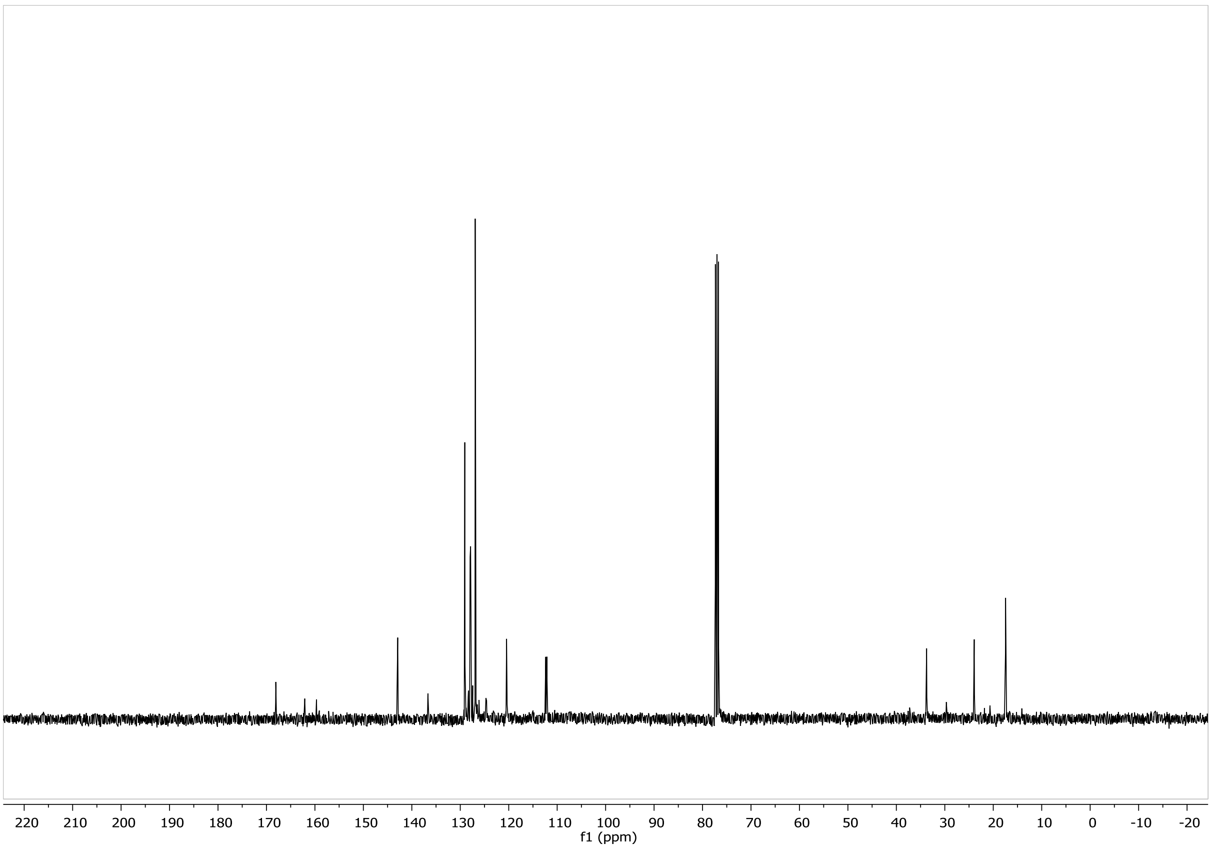
f1 (ppm)

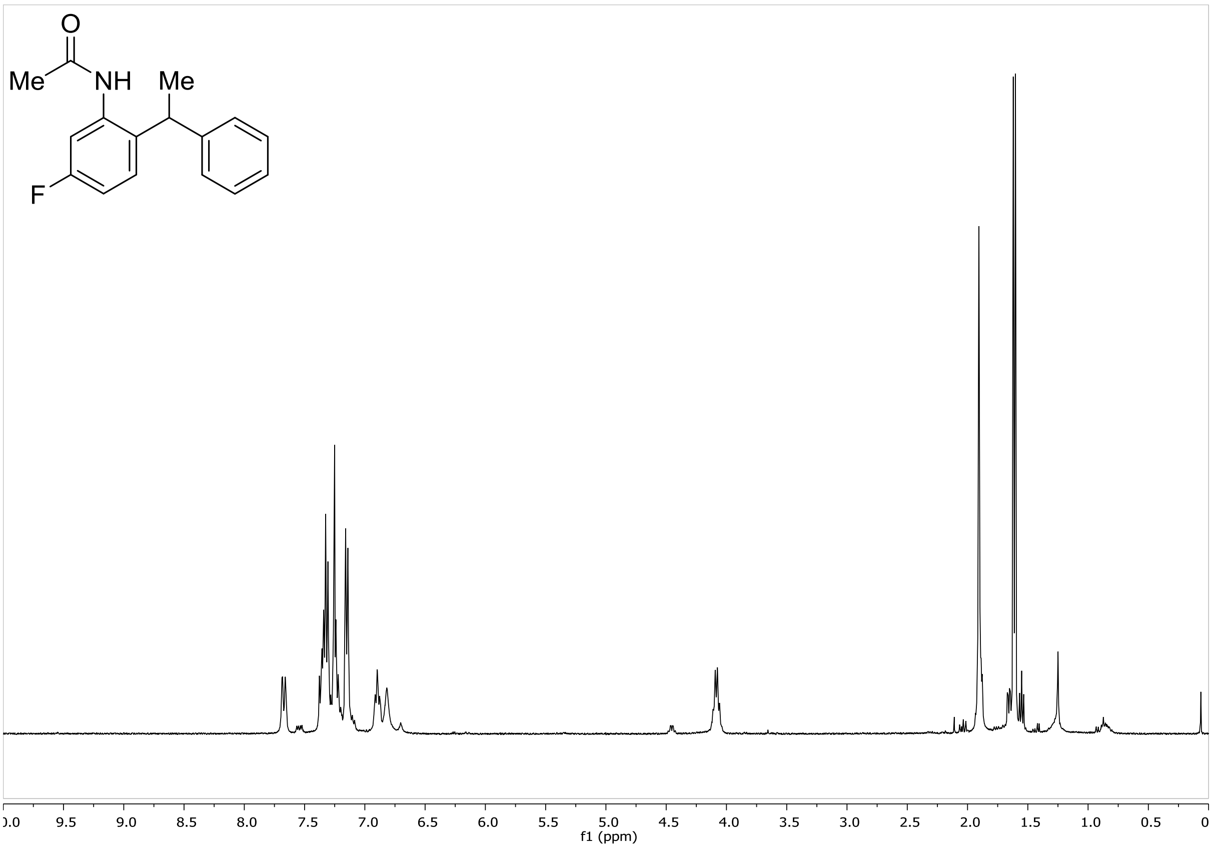
O
NH
Me
Me
F
f1 (ppm)

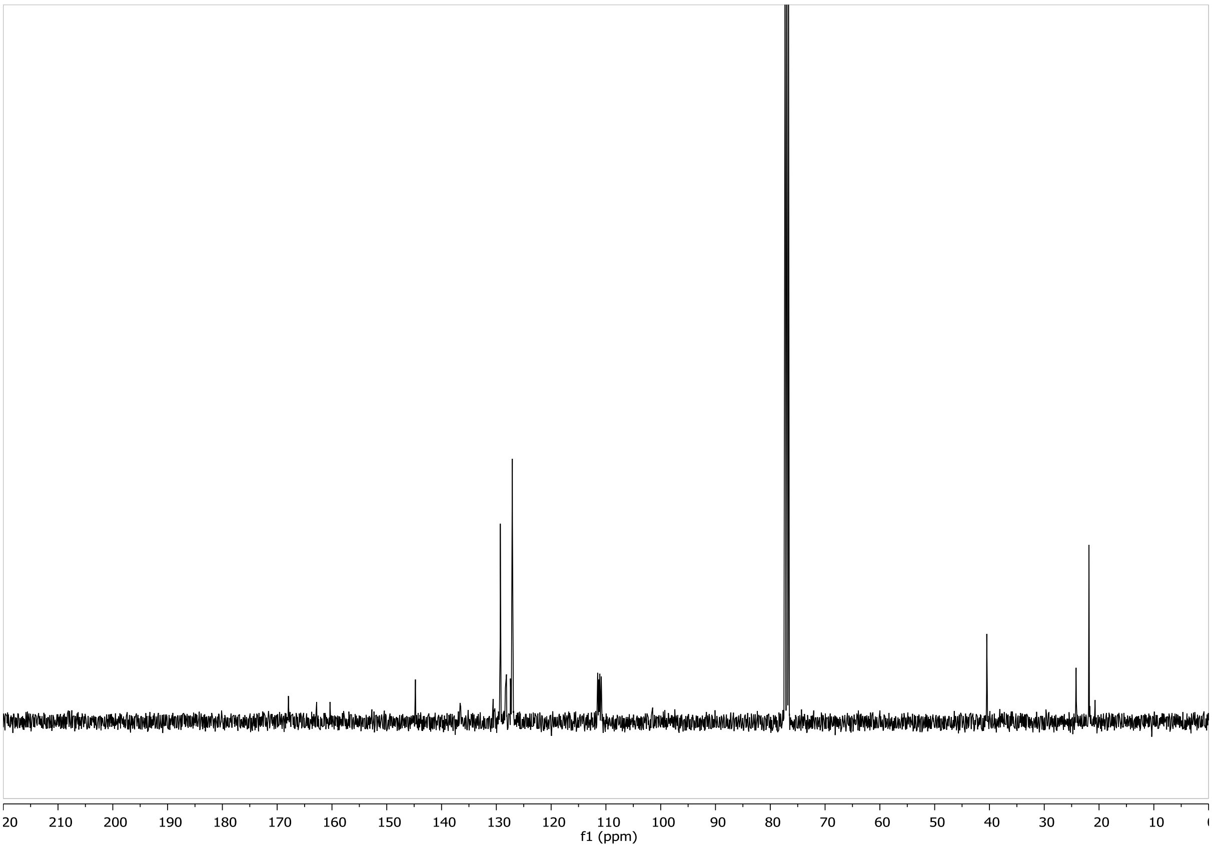
f1 (ppm)

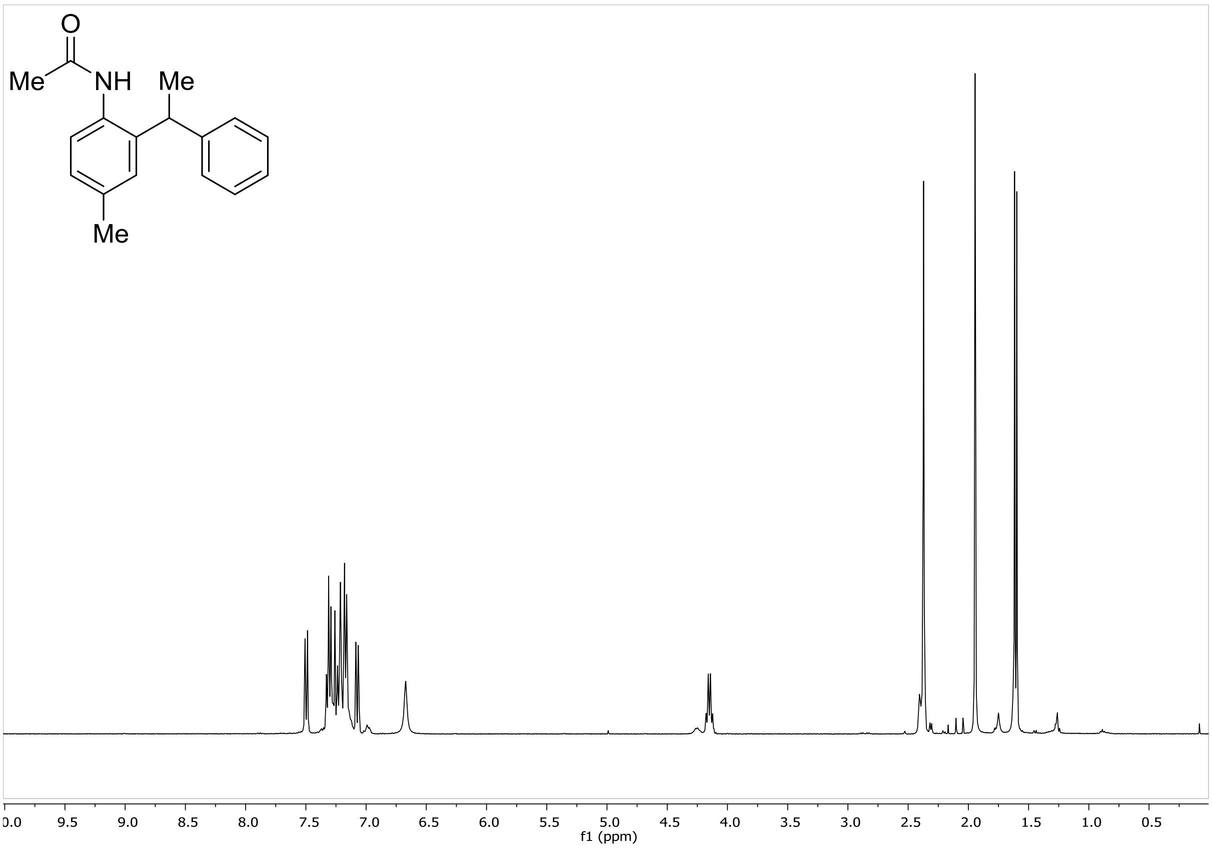
O
Me
NH
Me
Me
f1 (ppm)

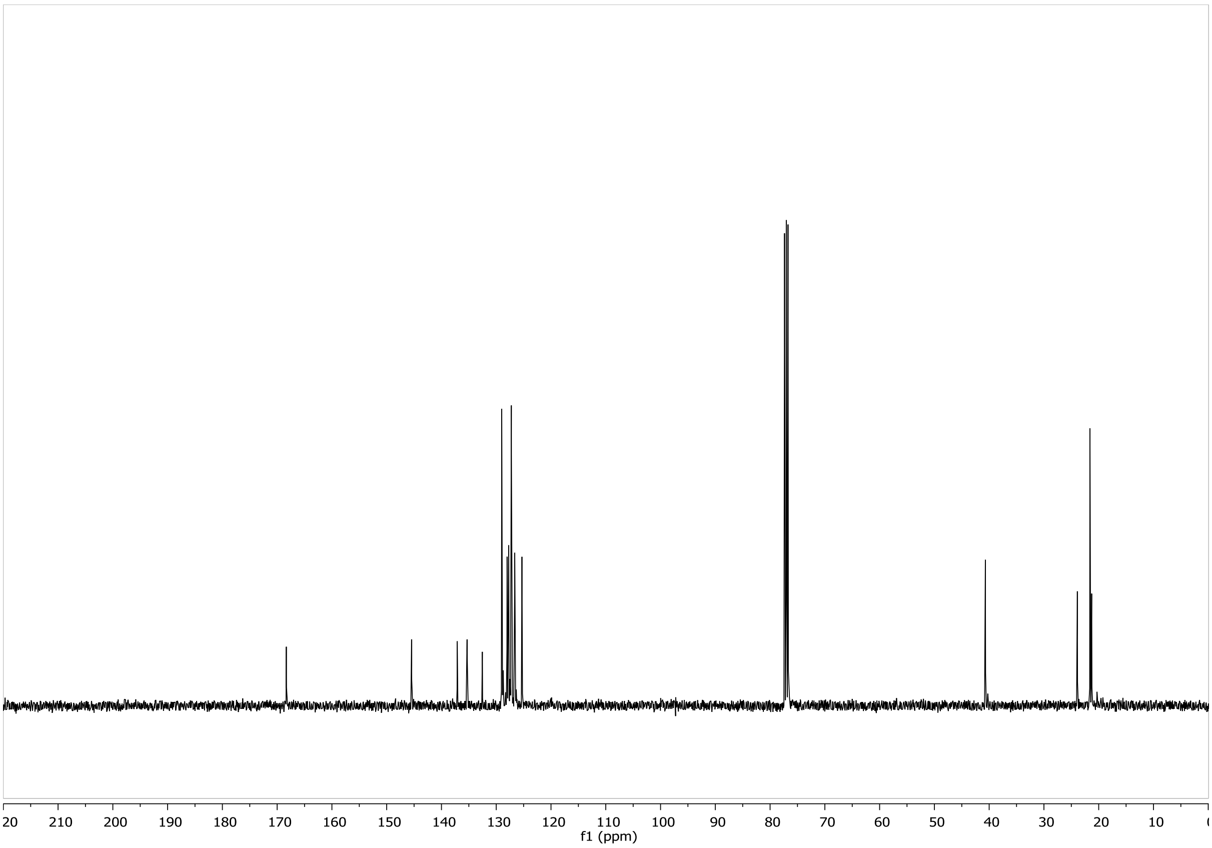
f1 (ppm)

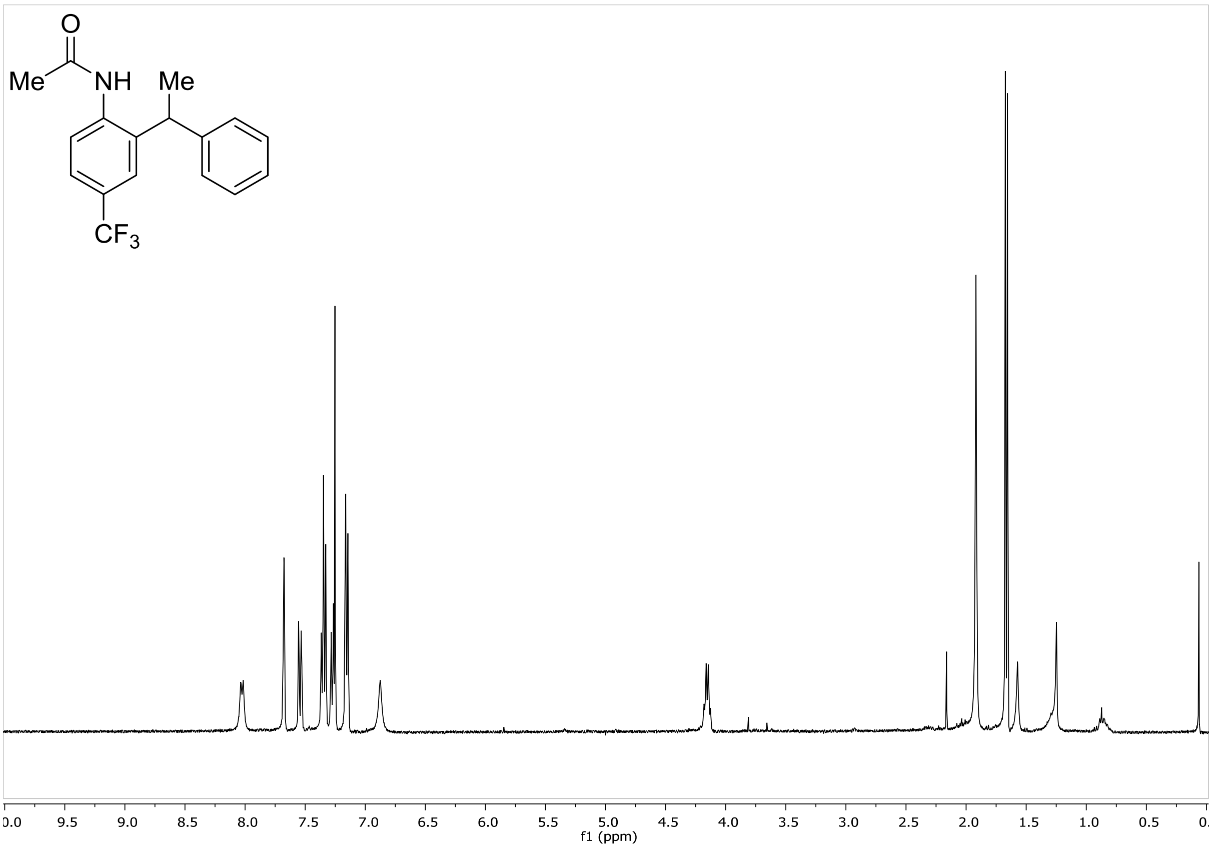
O
Me
NH
Me
CF3
f1 (ppm)

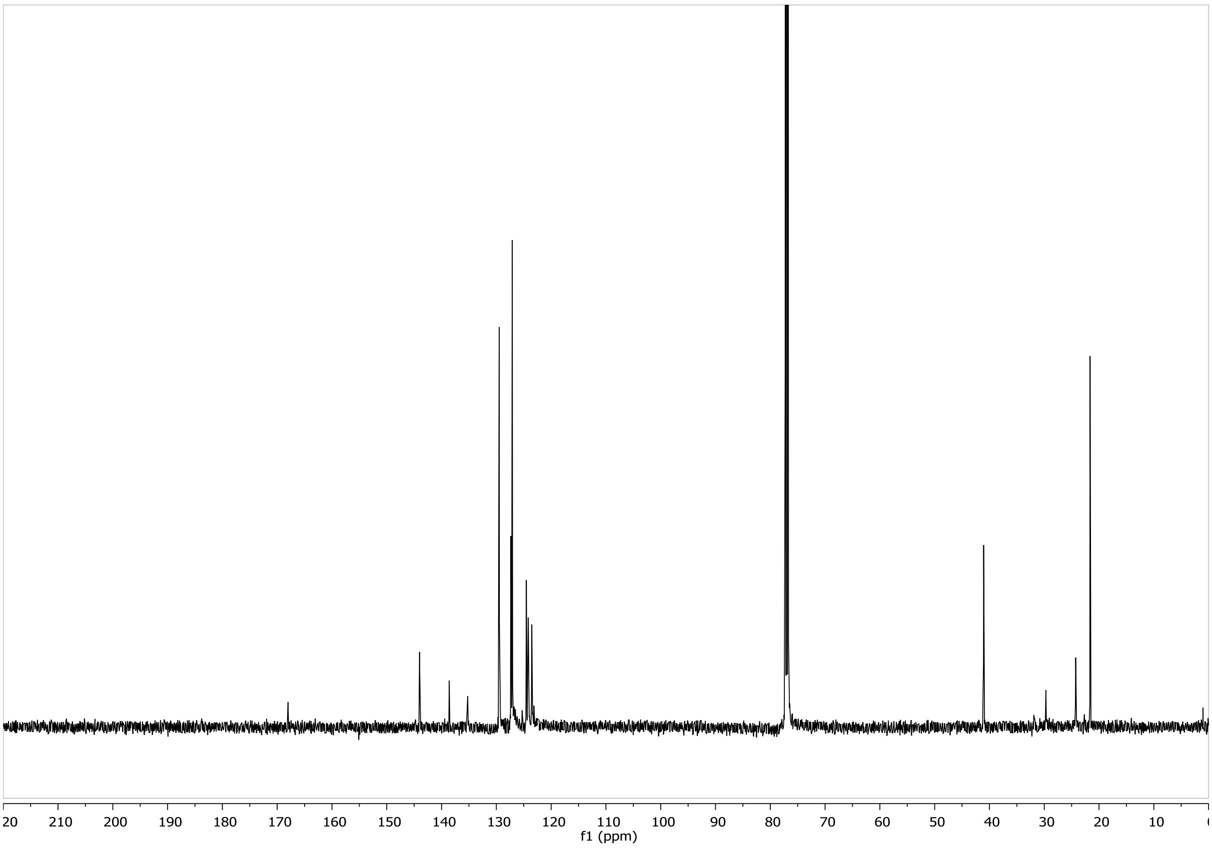
f1 (ppm)

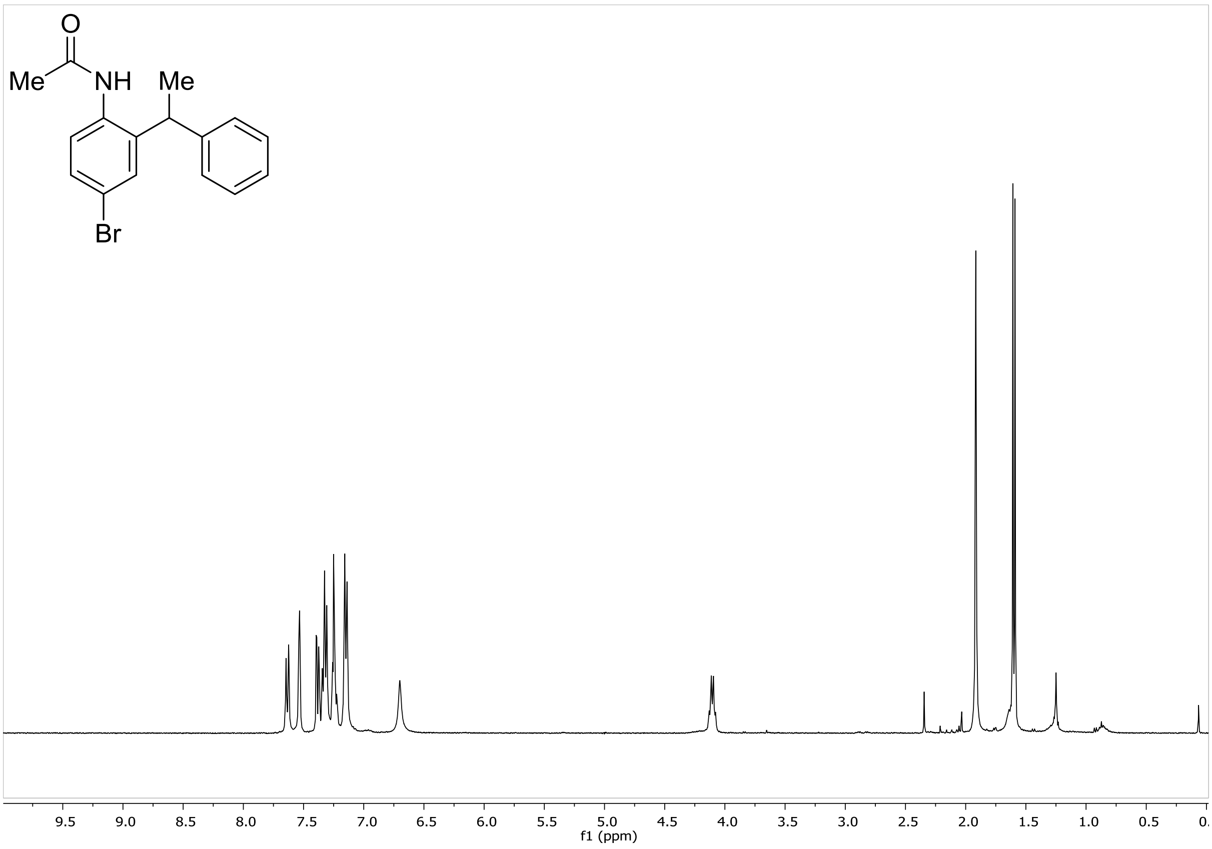
O
Me
NH
Me
Br
9.5 9.0 8.5 8.0 7.5 7.0 6.5 6.0 5.5 5.0 4.5 4.0 3.5 3.0 2.5 2.0 1.5 1.0 0.5
f1 (ppm)

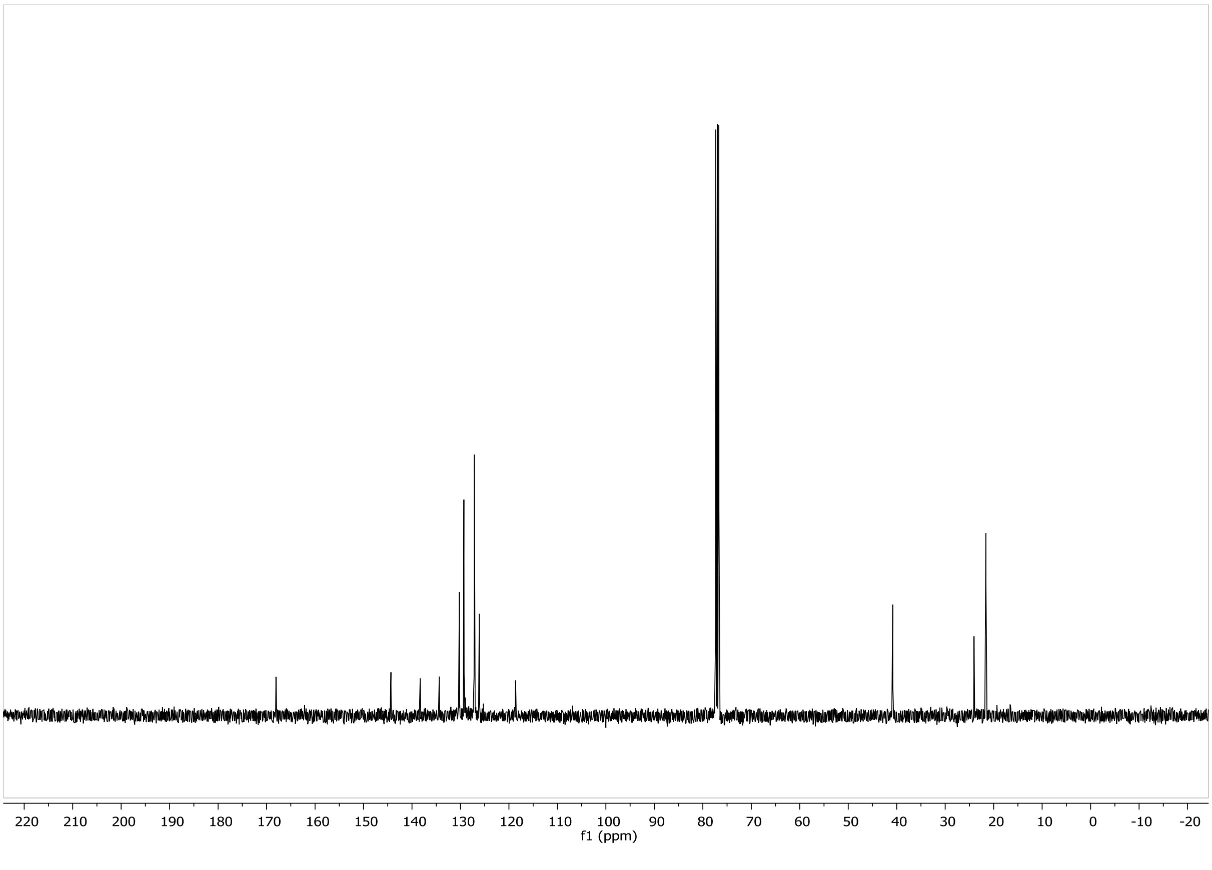
220 210 200 190 180 170 160 150 140 130 120 110 100 90 80 70 60 50 40 30 20 10 0 -10 -20
f1 (ppm)

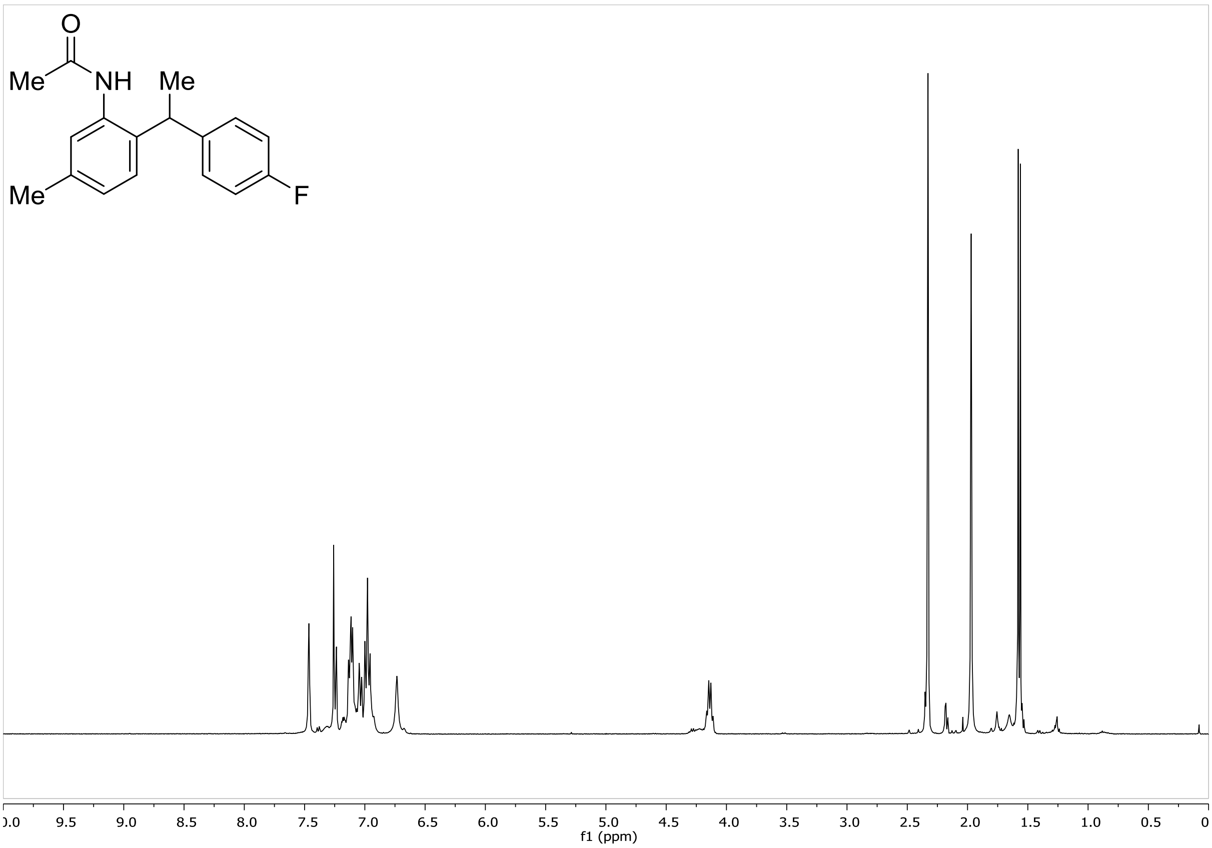
O
NH
Me
Me
Me
F
9.5
9.0
8.5
8.0
7.5
7.0
6.5
6.0
5.5
5.0
4.5
4.0
3.5
3.0
2.5
2.0
1.5
1.0
0.5
f1 (ppm)

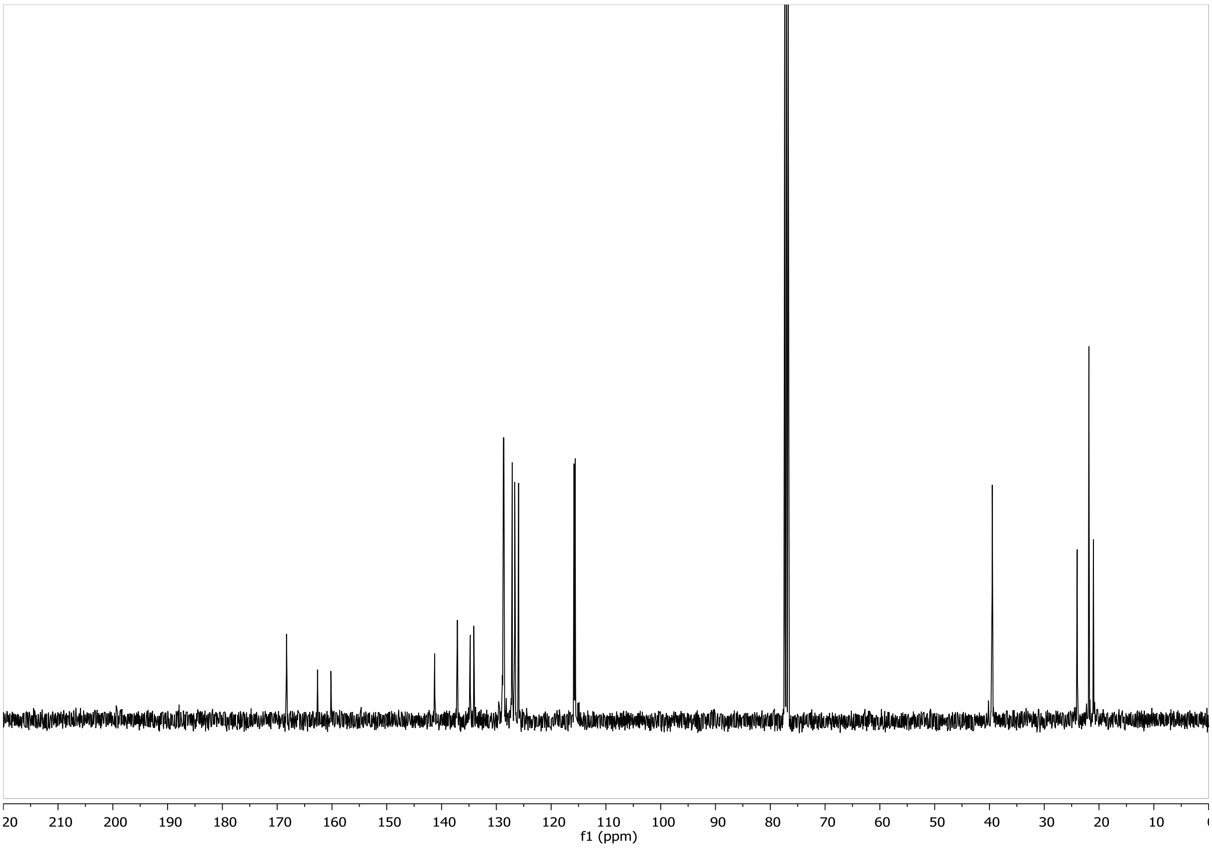
210
200
190
180
170
160
150
140
130
120
110
100
90
80
70
60
50
40
30
20
10
f1 (ppm)

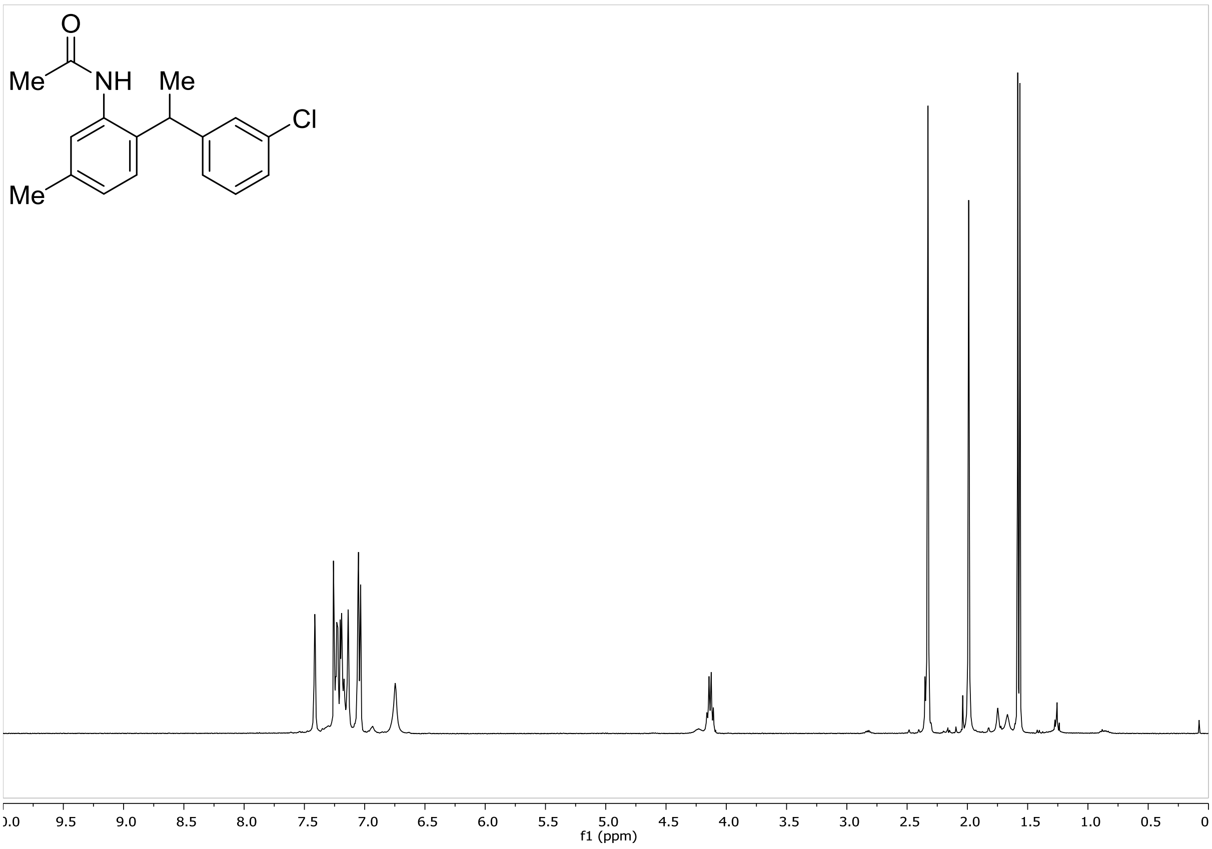
O
Me
NH
Me
Cl
Me
f1 (ppm)

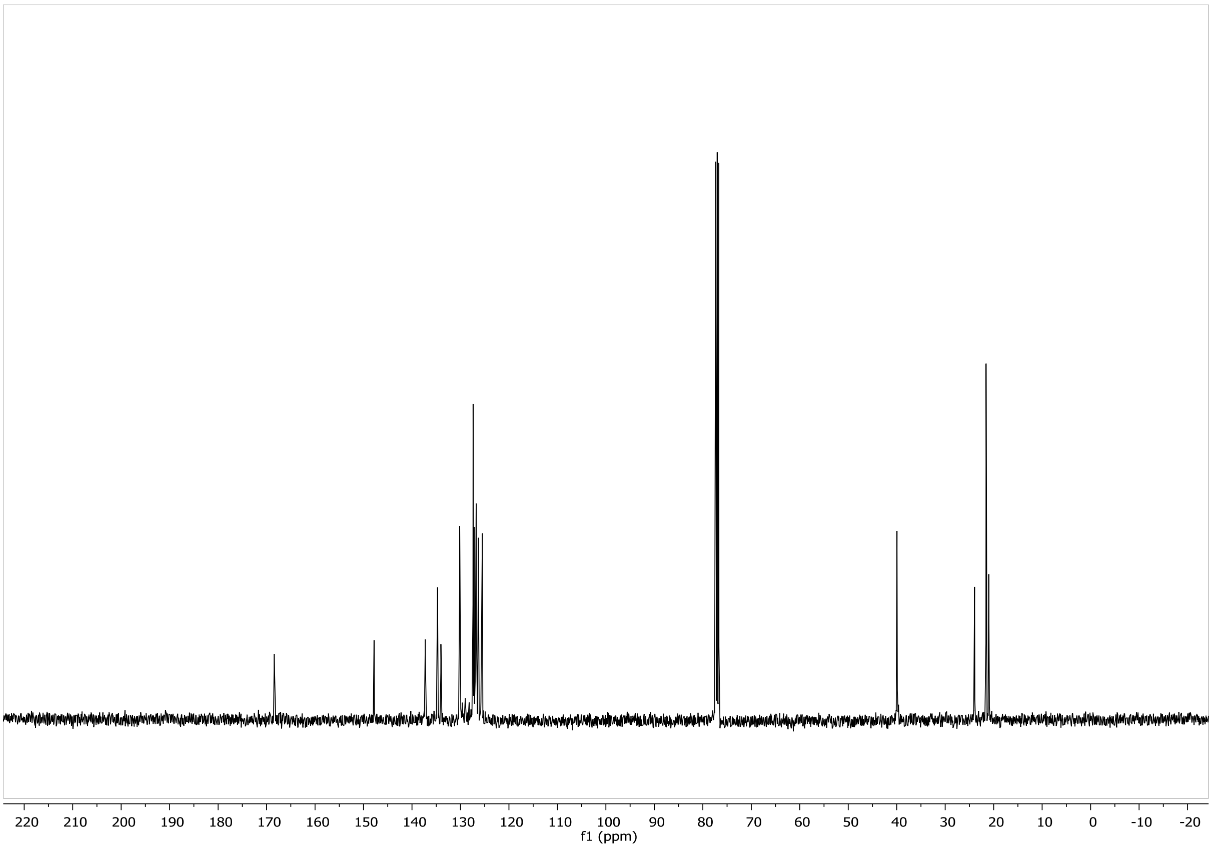
f1 (ppm)

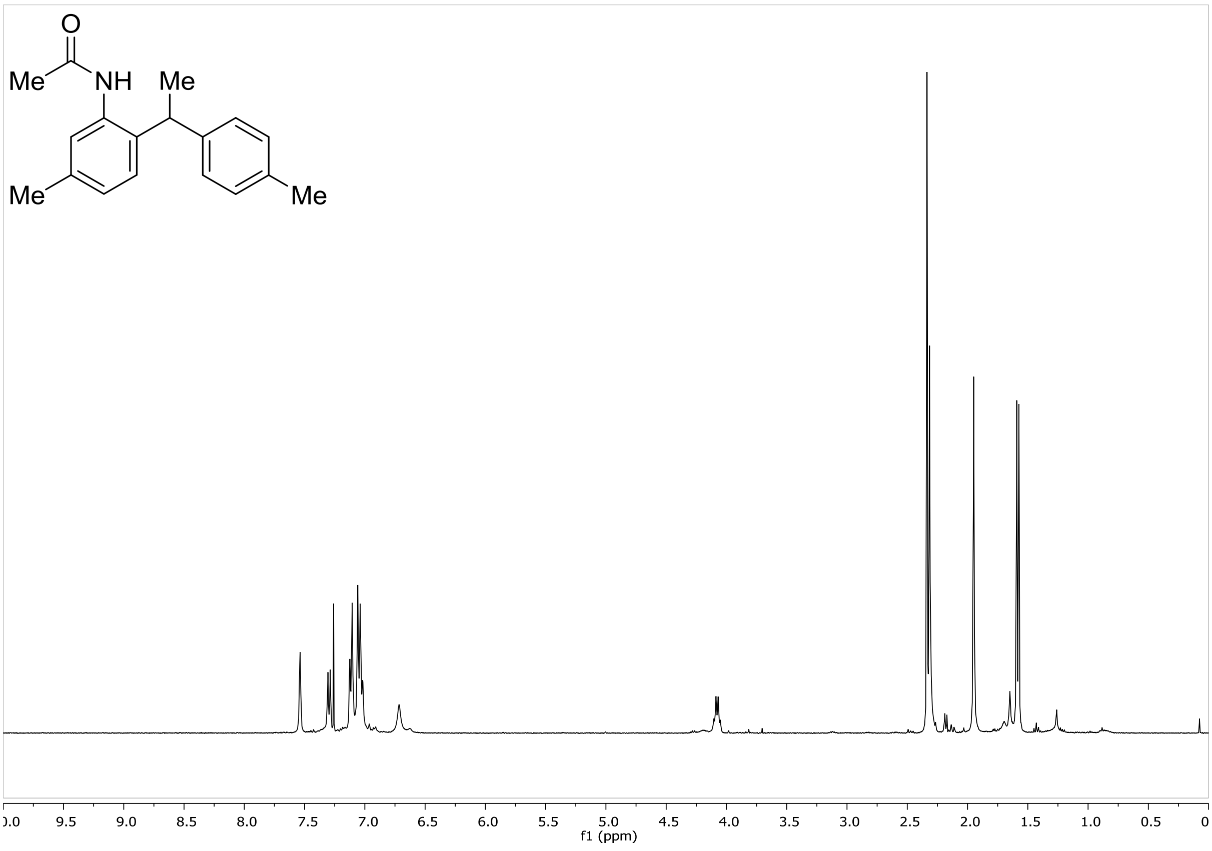
O
Me
NH
Me
Me
Me
f1 (ppm)

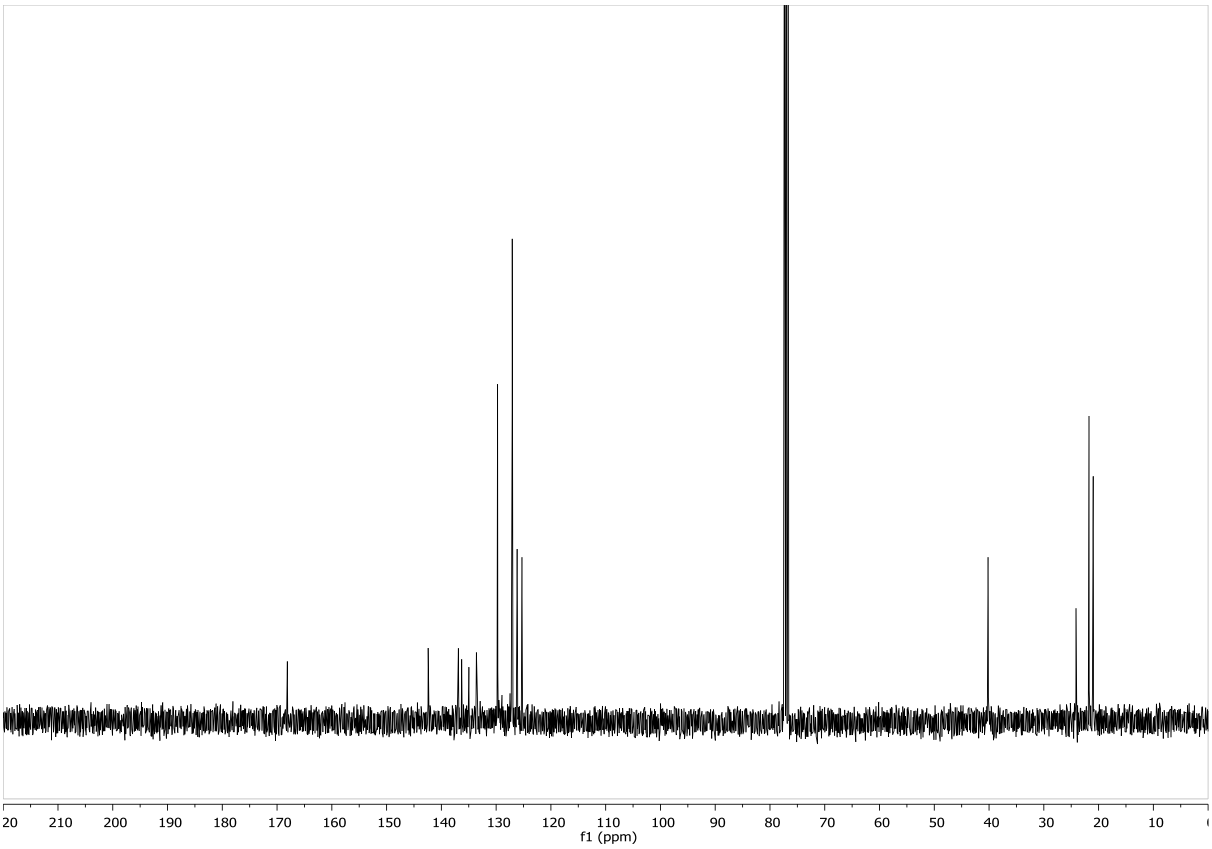
f1 (ppm)

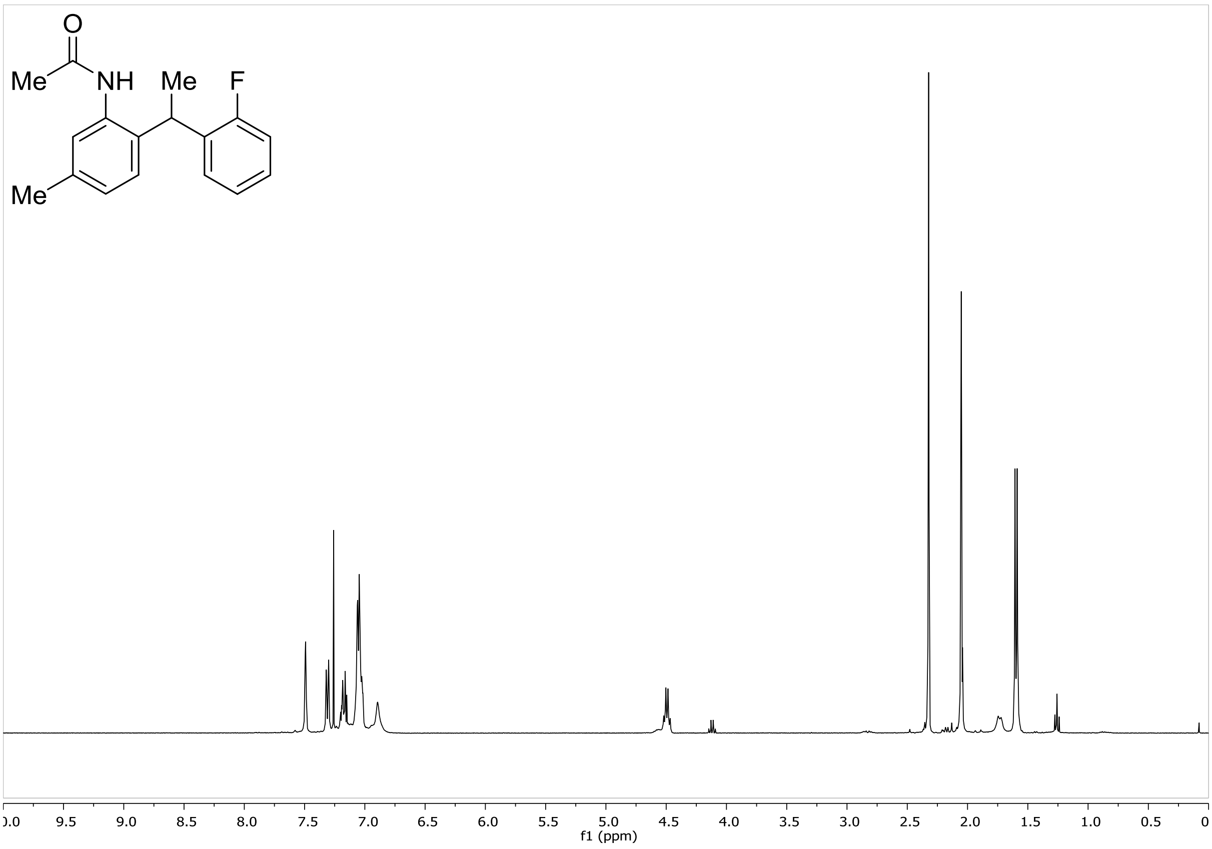
O
Me NH Me F
Me
f1 (ppm)

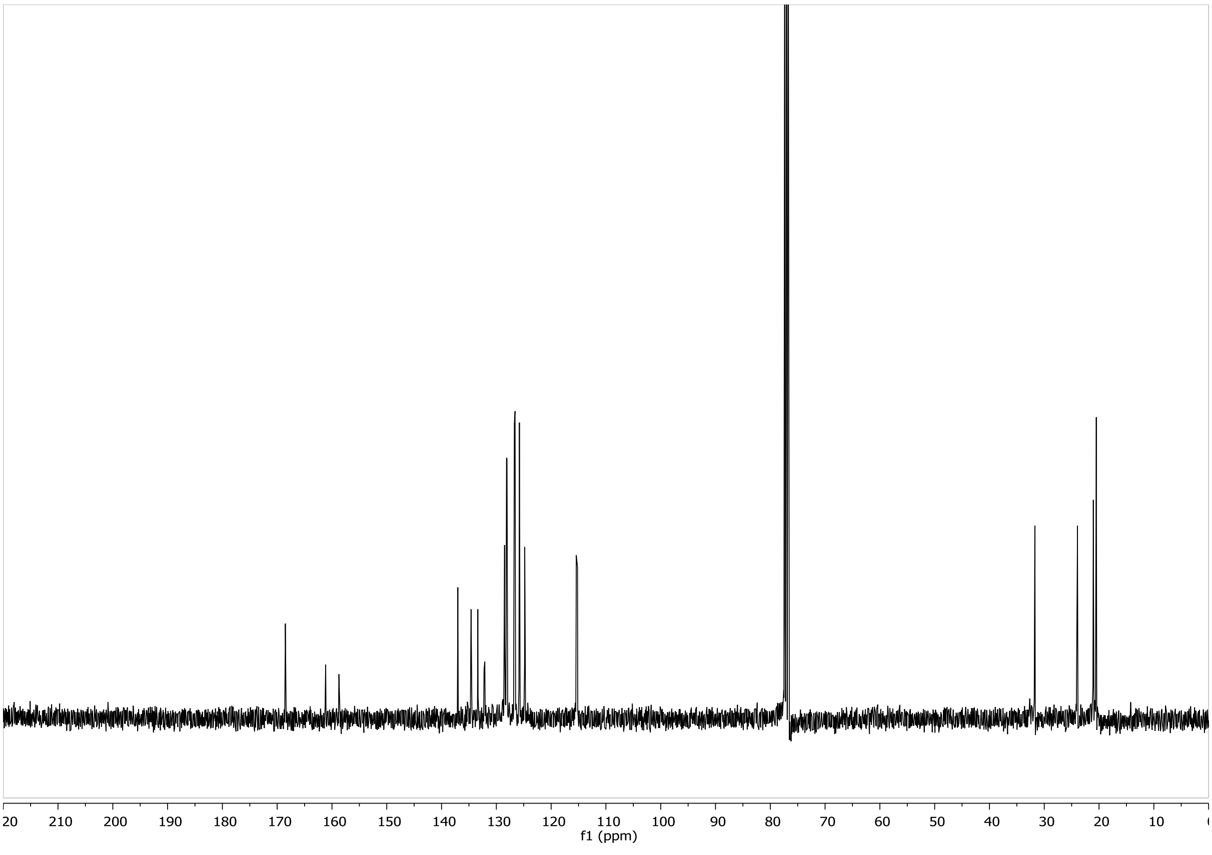
f1 (ppm)

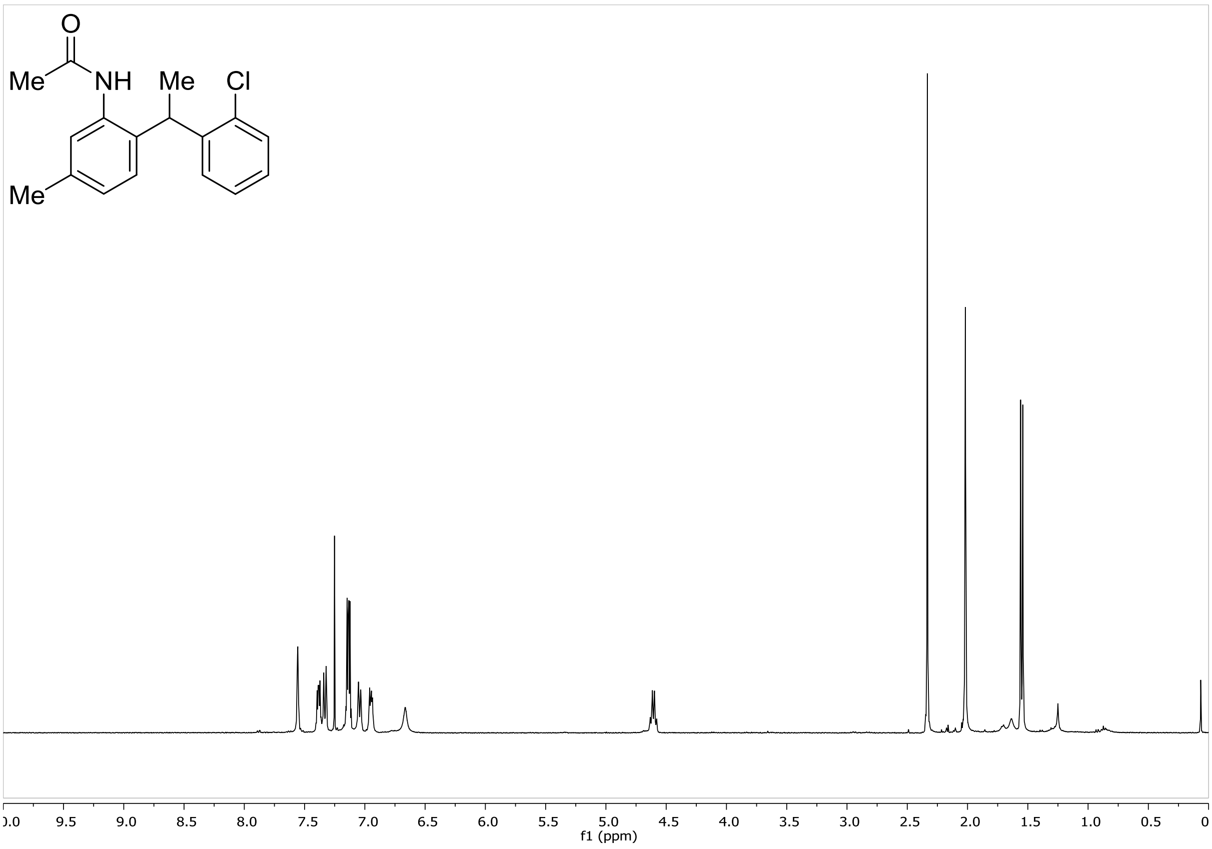
O
Me
NH
Me
Cl
Me
f1 (ppm)

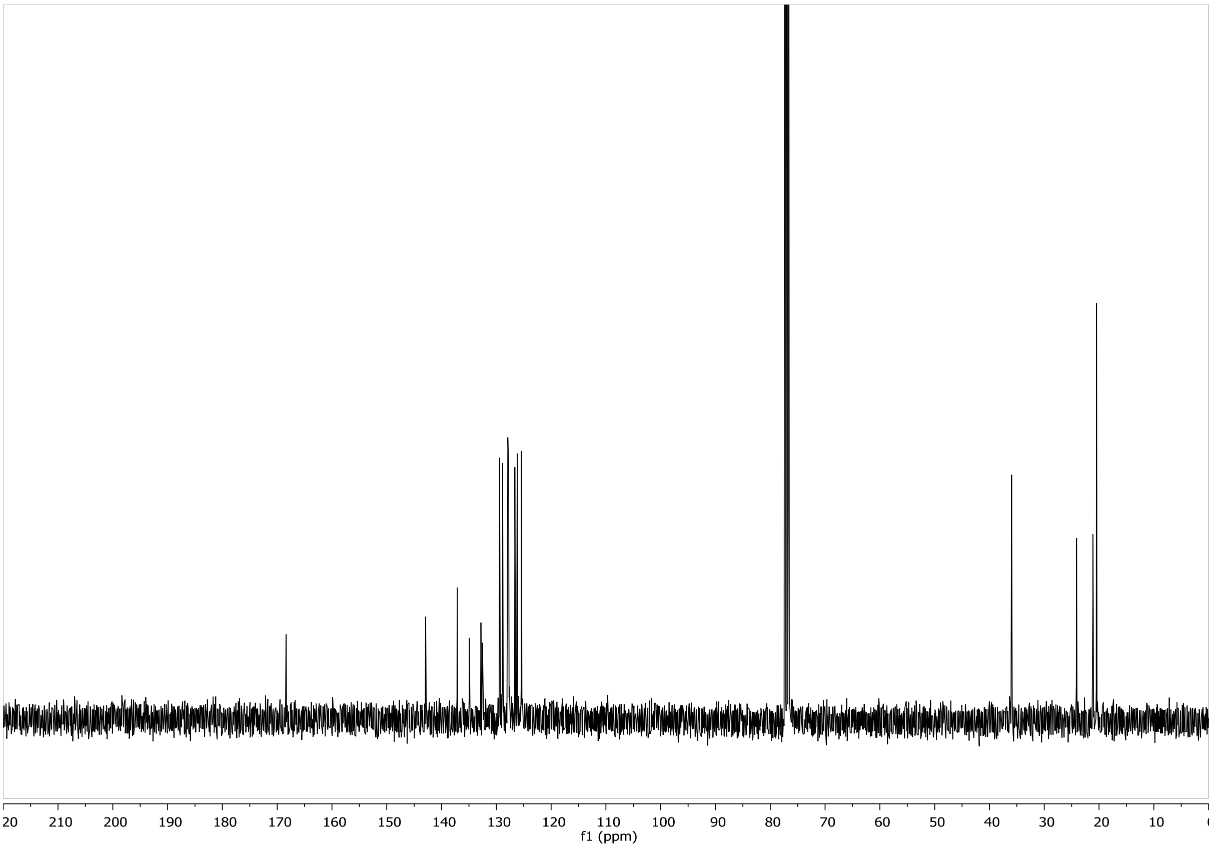
f1 (ppm)

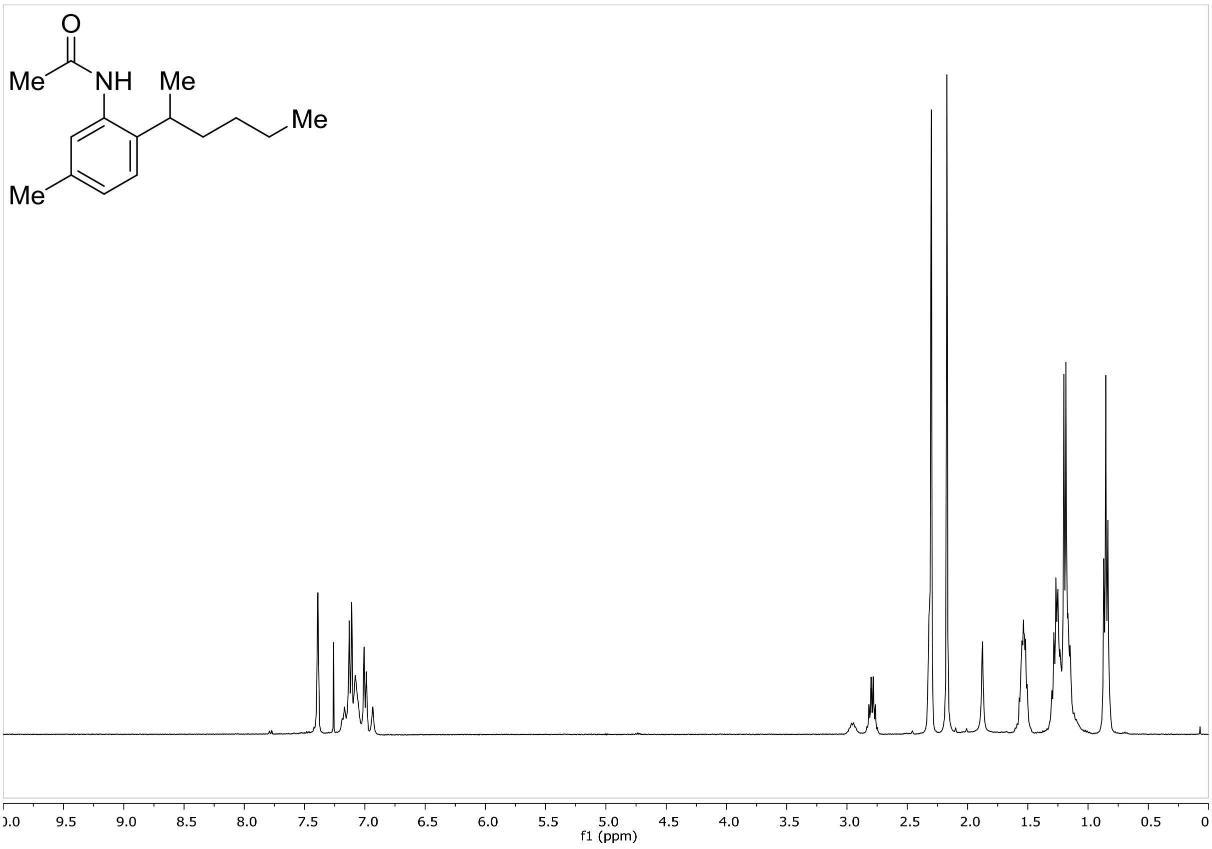
O
Me
NH
Me
Me
Me
10.0 9.5 9.0 8.5 8.0 7.5 7.0 6.5 6.0 5.5 5.0 4.5 4.0 3.5 3.0 2.5 2.0 1.5 1.0 0.5 0
f1 (ppm)

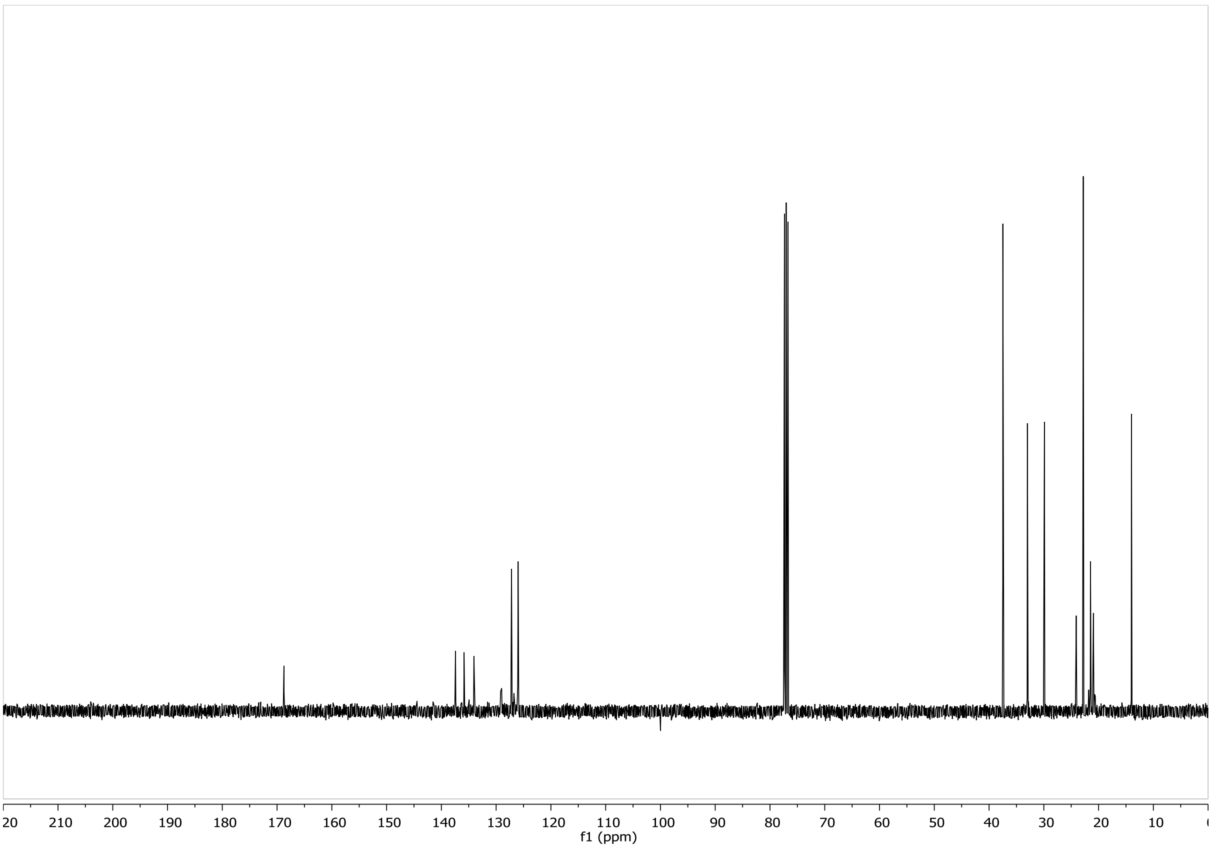
210 200 190 180 170 160 150 140 130 120 110 100 90 80 70 60 50 40 30 20 10
f1 (ppm)

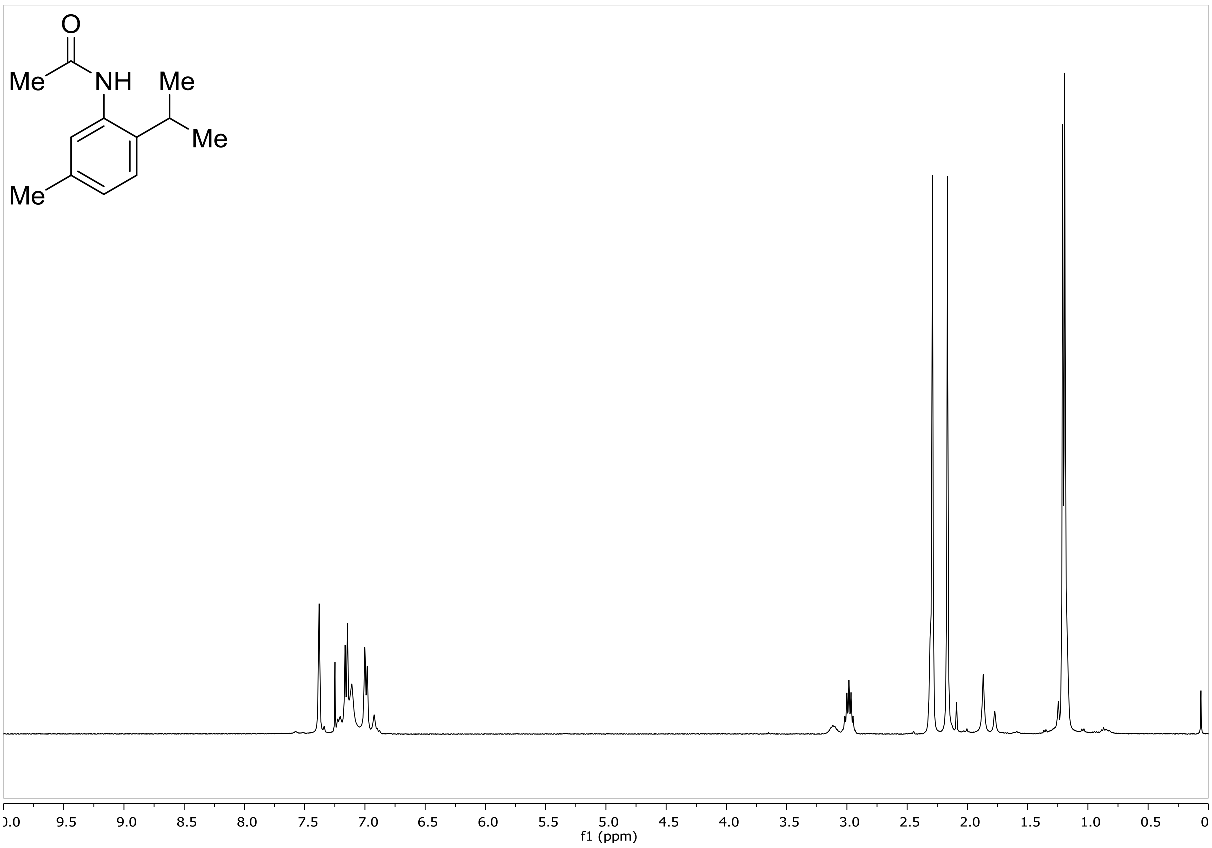
O
Me
NH
Me
Me
Me
0.0 9.5 9.0 8.5 8.0 7.5 7.0 6.5 6.0 5.5 5.0 4.5 4.0 3.5 3.0 2.5 2.0 1.5 1.0 0.5 0
f1 (ppm)

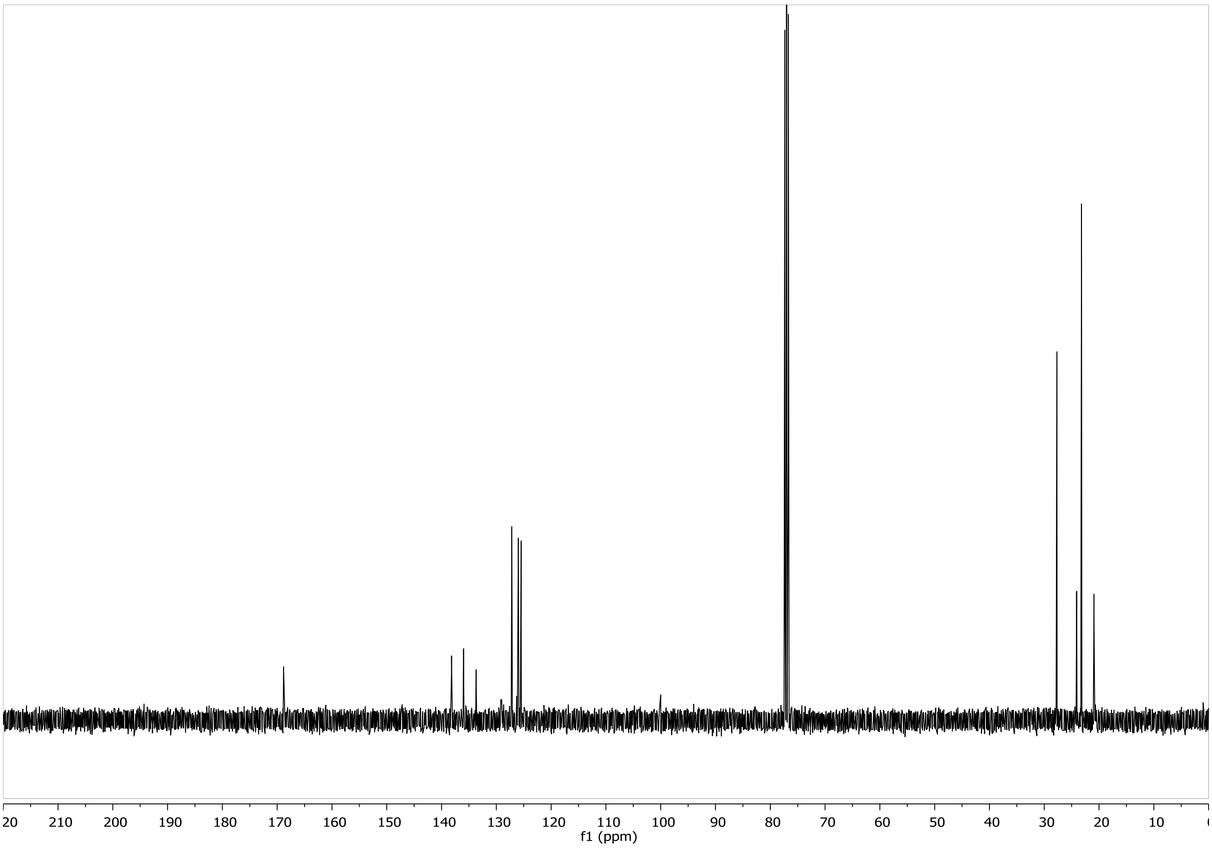
20 210 200 190 180 170 160 150 140 130 120 110 100 90 80 70 60 50 40 30 20 10
f1 (ppm)

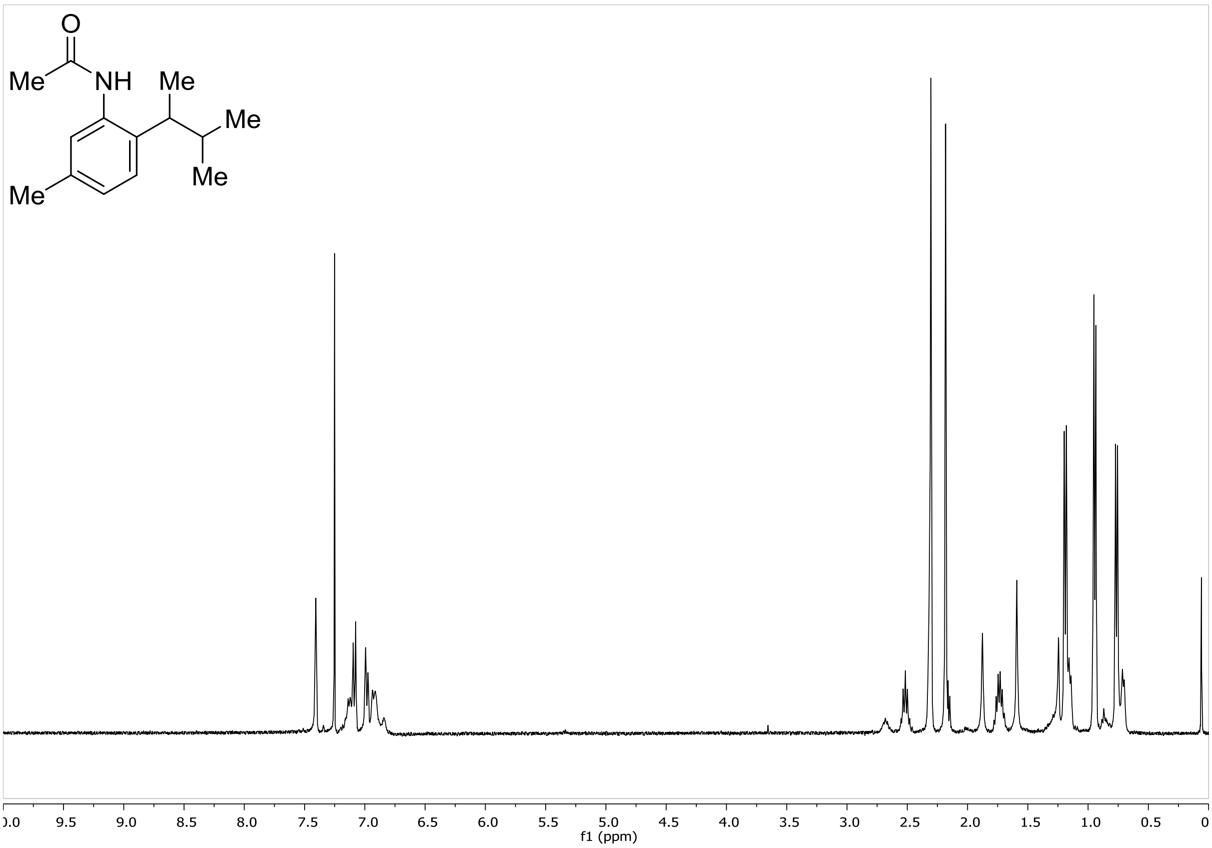
O
Me
NH
Me
Me
Me
Me
f1 (ppm)

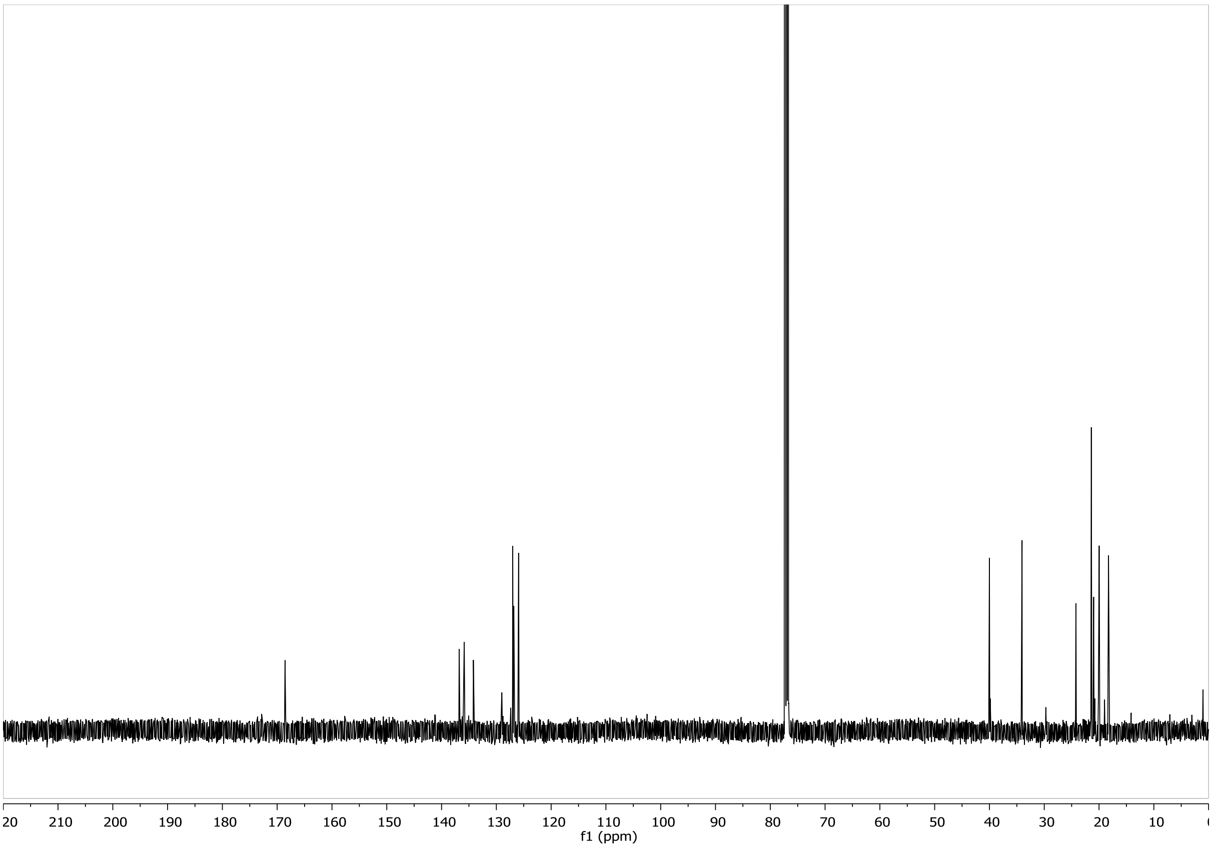
f1 (ppm)

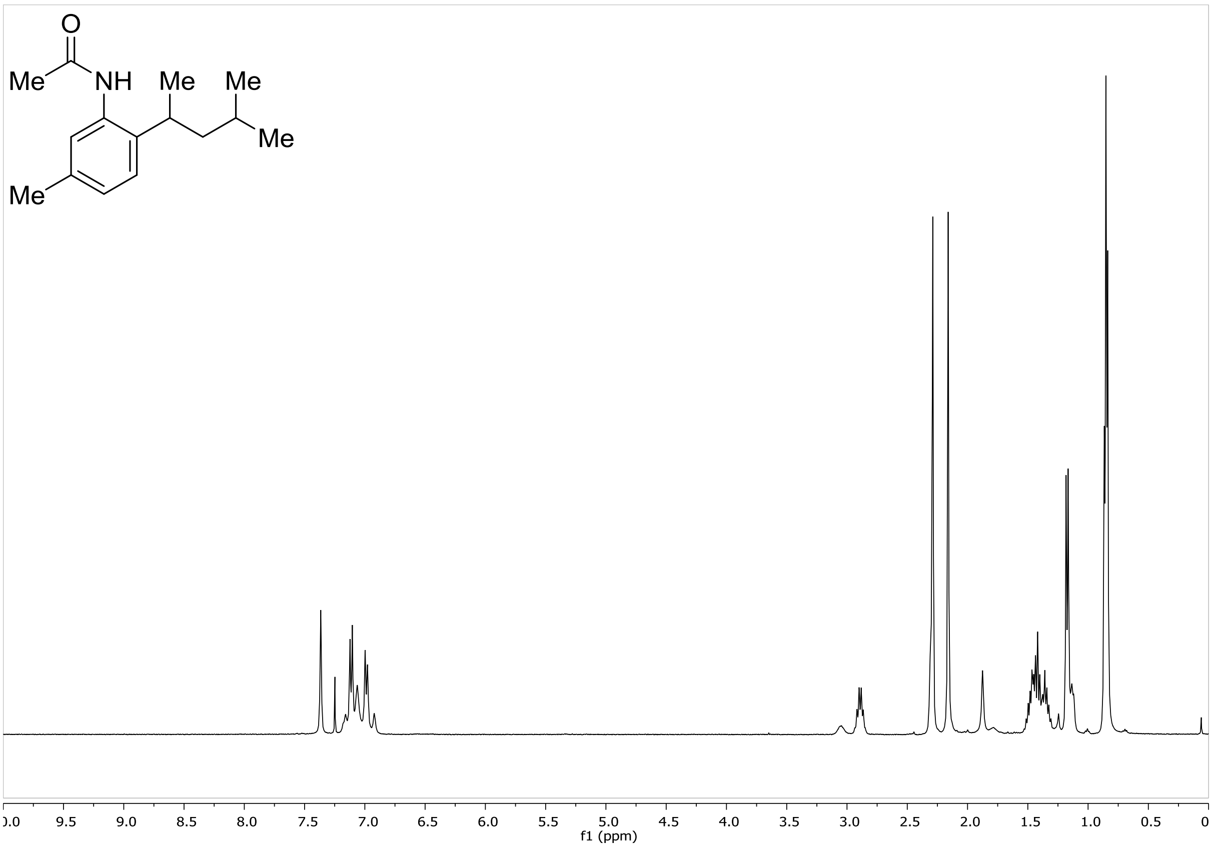
O
Me
NH
Me
Me
Me
Me
f1 (ppm)

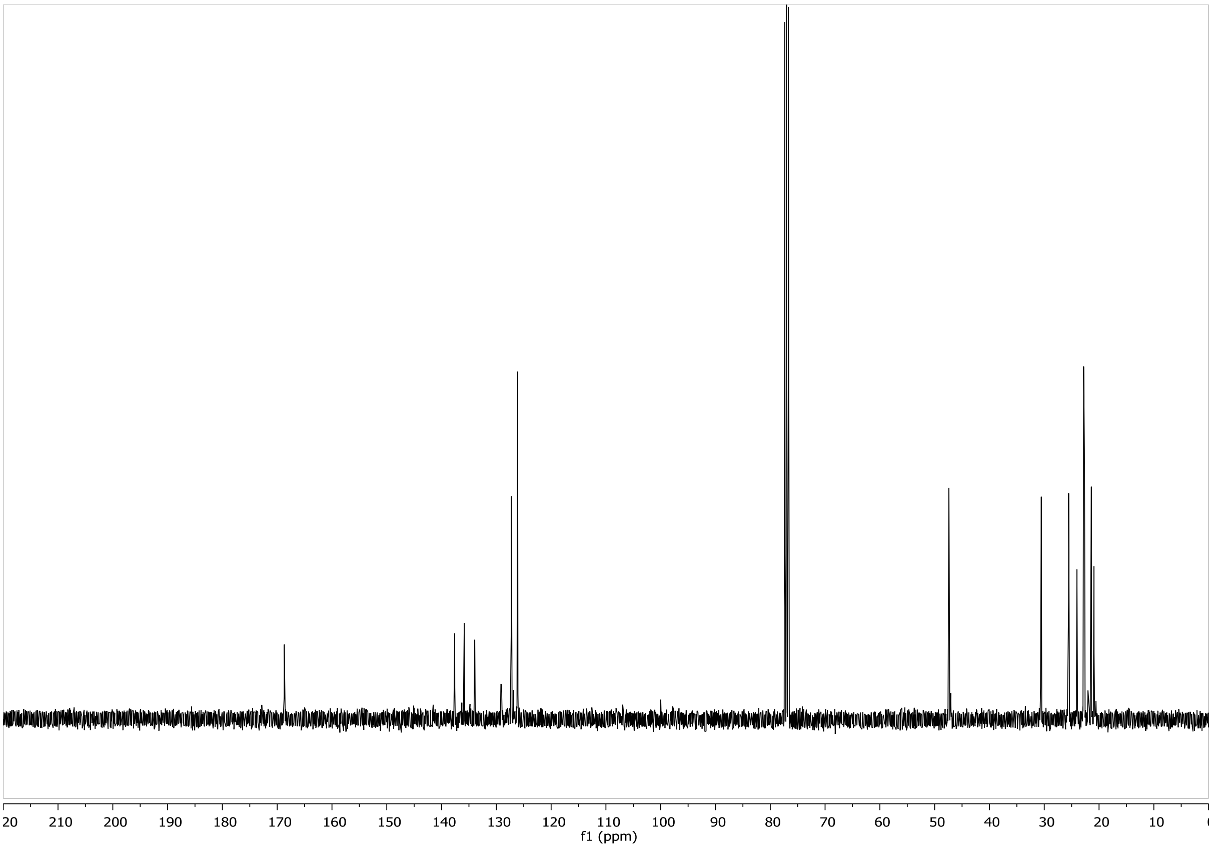
f1 (ppm)

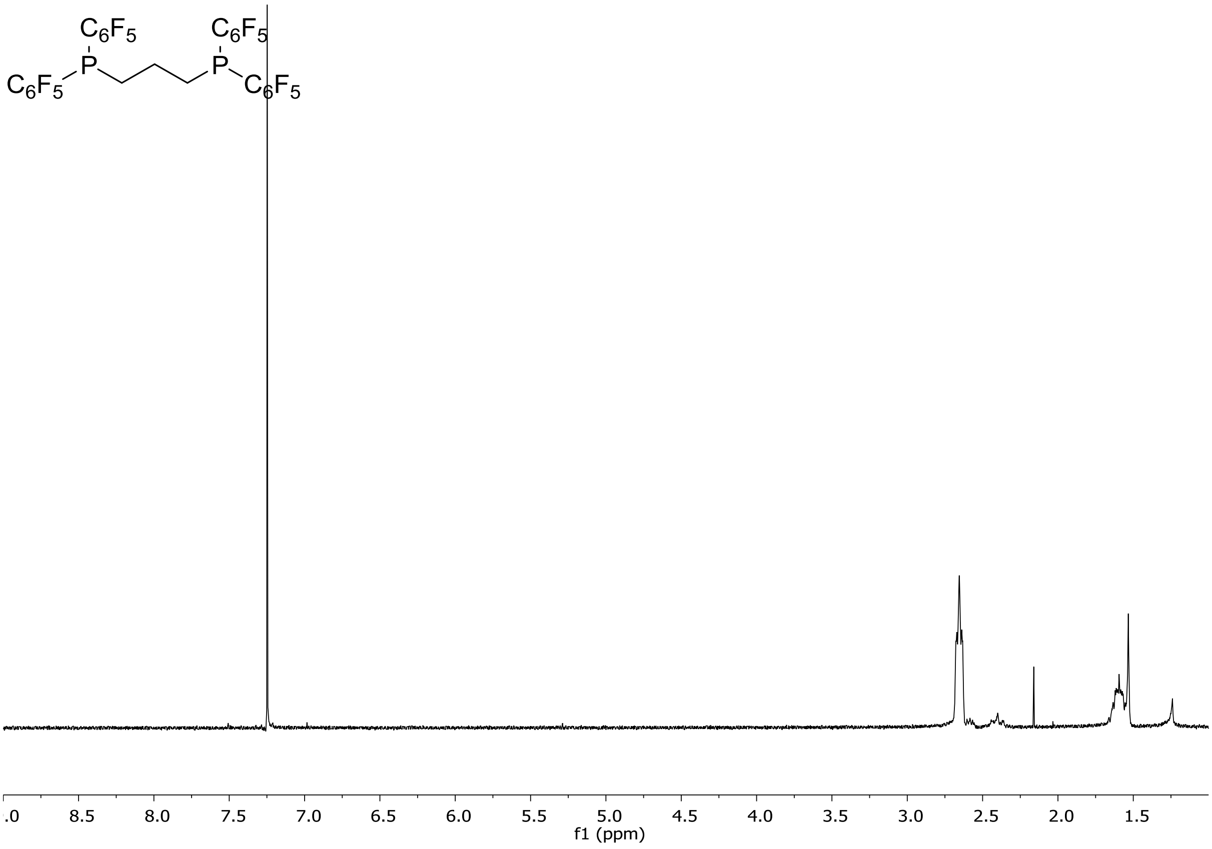
C6F5
P
C6F5
P
C6F5
C6F5
.0 8.5 8.0 7.5 7.0 6.5 6.0 5.5 5.0 4.5 4.0 3.5 3.0 2.5 2.0 1.5
f1 (ppm)

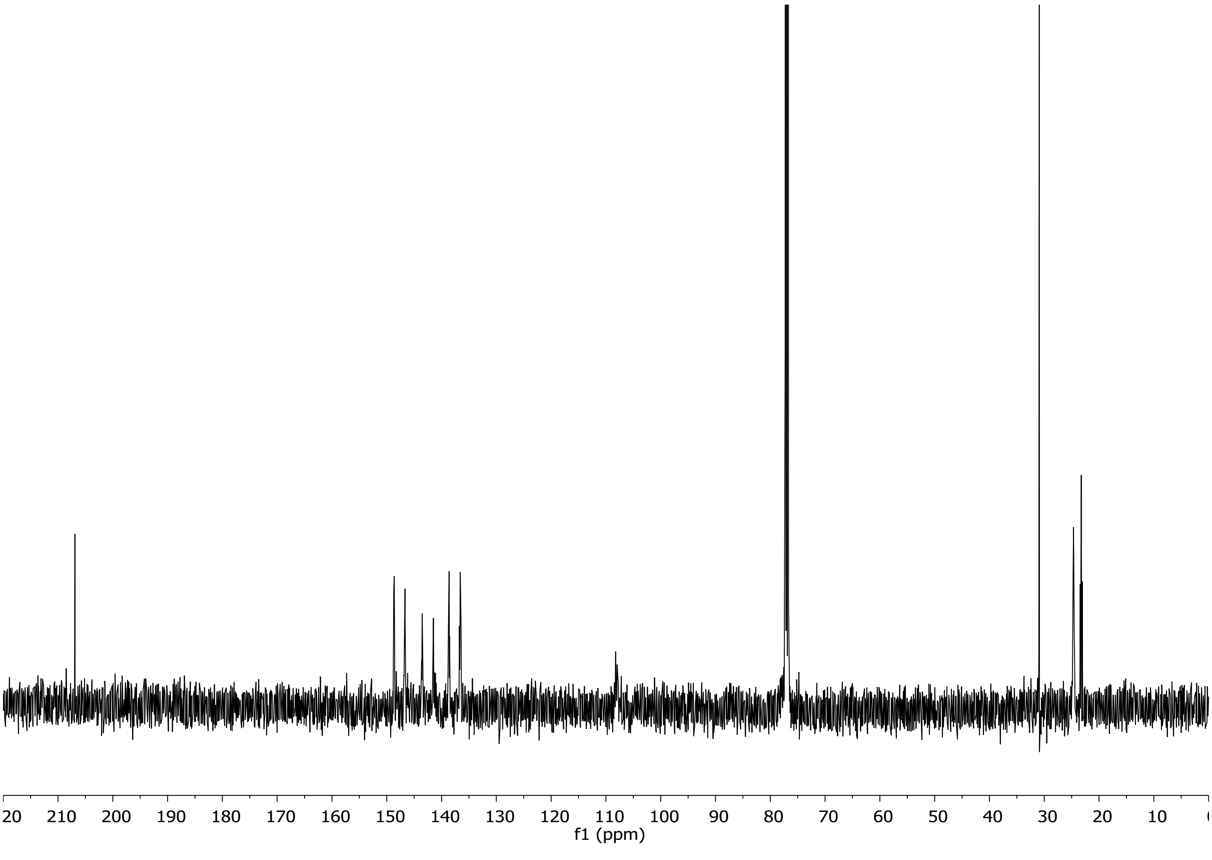
20 210 200 190 180 170 160 150 140 130 120 110 100 90 80 70 60 50 40 30 20 10
f1 (ppm)

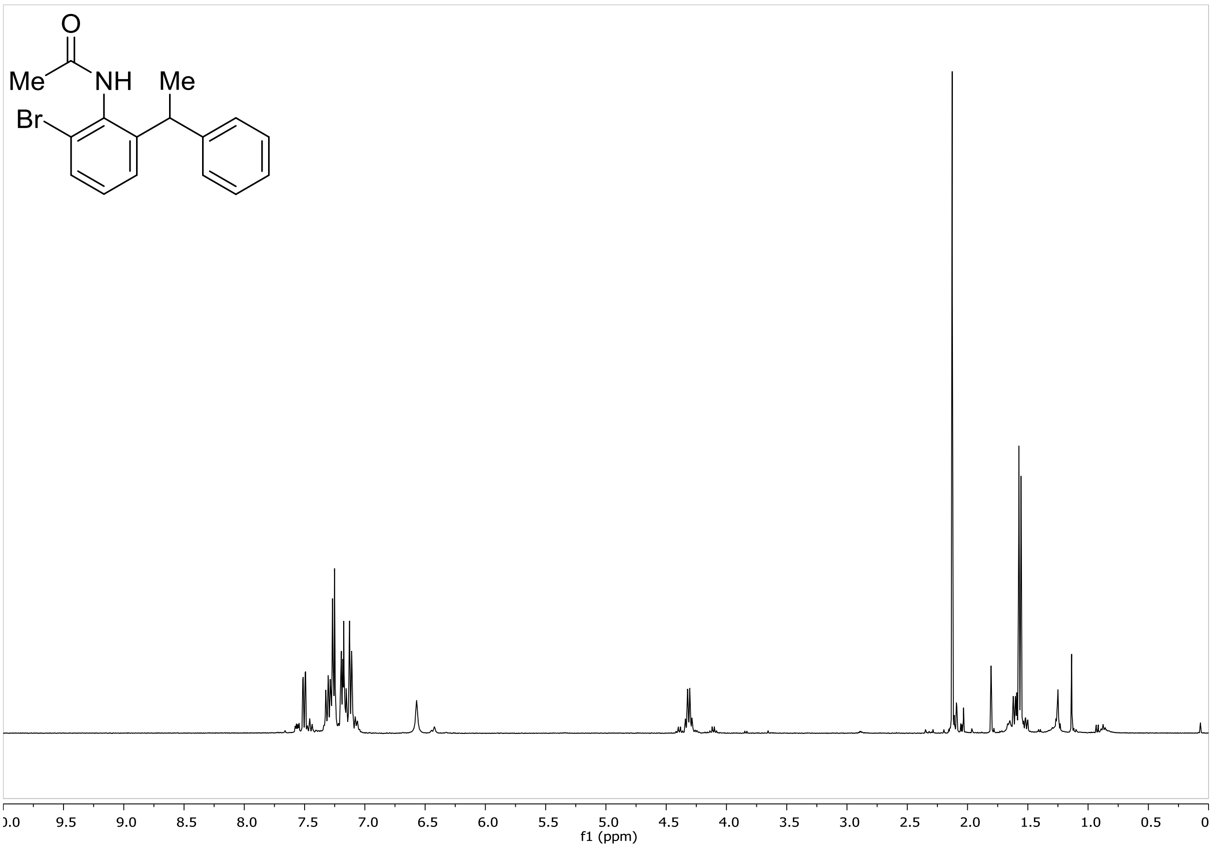
O
Me
NH
Me
Br
9.5 9.0 8.5 8.0 7.5 7.0 6.5 6.0 5.5 5.0 4.5 4.0 3.5 3.0 2.5 2.0 1.5 1.0 0.5 0
f1 (ppm)

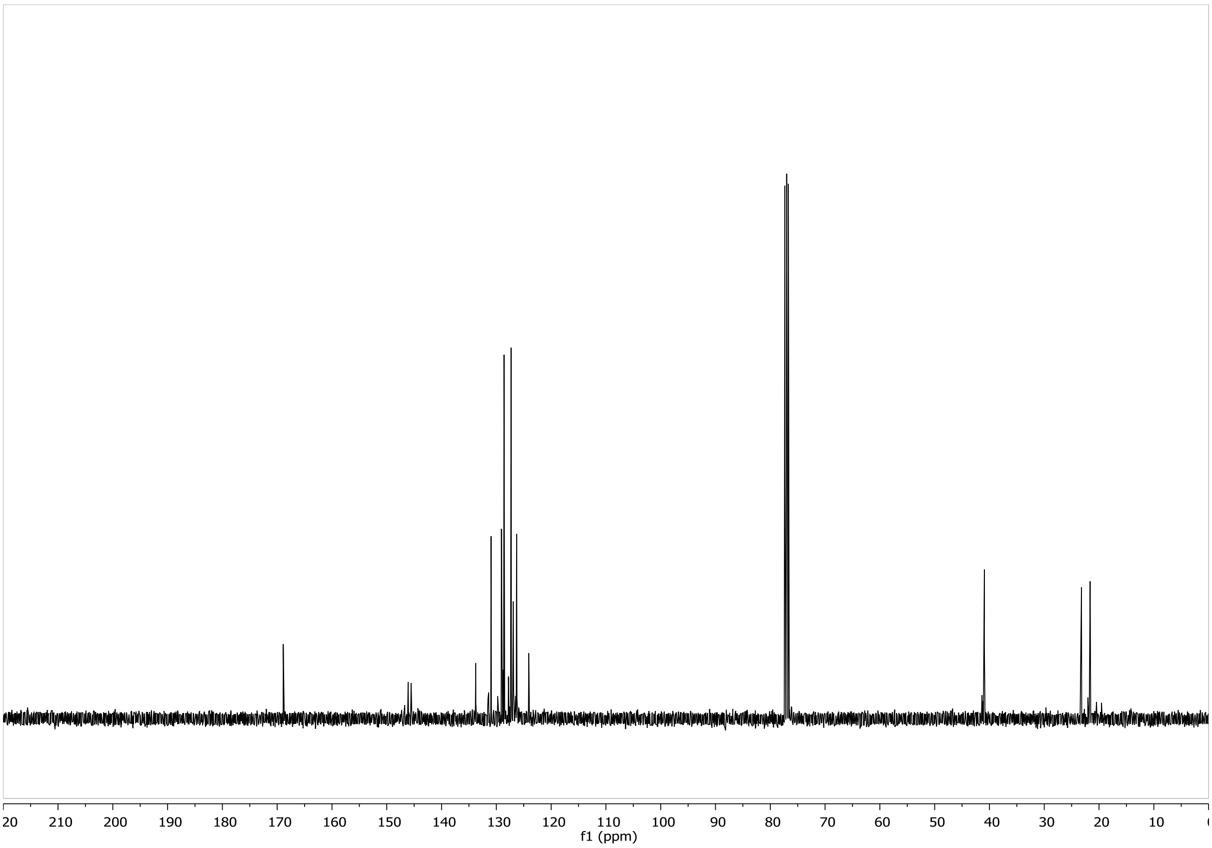
210 200 190 180 170 160 150 140 130 120 110 100 90 80 70 60 50 40 30 20 10
f1 (ppm)

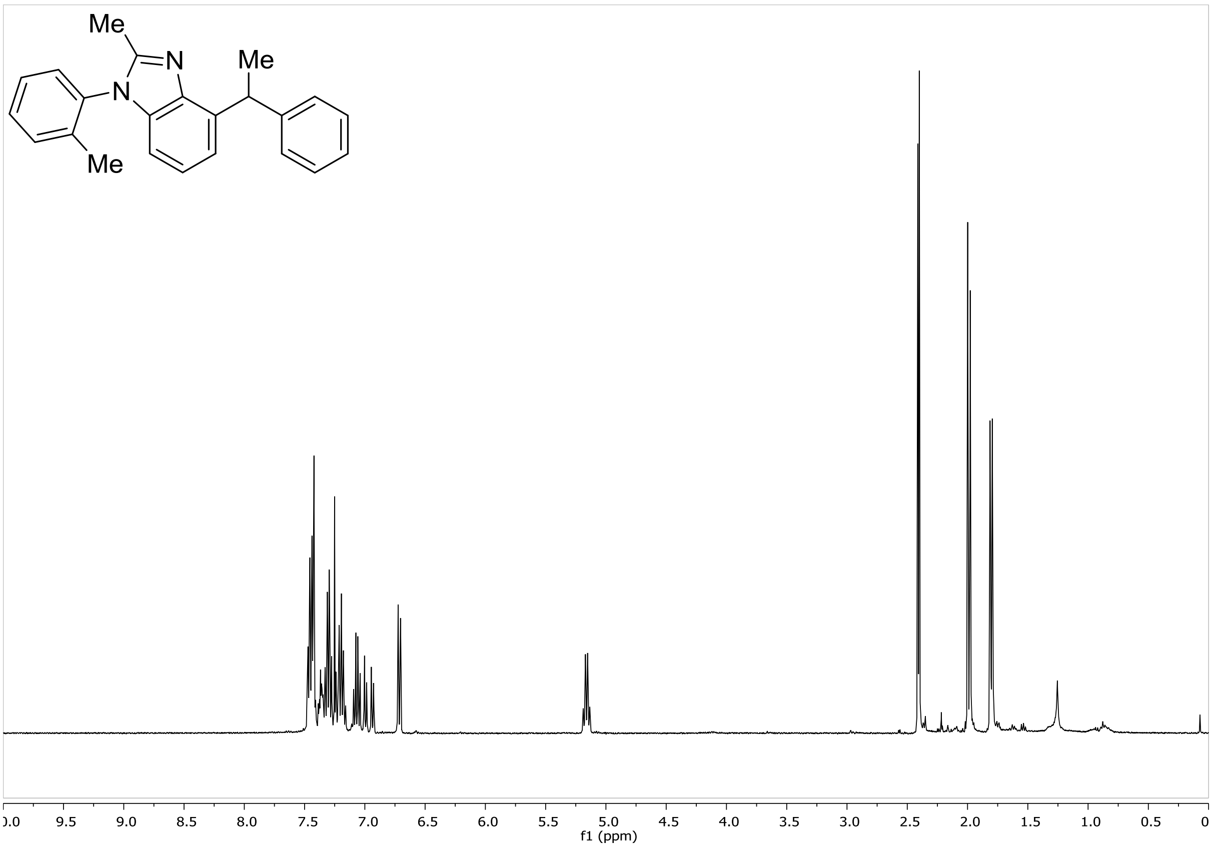
Me
N
Me
N
Me
f1 (ppm)

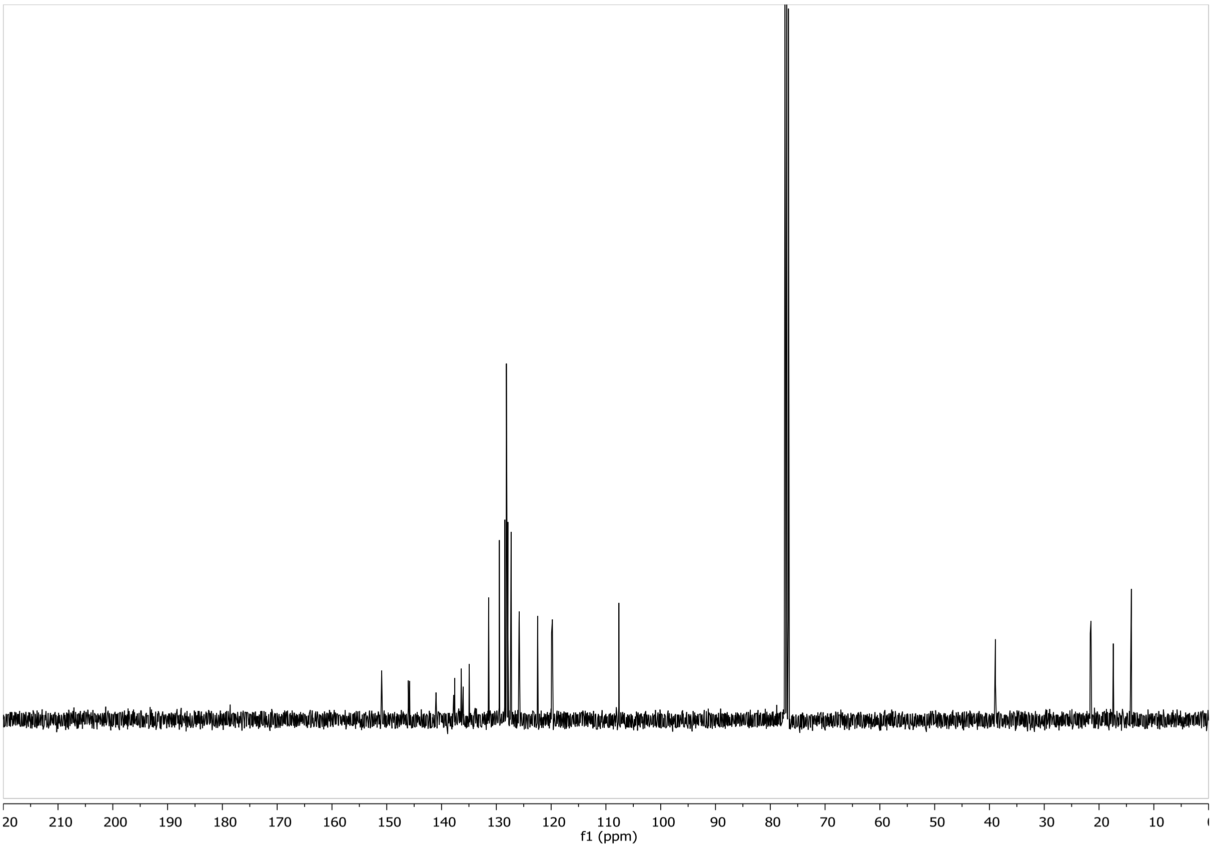
f1 (ppm)

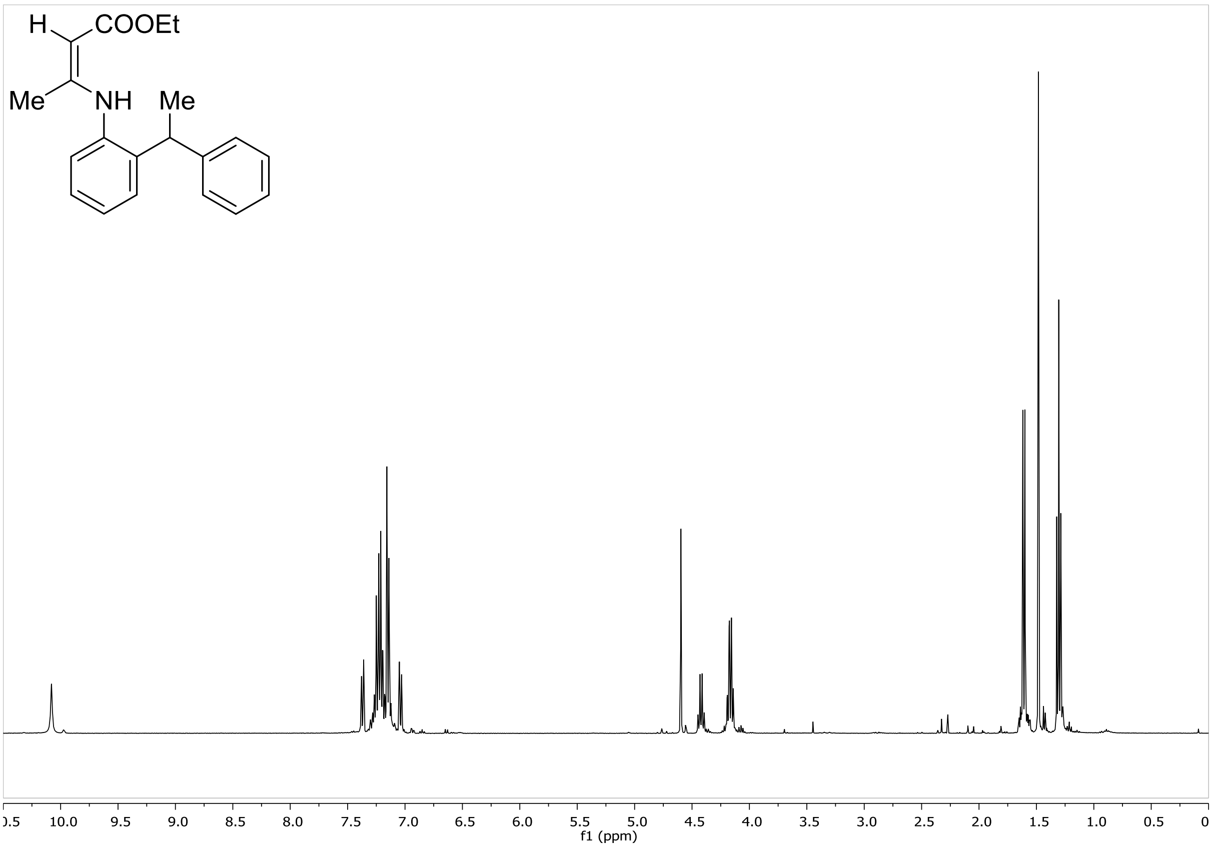
H
COOEt
Me
NH
Me
10.0 9.5 9.0 8.5 8.0 7.5 7.0 6.5 6.0 5.5 5.0 4.5 4.0 3.5 3.0 2.5 2.0 1.5 1.0 0.5
f1 (ppm)

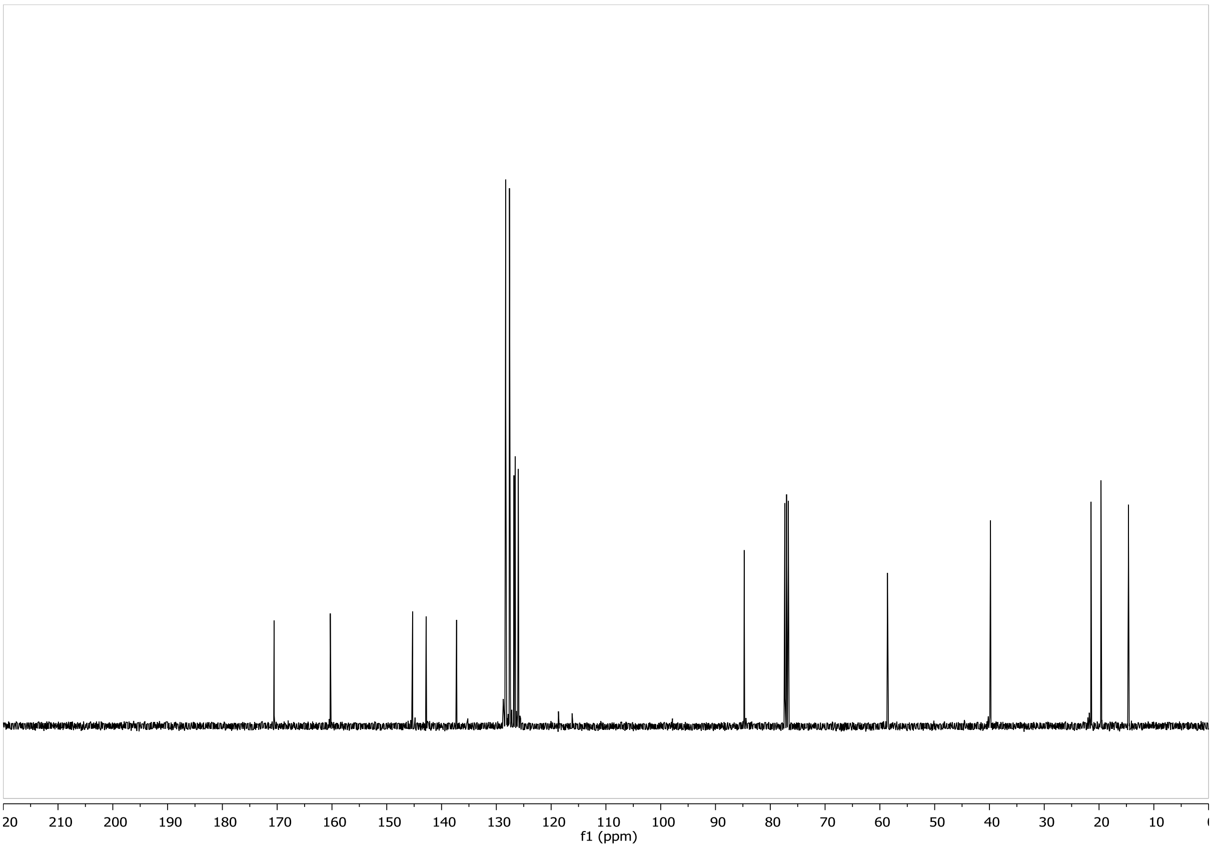
210 200 190 180 170 160 150 140 130 120 110 100 90 80 70 60 50 40 30 20 10
f1 (ppm)

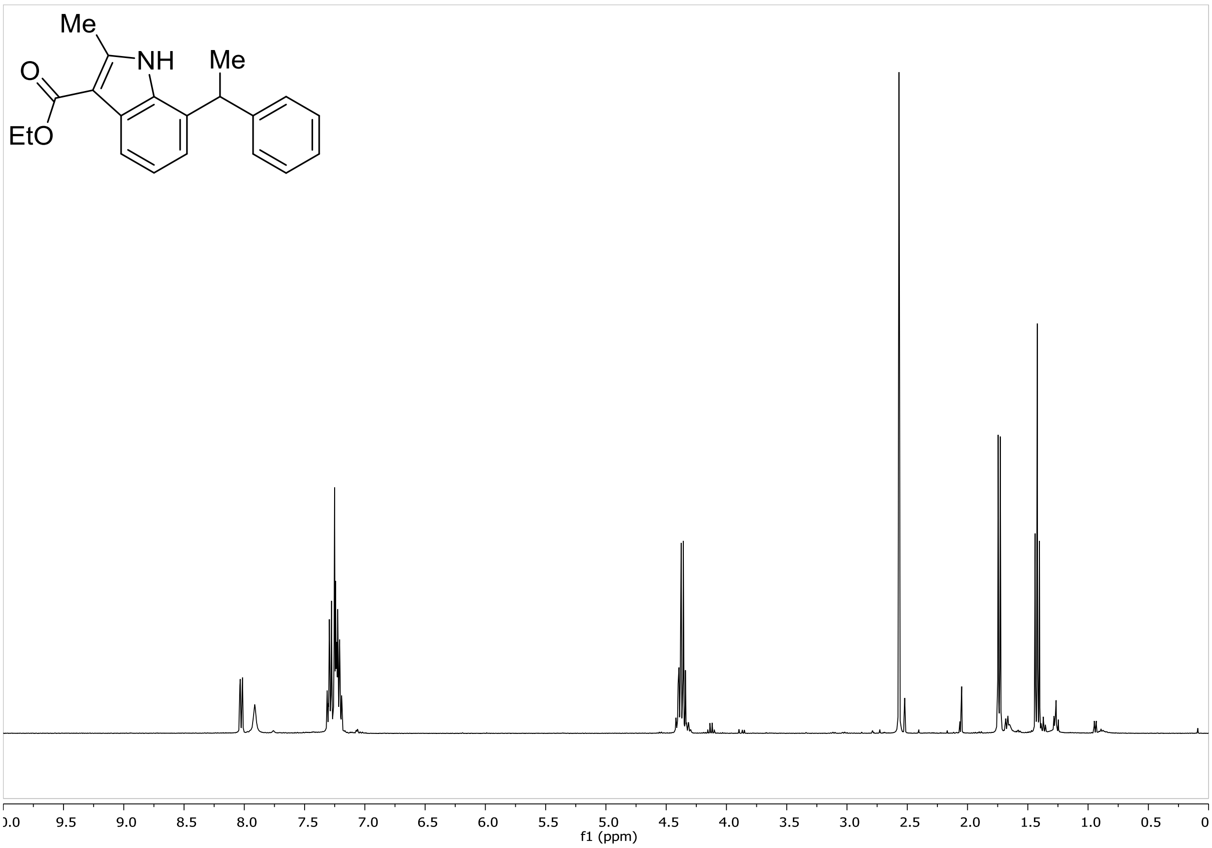
Me
NH
Me
O
EtO
f1 (ppm)

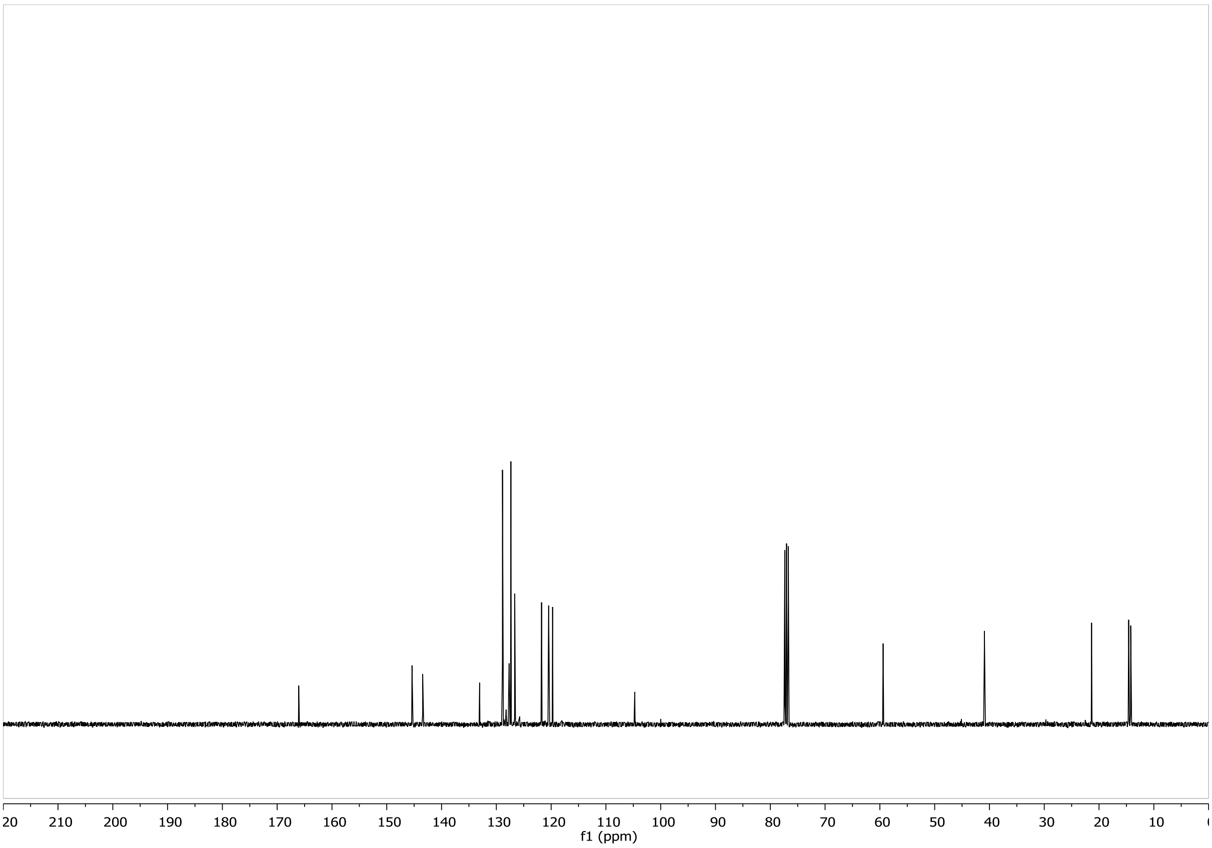
f1 (ppm)

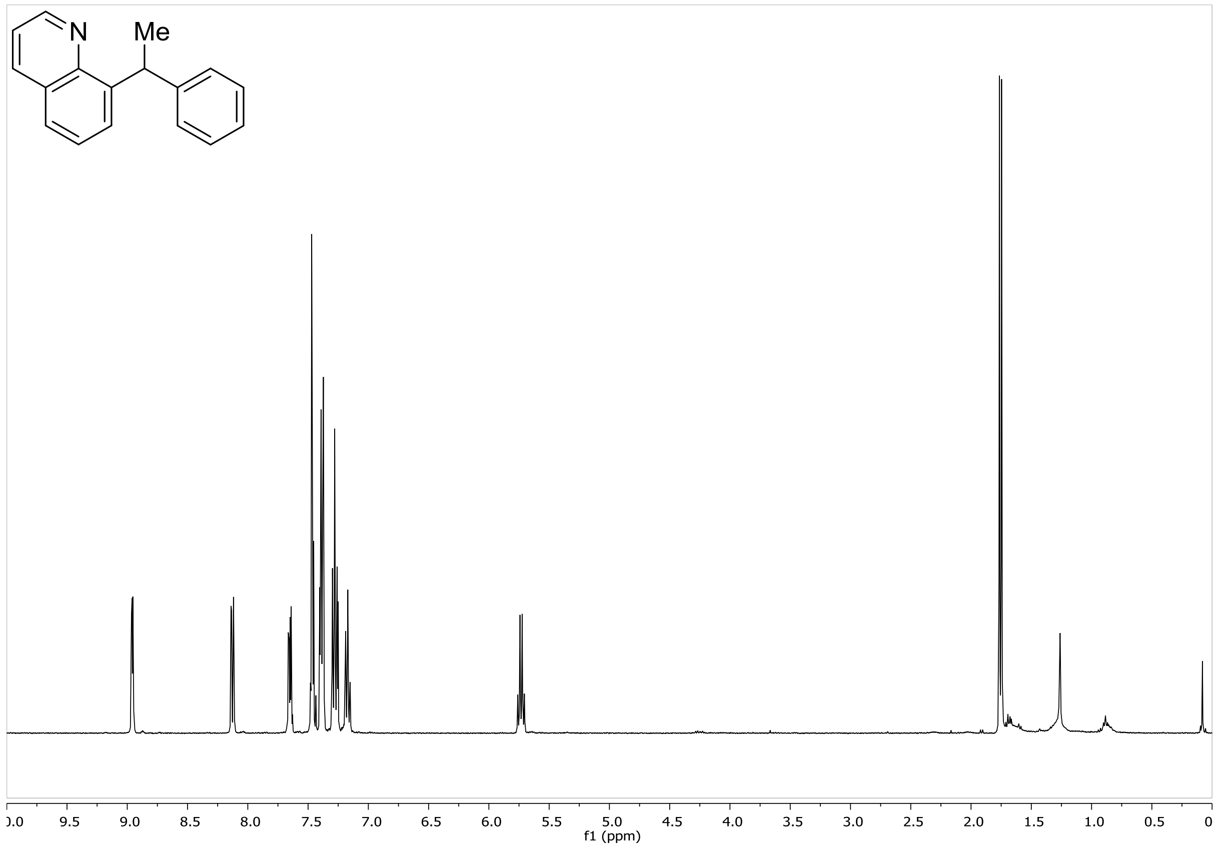
N
Me
f1 (ppm)

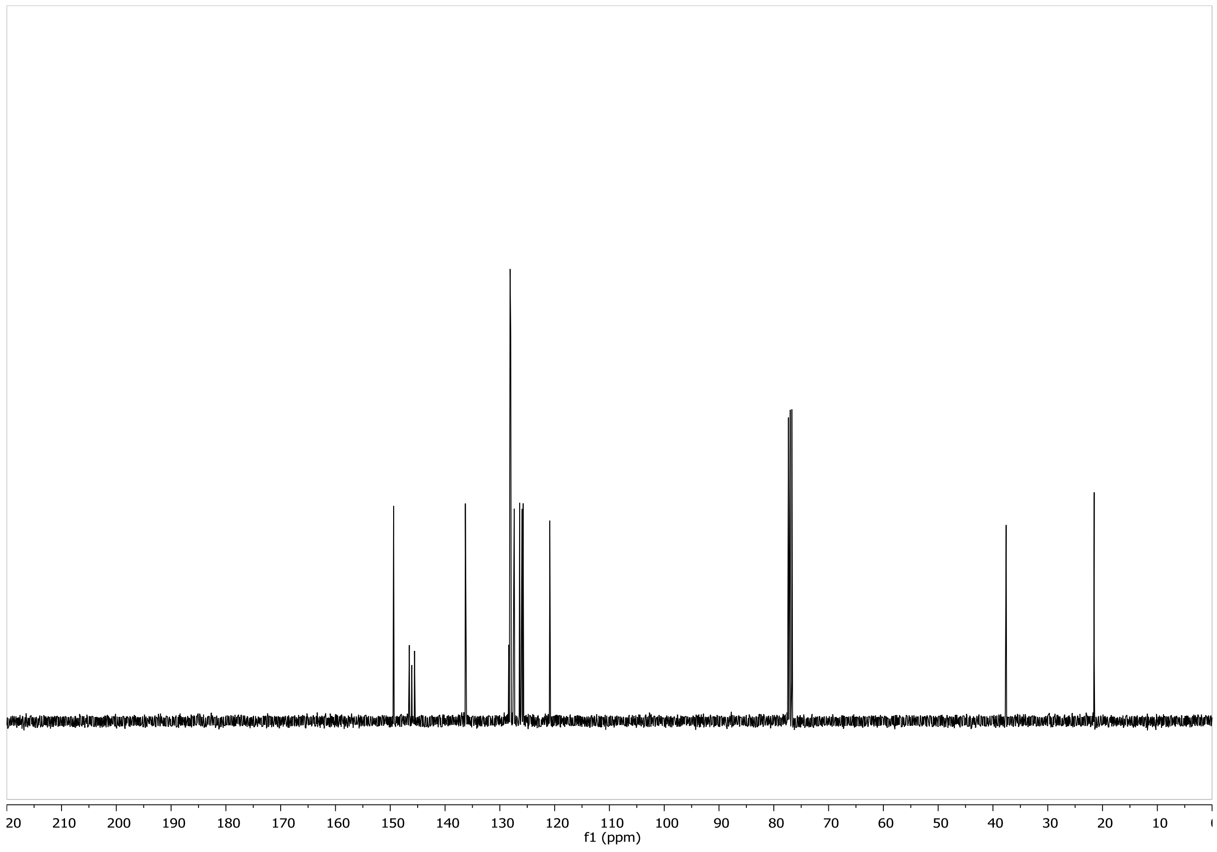
f1 (ppm)

References

1. [a] For **[Ir(cod)$_2$]BARF** and **d^{F}ppb** and **4-methylstyrene-*β,β-d*$_2$**: Crisenza, G. E. M.; McCreanor, N. G.; Bower, J. F. *J. Am. Chem. Soc.* **2014**, *136*, 10258; [b] for **[Ir(cod)$_2$]OTf**: Tsuchikama, K.; Kasagawa, M.; Endo, K.; Shibata, T. *Org. Lett.* **2009**, *11*, 1821.
2. [a] Jensen, T.; Pedersen, H.; Bang-Andersen, B.; Madsen, R.; Jørgensen, M. *Angew. Chem. Int. Ed.* **2008**, *47*, 888; [b] Marburg, S.; Tolman, R. L. *J. Heterocyclic Chem.* **1980**, *17*, 1333.
3. Hesp, K. D.; Bergman, R. G.; Ellman, J. A. *J. Am. Chem. Soc.* **2011**, *133*, 11430.
4. [a] Courtin, A. *Hel. Chim. Acta* **1980**, *63*, 2280; [b] Nguyen, P.; Corpuz, E.; Heidelbaugh, T. M.; Chow, K.; Garst, M. E. *J. Org. Chem.* **2003**, *68*, 10195.
5. Karimi, B.; Behzadnia, H. *Synlett* **2010**, 2019.
6. [a] Zhao, H.; Vandenbossche, C. P.; Koenig, S. G.; Singh, S. P.; Bakale, R. P. *Org. Lett.* **2008**, *10*, 505; [b] Kloetzel, M. C.; King, W.; Wasserman, W. J.; Warren, C. K.; Larssen, P. A. *J. Org. Chem.* **1961**, *26*, 607; [c] Chen, C.-T.; Kuo, J.-H.; Pawar, V. D.; Munot, Y. S.; Weng, S.-S.; Ku, C.-H.; Liu, C.-Y. *J. Org. Chem.* **2005**, *70*, 1188.
7. [a] Hay, M. P.; Hicks, K. O.; Pchalek, K.; Lee, H. H.; Blaser, A.; Pruijn, F. B.; Anderson, R. F.; Shinde, S. S.; Wilson, W. R.; Denny, W. A. *J. Med. Chem.* **2008**, *51*, 6853; [b] Lal, S.; Snape, T. J. *J. Mol. Catal. B: Enzym.* **2012** , *83*, 80.
8. [a] Peet, N. P.; Sunder, S.; Barbuch, R. J.; Whalon, M. R.; Huber, E. W.; Huffman, J. C. *J. Heterocycl. Chem.* **1989**, *26*, 1611; [b] Shimada, T.; Nakamura, I.; Yamamoto, Y. *J. Am. Chem. Soc.* **2004**, *126*, 10546.
9. 1,3-Bis(dichlorophosphanyl)propane was prepared following a literature procedure: Berven, B. M.; Koutsantonis, G. A. *Synthesis* **2008**, 2626.
10. Bedford, R. B.; Haddow, M. F.; Mitchell, C. J.; Webster, R. L. *Angew. Chem. Int. Ed.* **2011**, *50*, 5524.
11. Zheng, N.; Anderson, K. W.; Huang, X.; Nguyen, H. N.; Buchwald, S. L. *Angew. Chem. Int. Ed.* **2007**, *46*, 7509.
12. [a] Hickinbottom, W. J. *J. Am. Chem. Soc.* **1934**, 319; [b] Marcseková, K.; Doye, S. *Synthesis* **2007**, 145.
13. Sridharan, V.; Avendaño, C.; Menéndez, J. C. *Synlett* **2007**, 0881.
14. Neumann, J. J.; Rakshit, S.; Dröge, T.; Würtz, S.; Glorius, F. *Chem. Eur. J.* **2011**, *17*, 7298.
15. Biao, L.; Li, C.; Zhang, L. *J. Am. Chem. Soc.* **2010**, *132*, 14070.
